# Burosumab for the Treatment of Tumor‐Induced Osteomalacia

**DOI:** 10.1002/jbmr.4233

**Published:** 2021-01-12

**Authors:** Suzanne M Jan de Beur, Paul D Miller, Thomas J Weber, Munro Peacock, Karl Insogna, Rajiv Kumar, Frank Rauch, Diana Luca, Tricia Cimms, Mary Scott Roberts, Javier San Martin, Thomas O Carpenter

**Affiliations:** ^1^ Johns Hopkins University School of Medicine Baltimore MD USA; ^2^ Colorado Center for Bone Research Lakewood CO USA; ^3^ Duke University Durham NC USA; ^4^ Indiana University School of Medicine Indianapolis IN USA; ^5^ Yale University School of Medicine New Haven CT USA; ^6^ Mayo Clinic College of Medicine Rochester MN USA; ^7^ McGill University Montreal Canada; ^8^ Ultragenyx Pharmaceutical Inc. Novato CA USA

**Keywords:** BONE HISTOMORPHOMETRY, TUMOR‐INDUCED BONE DISEASE, CLINICAL TRIALS, OSTEOMALACIA AND RICKETS, PTH/VIT D/FGF23

## Abstract

Tumor‐induced osteomalacia (TIO) is caused by phosphaturic mesenchymal tumors producing fibroblast growth factor 23 (FGF23) and is characterized by impaired phosphate metabolism, skeletal health, and quality of life. UX023T‐CL201 is an ongoing, open‐label, phase 2 study investigating the safety and efficacy of burosumab, a fully human monoclonal antibody that inhibits FGF23, in adults with TIO or cutaneous skeletal hypophosphatemia syndrome (CSHS). Key endpoints were changes in serum phosphorus and osteomalacia assessed by transiliac bone biopsies at week 48. This report focuses on 14 patients with TIO, excluding two diagnosed with X‐linked hypophosphatemia post‐enrollment and one with CSHS. Serum phosphorus increased from baseline (0.52 mmol/L) and was maintained after dose titration from week 22 (0.91 mmol/L) to week 144 (0.82 mmol/L, *p* < 0.0001). Most measures of osteomalacia were improved at week 48: osteoid volume/bone, osteoid thickness, and mineralization lag time decreased; osteoid surface/bone surface showed no change. Of 249 fractures/pseudofractures detected across 14 patients at baseline, 33% were fully healed and 13% were partially healed at week 144. Patients reported a reduction in pain and fatigue and an increase in physical health. Two patients discontinued: one to treat an adverse event (AE) of neoplasm progression and one failed to meet dosing criteria (receiving minimal burosumab). Sixteen serious AEs occurred in seven patients, and there was one death; all serious AEs were considered unrelated to treatment. Nine patients had 16 treatment‐related AEs; all were mild to moderate in severity. In adults with TIO, burosumab exhibited an acceptable safety profile and was associated with improvements in phosphate metabolism and osteomalacia. © 2020 The Authors. *Journal of Bone and Mineral Research* published by American Society for Bone and Mineral Research..

## Introduction

Tumor‐induced osteomalacia (TIO) is an ultrarare disease caused by tumors secreting fibroblast growth factor 23 (FGF23).^(^ ^1^ ^)^ Most patients have phosphaturic mesenchymal tumors, which are often small and occur in soft tissue or bone, making localization difficult and delaying diagnosis. The excess levels of FGF23 in TIO lead to impaired renal phosphate reabsorption, reduced active vitamin D synthesis, and chronic hypophosphatemia. Clinical manifestations include osteomalacia, fractures, musculoskeletal pain, fatigue, severe myopathy, and reduced health‐related quality of life, which typically result in rapid clinical deterioration of the patient.

Complete surgical resection of the causative tumor is curative and the established treatment for TIO.^(^ ^1^ ^)^ Incomplete resection often results in tumor recurrence with symptoms. Roughly 35% to 40% of tumors cannot be localized.^(^ ^2^, ^3^ ^)^ When the tumor cannot be localized and completely resected, supplementation with multiple daily doses of oral phosphate and active vitamin D analogues is required. However, efficacy of supplementation therapy can be limited. Complications of supplementation therapy include frequent gastrointestinal (GI) distress, hypercalcemia, nephrocalcinosis, and tertiary hyperparathyroidism.

Burosumab is a fully human monoclonal antibody against FGF23. By blocking excess FGF23, burosumab normalized phosphate metabolism, improved bone health, and ameliorated clinical symptoms in both children and adults with X‐linked hypophosphatemia (XLH). Burosumab is approved for treatment of XLH and TIO.^(^ ^4^, ^5^, ^6^, ^7^, ^8^, ^9^ ^)^ In this first report of a long‐term prospective trial in TIO, we present the primary analysis of a phase 2 clinical trial investigating the efficacy and safety of burosumab in adults with TIO.

## Materials and Methods

### Patients

Adults (aged ≥18 years) with TIO not curable by surgical excision or cutaneous skeletal hypophosphatemia syndrome (CSHS) were eligible for this trial. CSHS is rare condition caused by somatic *RAS* mutations often accompanied by skin lesions and skeletal dysplasia with excess FGF23.^(^ ^10^ ^)^ Additional key inclusion criteria were fasting serum phosphorus and maximum tubular phosphate reabsorption per unit of glomerular filtration rate (TmP/GFR), both <0.81 mmol/L; intact FGF23 ≥100 pg/mL (Kainos ELISA, not commercially available); and a corrected serum calcium <10.8 mg/dL. Key exclusion criteria were use of vitamin D analogues or oral phosphate salts within 2 weeks of screening or during the study; use of medication to suppress parathyroid hormone (PTH) within 2 months before screening; and history of malignancy within 5 years of screening, with the exception of phosphaturic mesenchymal tumors or nonmelanoma skin cancers. Additional criteria are in the supplemental materials.

### Study design

This ongoing, open‐label, single‐arm, phase 2 study (UX023T‐CL201) is investigating the efficacy and safety of burosumab in adults with TIO and CSHS at six US sites. Patients were treated with burosumab subcutaneously every 4 weeks for up to 144 weeks at the time of this report. Dosing started at 0.3 mg/kg and was titrated through week 16, and beyond if needed, to a maximum of 2.0 mg/kg every 4 weeks to achieve a fasting peak serum phosphorus level between 0.81 and 1.29 mmol/L. At baseline and week 48, transiliac bone biopsies—horizontal full‐thickness biopsies of the ilium from a site 2 cm dorsal of the anterior superior iliac spine—were obtained using a needle with an inner diameter of ≥5 mm. The bone biopsy was not required at baseline if a previous bone biopsy taken within 12 months of screening confirmed the diagnosis of osteomalacia, the patients' clinical manifestations have not changed significantly since the time of the previous biopsy/diagnosis, and the tissue collected at that biopsy was made available for testing for this study. Patients received two 3‐day courses of tetracycline‐HCl (or demeclocycline‐HCl) 20 and 8 days before each biopsy for dynamic histomorphometric analysis, as previously described.^(^ ^11^, ^12^ ^)^ For structural morphometric analysis, biopsies were stained using Masson Goldner Trichrome.^(^ ^11^, ^12^ ^)^

### Study outcomes

Co‐primary endpoints were (i) the proportion of patients with an average fasting serum phosphorus level at the midpoint of the dose interval between baseline and week 24 >0.81 mmol/L; and (ii) change from baseline in parameters of osteomalacia at week 48, as assessed by osteoid thickness, osteoid surface/bone surface, osteoid volume/bone volume, and mineralization lag time. In patients in whom the mineralization defect was profound and, therefore, the uptake of tetracycline label very low, mineralization lag time was calculated using imputation.^(^ ^13^ ^)^ Key pharmacodynamic endpoints include change from baseline in fasting TmP/GFR, 1,25(OH)_2_D, and bone‐specific alkaline phosphatase (BALP). A full list of assessments and schedule is in the supplemental materials.

Fractures and pseudofractures were identified at baseline by a technetium‐labeled (^99m^Tc) methyl diphosphonate (MDP) whole‐body bone scan, and fracture healing was assessed by follow‐up ^99m^Tc‐MDP whole‐body bone scans at weeks 24, 48, 96, and 144. Pre‐ and post‐treatment scans were compared by a central reader who was blinded to time point and patient data. Although bone scans are highly sensitive for detecting mineralizing lesions with increased uptake at sites of injury and ongoing repair, they have a low diagnostic specificity and are typically interpreted in the context of the patient's medical condition. In the setting of osteomalacia and TIO, bone scan findings are very informative in providing a comprehensive profile of the underlying skeletal disease.^(^ ^14^ ^)^ A number of studies have demonstrated the increased sensitivity of bone scans in detecting fractures/pseudofractures compared with standard radiographs, particularly in the setting of osteomalacia.^(^ ^15^ ^)^ Functional mobility was assessed by the sit‐to‐stand test^(^ ^16^ ^)^ and the 6‐minute walk test.^(^ ^17^ ^)^ The 36‐item Short Form Health Survey (SF‐36v2) was used to assess patient‐reported physical and mental health–related quality of life.^(^ ^18^ ^)^ Patient‐reported pain and fatigue were assessed with the Brief Pain Inventory and the Brief Fatigue Inventory, respectively.^(^ ^19^, ^20^, ^21^ ^)^

Key safety assessments include the incidence and severity of adverse events (AE) and serious adverse events, development of anti‐burosumab antibodies, changes in echocardiograms and electrocardiograms, renal ultrasound nephrocalcinosis scores, fasting serum calcium, plasma intact PTH (iPTH), and 24‐hour urine calcium excretion. Patients with an identifiable tumor undergo CT or MRI scanning, depending on the method of initial identification, every 6 months to monitor tumor size. During the study, ^68^Ga‐DOTATATE PET/CT scans were added to the protocol for any patients who did not have a tumor identified by other means.

### Study oversight

This study was designed, conducted, recorded, and reported in accordance with the principles established by the World Medical Association Declaration of Helsinki Ethical Principles for Medical Research Involving Human Patients. Institutional review boards at each site approved the protocol. Investigators obtained written informed consent from each participant. The study is registered with ClinicalTrials.gov (NCT02304367).

### Statistical analysis

Statistical analyses were conducted in SAS version 9.4 (SAS Institute, Cary, NC, USA). We provide descriptive summaries for efficacy and safety endpoints. Continuous variables are summarized by number of patients, mean, standard error (SE), standard deviation (SD), median, minimum, and maximum. Categorical data are summarized as number or percentage of patients. Changes in histomorphometric endpoints are analyzed using a two‐sided *t* test, providing the 95% confidence interval (CI) and *p* value. For other selected endpoints, the least squares (LS) mean and SE for the change from baseline are provided using the generalized estimating equation (GEE) repeated‐measures analysis, including time as the categorical variable adjusted for baseline measurement in the model with compound symmetry covariance structure. No adjustments for multiplicity were made.

## Results

### Disposition

Of the 20 patients screened, 17 were enrolled: 14 had TIO, one had CSHS, and two who enrolled with diagnoses of TIO were subsequently diagnosed with X‐linked hypophosphatemia after enrollment (Supplemental Fig. S1). These two patients did not have a tumor localized at enrollment. Upon review of medical history, these patients had a clinical profile consistent with X‐linked hypophosphatemia, including symptoms since childhood. These patients were subsequently diagnosed with X‐linked hypophosphatemia based on *PHEX* genetic testing. Four other patients had tumors that had never been located. However, these patients did not undergo *PHEX* testing because their clinical profile was consistent with TIO (normal height and late onset of symptoms). Figures, tables, and text summarize group findings for patients with TIO only. Results for the two patients with X‐linked hypophosphatemia were similar to observations in previous clinical trials.^(^ ^7^, ^9^, ^12^ ^)^ The source of FGF23 in CSHS is unknown and the disease may have a distinct natural history from TIO.^(^ ^10^ ^)^ Because only one patient with CSHS enrolled in this study, results for this patient are provided separately to allow robust descriptions of both disease states (manuscript describing CSHS patient in preparation).

One of the 14 patients with TIO discontinued burosumab after 20 weeks to treat neoplasm progression and subsequently discontinued from the study. Eleven patients with TIO completed a bone biopsy at baseline and week 48 and are included in the bone biopsy analysis. After week 48, a patient with TIO died from cardiac arrest after sepsis, unrelated to burosumab, and a second patient with TIO discontinued from the study because he did not meet serum phosphorus dosing criteria (receiving minimal burosumab dosing; Supplemental Results). Finally, one patient with TIO enrolled late, completing all assessments through week 124 at the time of this analysis. Ten patients with TIO completed 144 weeks of treatment; their pharmacodynamic and patient‐reported outcomes are summarized through week 144.

### Baseline characteristics

At baseline, patients with TIO demonstrated low serum phosphorus, TmP/GFR, and 1,25(OH)_2_D levels (Table 1). Mean (SD) dose of burosumab was at 0.3 (0) mg/kg at baseline, 0.8 (0.4) mg/kg at week 16, 0.9 (0.5) mg/kg at week 48, 0.8 (0.5) mg/kg at week 96, and 0.7 (0.6) mg/kg at week 144.

**Table 1:** Baseline Characteristics

Characteristic, statistic	TIO (*N* = 14)
Male, *n* (%)	8 (57)
Age (years)	56.9 ± 10.3
Years since diagnosis	13.7 ± 13.0
Body mass index (kg/m^2^)	33.8 ± 7.5
Race	
White	12 (86)
Black or African American	2 (14)
Serum phosphorus (mmol/L)	0.52 ± 0.15
Serum FGF23 (pg/mL)	416 (94, 2569)
TmP/GFR (mmol/L)	0.36 ± 0.17
1,25(OH)_2_D (pmol/L)	68 ± 23
25(OH)D (nmol/L)	77 ± 29
Serum iPTH (pmol/L)	9 (1, 54)
Received prior phosphate treatment	13 (93)
Received prior active vitamin D	14 (100)
History of hyperparathyroidism	2 (14)
History of nephrolithiasis	3 (21)
Tumor located at baseline^a^	6 (43)

TIO = tumor‐induced osteomalacia; FGF23 = fibroblast growth factor 23; TmP/GFR = renal tubular reabsorption of phosphate; iPTH = intact parathyroid hormone.

Data are presented as mean ± SD, median (min, max), or *n* (%). Normal reference range is 0.81 to 1.5 mmol/L for serum phosphorus, 0.81 to 1.5 mmol/L for TmP/GFR, 42 to 169 pmol/L for 1,25(OH)_2_D, and 2 to 7 pmol/mL for iPTH. The minimum value for serum FGF23 (94 pg/mL) was below the inclusion criteria cut‐off of 100 pg/mL because this patient was screened before the protocol was amended to include this cut‐off.

^a^ Four patients had tumors that had never been located; 4 had tumors that had been located pre‐study but that could not be located at baseline.

### Efficacy

**Fig 1:**
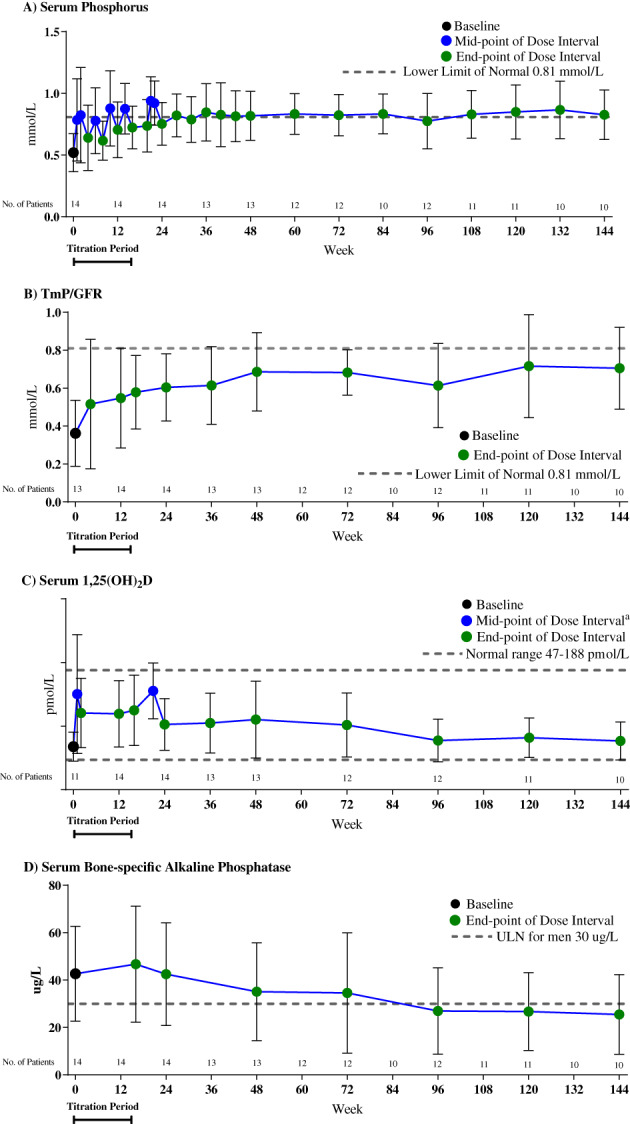
Pharmacodynamic assessments of (*A*) serum phosphorus; (*B*) renal tubular reabsorption of phosphate (TmP/GFR); (*C*) serum 1,25(OH)_2_D; and (*D*) serum bone‐specific alkaline phosphatase. Data are presented as mean ± standard deviation. Baseline data points are shown in black, endpoint of the dose interval data points are shown in green, and midpoint of the dose interval data points are shown in blue, with the exception of week 21 for 1,25(OH)_2_D shown in blue, which is neither the mid‐ nor endpoint of the dose cycle. Assessments in mass units are available in Supplemental Fig. S2.

Seven (50%) patients achieved a mean serum phosphorus above the lower limit of normal (0.81 mmol/L) when the levels at the midpoint of dose intervals were averaged across baseline and week 24 (a co‐primary endpoint). This includes the 16‐week titration period; the mean ± SE serum phosphorus level was 0.85 ± 0.6 mmol/L. At week 22, 12 (86%) patients achieved a fasting serum phosphorus level ≥0.81 mmol/L. Serum phosphorus levels increased at the first post‐baseline assessment (week 1 LS mean change ± SE 0.24 ± 0.77 mmol/L) and were maintained through week 144 (0.33 ± 0.07 mmol/L; *p* < .0001; Fig. 1*A*, Supplemental Fig. S2*A*).

Mean ± SE TmP/GFR level increased from 0.36 ± 0.05 mmol/L at baseline to 0.60 ± 0.05 mmol/L at week 24 (Fig. 1*B*, Supplemental Fig. S2*B*), reflecting an LS mean percentage change of 89% (*p* < .0001); increases were maintained through week 144.

Mean ± SE serum 1,25(OH)_2_D level increased from 68 ± 7 pmol/L at baseline to 103 ± 11 pmol/L at week 24 (Fig. 1*C*, Supplemental Fig. S2*C*), reflecting an LS mean percentage change of 56% (*p* < .01). After week 24, serum 1,25(OH)_2_D levels declined toward baseline levels, likely mirroring the normalization in serum phosphorus levels, while remaining within the normal range (47 to 187 pmol/L).

Serum BALP level increased slightly at first and then gradually decreased through the rest of the study, with a mean ± SE of 42.66 ± 5.35 μg/L at baseline, 46.71 ± 6.54 μg/L at week 16, 35.12 ± 5·96 μg/L at week 48, and 25·50 ± 5·33 μg/L at week 144 (Fig. 1*D*). CTX, P1NP, and osteocalcin showed the same pattern of rapid increases followed by slow decrease to baseline levels, which were within the normal limits for the study population (Supplemental Table S1).

On baseline bone biopsy, 10 of 12 patients (83%) had elevated osteoid volume/bone volume and osteoid surface/bone surface compared with the upper limit of normal for 17.0‐ to 22.9‐year‐old healthy young adults.^(^ ^11^ ^)^ All patients in which mineralization lag time could be evaluated had increased baseline values compared with healthy young adults. However, only 6 of 12 patients (50%) had biopsies with abnormally high osteoid thickness, an important characteristic of osteomalacia on bone histomorphometry.

**Fig 2:**
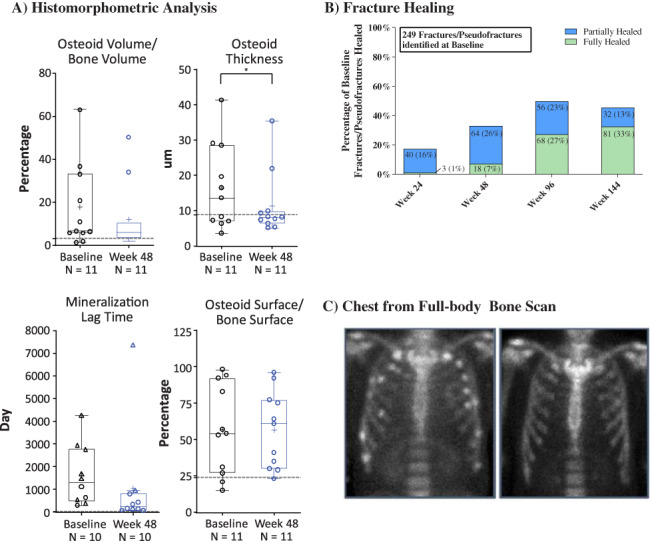
Histomorphometric and fracture assessments. Histomorphometric data (*A*) are presented as median, interquartile, 1.5× interquartile range, mean (+), and individual data points (○); for mineralization lag time, individual data points that were calculated using imputation are shown (∆);^(13)^ *p < .05. Gray line indicates upper limit of normal reference ranges for osteoid volume/bone volume 3.05%, osteoid thickness 8.9 μm, mineralization lag time 28.6 days, and osteoid surface/bone surface 23.9%, and are from Glorieux and colleagues.^(^ ^11^ ^)^ Fracture healing data (*B*) are presented as number and percentage of baseline fractures and pseudofractures. A total of 20 (8.03%) and 48 (19.3%) fractures and pseudofractures that were identified at baseline were considered not readable or were not evaluated at weeks 96 and 144, respectively. Zoomed‐in image of chest from a full‐body bone scan (*C*) demonstrates multiple fractures/pseudofractures at baseline with reduction in fractures/pseudofractures at week 144.

At week 48, a majority of osteomalacia‐related histomorphometric measures improved with burosumab. Osteoid volume/bone volume decreased from a mean ± SE of 17.6 ± 5.9% at baseline to 12.1 ± 4.7% at week 48, reflecting a mean absolute change ± SE of −5.5 ± 2.9% (95% CI −11.9 to 0.9; *p* = .0858) (Fig. 2*A*). Osteoid thickness changed from 16.5 ± 3.6 μm at baseline to 11.3 ± 2.8 μm at week 48, reflecting a mean ± SE change of −5.1 μm ± 2.2 μm (95% CI −10.0 to −0.2; *p* < .05). Mineralization lag time decreased from a mean ± SE of 1597.7 ± 419.5 days at baseline (imputation for 8 patients) to 1032.5 ± 711.6 days at week 48 (imputation for 2 patients), reflecting a mean absolute change ± SE of −565.2 ± 650.8 days (95% CI −2037.4 to 907.0; *p* = .4077). Osteoid surface/bone surface showed no change from baseline, with a mean ± SE of 57 ± 9% at baseline and 57 ± 7% at week 48 (absolute mean change −0.2% ± 6.1%, 95% CI −14% to 14%). Supplemental Table S2 provides the individual results. Improvements in bone biopsy parameters at week 48 were variable across individual patients with the largest improvements in patients with the most severe osteomalacia at baseline bone biopsy, as determined by osteoid thickness and mineralization lag time. Supplemental Table S3 reports additional histomorphometric parameters.

Using ^99m^Tc‐MDP whole‐body bone scans, 249 fractures and pseudofractures were detected at baseline in 14 patients with TIO. At week 96, 68 (27%) were fully healed and 56 (23%) were partially healed. At week 144, 81 (33%) were fully healed and 32 (13%) were partially healed (Fig. 2*B*). A total of 20 (8.03%) fractures and pseudofractures at week 96 and 48 (19.3%) fractures and pseudofractures at week 144 that were identified at baseline were considered not readable or were not evaluated because of patient discontinuation. The number of new fractures/pseudofractures was 19 at week 24, 17 at week 48, and 3 at weeks 96 and 144. Fig. 2*C* shows an example of fracture healing.

All three domains of the Brief Pain Inventory decreased with burosumab, indicating reductions in pain, with significant reduction in both pain severity and pain interference (Table 2). Similarly, patients reported a significant reduction in global fatigue, which includes fatigue severity and the impact of fatigue on daily functioning. Overall, patients showed an improvement in physical mobility. At baseline, the mean (SD) scores for the physical component domains of the SF‐36v2 were below the general population mean of 50 (10). By week 144, all scores had improved with significant increases in physical function, bodily pain, and the physical component summary. From baseline to week 144, physical function increased from 32.7 (9.8) to 41.7 (10.5; *p* = .0001); role physical increased from 33.6 (12.3) to 38.4 (14.6; *p* = .0545); bodily pain increased from 39.2 (10.5) to 43.3 (9.8; *p* = .0061); and the physical component summary increased from 32.8 (10.2) to 40.9 (11.8; *p* = .0011). For all measures, higher numbers indicated improved physical functioning. The number of repetitions in the sit‐to‐stand test also increased, with significant improvements found at week 24. The 6‐minute walk test was assessed in a fewer number of patients and only through week 48, demonstrating minimal change. The SF‐36v2 Mental Component Score was within or above the normal range (40 to 60) at baseline and throughout the study.

**Table 2:** Patient‐Reported Outcomes and Physical Functioning

Assessment	Mean (SD)	*n*
Brief Pain Inventory—worst pain score		
Baseline	5.0 (3.2)	14
Week 48	4.1 (3.1)	13
Week 144	4.1 (2.7)	10
Brief Pain Inventory—pain severity		
Baseline	3.9 (2.6)	14
Week 48	3.0 (2.6)	13
Week 144	3.1 (1.9)^**^	10
Brief Pain Inventory—pain interference		
Baseline	4.4 (3.2)	14
Week 48	3.4 (3.4)	13
Week 144	3.3 (2.8)^**^	10
Global fatigue score		
Baseline	5.6 (2.5)	13
Week 24	4.0 (2.6)^*^	14
Week 48	3.5 (3.0)^*^	13
Week 144	3.8 (2.2)^*^	10
SF‐36v2 physical component score		
Baseline	32.8 (10.2)	14
Week 24	36.9 (8.6)^*^	14
Week 48	39.3 (10.3)^**^	13
Week 144	40.9 (11.8)^*^	9
Sit‐to‐stand repetitions		
Baseline	6.7 (4.2)	10
Week 24	8.4 (4.3)^*^	10
Week 48	8.5 (4.2)^*^	10
6MWT, percentage predicted distance walked for age and sex		
Baseline	47.5 (30.8)	6
Week 48	51.8 (29.6)	6

6MWT = 6‐minute walk test.

Lower pain and fatigue scores indicate less pain and fatigue; lower SF‐36v2 scores indicate lower functioning.

^*^ *p* < .01 and ^**^ *p* < .05 based on least squares mean change from baseline using generalized estimating equation model.

### Safety

Safety is summarized for all patients with TIO who completed at least 144 weeks, and up to 192 weeks, with the exception of one patient with TIO who enrolled late and completed 124 weeks. One patient had an AE of neoplasm progression and discontinued burosumab to undergo chemotherapy (Table 3); this patient had lack of serum phosphorus response on burosumab but did not reach the maximum dose of 2 mg/kg. No other discontinuations occurred due to AEs.

**Table 3:** Safety Summary

Category	No. of events	Patients, *n* (%)
AE	360	14 (100.0)
Related AEs	16	9 (64.3)
Serious AEs	16	7 (50.0)
Related serious AEs	0	0
Grade 3 or 4 AEs	27	7 (50.0)
AEs leading to study discontinuation	0	0
AEs leading to treatment discontinuation	1	1 (7.1)
AEs leading to death	1	1 (7.1)
AEs of interest		
Injection site reactions	8	3 (21.4)
Hypersensitivity	4	2 (14.3)
Hyperphosphatemia	2	2 (14.3)
Ectopic mineralization	3	3 (21.4)
Restless leg syndrome	3	2 (14.3)
Most frequent AEs (occurring in ≥4 patients)		
Pain in extremity	—	9 (64.3)
Arthralgia	—	8 (57.1)
Upper respiratory tract infection	—	7 (50.0)
Cough	—	6 (42.9)
Neoplasm progression	—	5 (35.7)
Back pain	—	4 (28.6)
Diarrhea	—	4 (28.6)
Fall	—	4 (28.6)
Muscle spasms	—	4 (28.6)
Nasopharyngitis	—	4 (28.6)
Urinary tract infection	—	4 (28.6)

AE = adverse event.

Related serious AEs included injection site reaction, injection site pain, injection site swelling, hyperphosphatemia, vitamin D deficiency, tooth fracture, muscle spasms, dysgeusia, and rash.

All patients reported at least one AE, with the most frequently occurring AEs typical of TIO (eg, pain in an extremity) or common in the general population (eg, cough) (Table 3). Nine patients experienced 16 AEs considered related to burosumab by the investigator; all were mild in severity.

Predefined AEs of interest were injection site reactions, hypersensitivity, hyperphosphatemia, ectopic mineralization, and restless leg syndrome. There were 20 AEs of interest in eight patients; all events were mild to moderate in severity. Three patients had eight injection site reactions; all were considered related to burosumab. Two patients had four hypersensitivity events, all of which pertained to rash, were mild in severity, and resolved spontaneously or with topical medication; one event was considered related to burosumab. Two patients had two events of hyperphosphatemia; both were considered related to burosumab. One of these patients had a serum phosphorus level within the normal range at baseline (Supplemental Results) and experienced a TEAE of hyperphosphatemia on day 16. The second patient had undergone radiation therapy beginning at week 36. This patient experienced a TEAE of hyperphosphatemia on study day 478 (approximately week 68) and required a dose reduction. Three patients had three events of ectopic mineralization (two of these patients had a history of ectopic mineralization), and two patients had restless leg syndrome; all were considered unrelated to burosumab.

There were 16 serious AEs in seven patients; none were considered related to burosumab. Five patients had tumor progression/metastases; one patient had a tooth abscess, sialoadenitis, and rheumatoid arthritis; and one patient had tumor compression, Pickwickian syndrome, acute respiratory failure, cholangitis, pancreatitis, septic shock, and cardiac arrest resulting in death at week 51. Of the six patients with a serious AE related to tumor progression, five had a history of tumor progression before enrollment. Of these five patients with a history of tumor progression, two patients had new metastases identified when ^68^Ga‐DOTATATE PET/CT was first performed. All AEs related to tumor progression were considered unrelated to burosumab.

Throughout the study, there were no noteworthy changes in vital signs, echocardiogram and electrocardiogram parameters, or safety labs, including serum and urine calcium, serum parathyroid hormone, creatinine, hematology, and urinalysis (Supplemental Fig. S3). Mean (SD) estimated glomerular filtration rate (eGFR) decreased slightly but remained within the normal range. For the 10 patients with measurements at baseline and week 144, mean (SD) eGFR was 125.4 (30.1) at baseline and 112.6 (37.2) at week 144 (*p* = .0385). Renal ultrasound nephrocalcinosis score, ranging from 0 (normal) to 4 (stone formation),^(^ ^22^, ^23^ ^)^ was either 0 or 1 throughout the study for all patients. Only two patients tested positive for anti‐drug antibodies at week 120, while all other visits for these two patients and the remaining patients were negative; the presence of anti‐drug antibodies did not impact efficacy results. No neutralizing antibodies were detected at any time.

## Discussion

For the patients in this study, mean time since diagnosis was 14 years. During this time, most patients had a history of attempted surgical tumor resection, and all had received prior treatment with vitamin D analogues and/or oral phosphate. In spite of this, most entered the study with multiple fractures/pseudofractures, pain, fatigue, and impaired physical functioning. At baseline bone biopsy, most patients had increased osteoid volume/bone volume, osteoid surface/bone surface, and mineralization lag time, but only six patients had increased osteoid thickness compared with normal reference range. The variability in osteomalacia assessments via bone histomorphometry across the patient population may be the result of previous prolonged treatment with active vitamin D analogues and/or oral phosphate that resulted in partial healing of osteomalacia before study entry. Although osteoid volume/bone volume and osteoid surface/bone surface were assessed as a co‐primary endpoint in this study, it is important to note that both parameters can be elevated in conditions other than osteomalacia and should be interpreted in light of osteoid thickness and mineralization lag time.

Burosumab treatment in patients with TIO was associated with normalization of phosphate metabolism, improvement in histomorphometric measures of osteomalacia and BALP, enhanced fracture/pseudofracture healing, decreases in the number of new fractures, reductions in pain and fatigue, and improvements in physical functioning. The improvements in phosphate metabolism, pain, fatigue, and physical functioning occurred within 24 weeks and were maintained through 144 weeks of treatment. Benefits from burosumab treatment demonstrated in patients with TIO in this study were consistent with a case report of burosumab treatment in a patient with TIO, including normalization of serum phosphorus and improvement in pain and physical function,^(^ ^24^ ^)^ and in an interim analysis of a phase 2 trial of burosumab in Japanese and Korean patients with TIO.^(^ ^25^ ^)^ To our knowledge, these are the only other published studies of burosumab in TIO.

Burosumab demonstrated an acceptable safety profile, with most AEs mild to moderate in severity and generally reflecting the patients' underlying disease. Incidence of AEs of interest were assessed based on burosumab's known safety profile in X‐linked hypophosphatemia. AEs considered related to burosumab were injection site reactions (three patients, 21.4%), hypersensitivity (one patient, 7.1%), and hyperphosphatemia (two patients, 14.3%), similar to previous burosumab clinical trials.^(^ ^7^, ^9^, ^12^ ^)^ Six patients had an AE of tumor progression, all unrelated to burosumab. All but one of these patients had a history of tumor progression before enrollment, suggesting that changes in tumors during the study may represent the natural course of unresectable or incompletely resected phosphaturic mesenchymal tumors.^(^ ^2^, ^26^, ^27^ ^)^ Burosumab's mechanism of action targets excess FGF23, addressing renal phosphate wasting and impaired vitamin D synthesis with consequent osteomalacia. Although burosumab did not appear to impact the natural course of the underlying disease in this trial, longer‐term studies are needed to understand both the natural history of TIO and any potential effects of burosumab on tumor progression.

Patients had received prior phosphate treatment for a mean of 10.5 years and active vitamin D for a mean of 10.4 years. However, baseline bone biopsies and number of fractures/pseudofractures indicated poor skeletal health. Baseline mean osteoid volume/bone volume (17.6%) was similar to values observed in untreated patients with X‐linked hypophosphatemia (19.1% to 26.1%),^(^ ^12^, ^28^ ^)^ and was substantially above the range reported in healthy young adults (0.41% to 3.05%).^(^ ^11^ ^)^ Similarly, mean baseline osteoid thickness, mineralization lag time, and osteoid surface/bone surface were above the reference range, indicating poor skeletal health.^(^ ^11^, ^12^, ^29^, ^30^ ^)^

Alongside corrections in phosphate metabolism, burosumab substantially improved key histomorphometric measures of osteomalacia in a majority of patients. Concordant with improved osteomalacia, approximately half of the fractures/pseudofractures identified at baseline were fully or partially healed after 96 weeks and there was a reduction in new fractures/pseudofractures. These improvements are in line with results from burosumab trials in patients with X‐linked hypophosphatemia. As expected from improvements in phosphate metabolism, osteomalacia, and fracture healing, patients also reported beneficial changes in pain, fatigue, and physical functioning as early as week 24. Together, these findings suggest that by inhibiting excess FGF23 and restoring phosphate homeostasis, burosumab leads to meaningful improvements in overall bone health and health‐related quality of life in these patients.

Because of the rarity of TIO and invasive nature of bone biopsies, a control arm was not included, and baseline values served as a reference to evaluate changes. Still, most patients showed improvements in key histomorphometric parameters; it is also possible that measures of osteomalacia may have continued to improve with longer treatment, as was observed for changes in fracture healing at weeks 96 and 144. Additionally, there were consistent beneficial changes reported in hallmark symptoms of TIO, such as pain, fatigue, and physical functioning. Although this is the largest prospective study in TIO, it is limited by the relatively small sample size. The number of patients and resulting variability may have contributed to the nonsignificant improvements observed in some of the histomorphometric outcomes. Still, there was a significant improvement in osteoid thickness, which is a more reproducible assessment of osteomalacia. Additionally, all other histomorphometric parameters trended favorably, and there was variability in the severity of baseline osteomalacia.

In conclusion, data from this trial demonstrate that burosumab restores phosphate homeostasis and improves osteomalacia, fracture healing, and functional mobility, which translates into improvement in health‐related quality of life in patients with TIO. Overall, the results demonstrate a favorable benefit–risk profile of burosumab and offer a new therapeutic option for patients with TIO in whom surgical resection of the underlying tumor is not feasible.

## Disclosures

SMJdB received grants, personal fees, and non‐financial support from Ultragenyx and grants from Mereo. PDM received grants and participated in a scientific advisory board for Ultragenyx, Amgen, and Radius Health; received grants from Elxion; and is an employee of Colorado Center for Bone Research. TJW received grants and personal fees from Ultragenyx. MP received grants from Ultragenyx. KI received grants, personal fees, and other fees from Ultragenyx. RK has nothing to disclose. FR's lab performed analyses for Ultragenyx on a fee‐for‐service basis, and FR received personal fees from Mereo and Novartis and grants from PreciThera. DL and TC are employees and stockholders of Ultragenyx. MSR is an employee of Ultragenyx. JSM was an employee of Ultragenyx at the time the study was conducted, is an employee and shareholder of Arrowhead, and has a pending patent with Ultragenyx. TOC received grants and personal fees from Ultragenyx and personal fees from Kyowa Kirin and Inozyme. This study was sponsored and funded by Ultragenyx Pharmaceutical Inc. in partnership with Kyowa Kirin International plc. Medical writing support was provided by Catherine Woods (Ultragenyx) and Kerri Hebard‐Massey (Ultragenyx).

## Author Contributions

SMJdB: Conceptualization; investigation; writing‐original draft; writing‐review and editing. PDM: Investigation; writing‐review and editing. TJW: Investigation; writing‐review and editing. MP: Investigation; writing‐review and editing. KI: Investigation; writing‐review and editing. RK: Investigation; writing‐review and editing. FR: Investigation; writing‐review and editing. DL: Conceptualization; formal analysis; investigation; writing‐review and editing. TC: Conceptualization; formal analysis; investigation; writing‐review and editing. MSR: Conceptualization; formal analysis; writing‐original draft; writing‐review and editing. JSM: Conceptualization; writing‐review and editing. TC: Conceptualization; investigation; writing‐original draft; writing‐review and editing.

### Peer Review

The peer review history for this article is available at https://publons.com/publon/10.1002/jbmr.4233.

## Supporting information


**Supplemental Fig. S1.** CONSORT diagram.
**Supplemental Fig. S2.** Pharmacodynamic assessments (mass units).
**Supplemental Fig. S3.** Key safety labs (mass units).
**Supplemental Table S1.** Bone Turnover Markers
**Supplemental Table S2.** Individual Bone Biopsy Assessments
**Supplemental Table S3.** Additional Histomorphometric Parameters
**Supplemental Results.** One patient was enrolled in this study based on inclusionary serum phosphorus levels per testing performed at a local laboratory. However, their serum phosphorus levels were within the normal range per central laboratory testing (data on file), which likely triggered the TEAE of hyperphosphatemia on study day 16. The patient received minimal burosumab dosing during the study (weeks 0, 8, 32, and 74, with doses ranging from 0.15 to 0.3 mg/kg) as the patient's serum phosphorus levels did not warrant dosing per protocol‐specified guidelines. Concentrations of burosumab in this subject were significantly lower than in patients who received regular dosing. This patient was discontinued due to minimal burosumab dosing.

**Appendix** **S1**: Supporting Information
