# Supplementary material for: Burosumab for the Treatment of Tumor‐Induced Osteomalacia
Source: J Bone Miner Res. 2021 Jan 12;36(4):627–35. doi: 10.1002/jbmr.4233 (PMC8247961; doi:10.1002/jbmr.4233)
Supplement: Supplementary file 1 — Supplemental Fig. S1. CONSORT diagram. Supplemental Fig. S2. Pharmacodynamic assessments (mass units). Supplemental Fig. S3. Key safety labs (mass units). Supplemental Table S1. Bone Turnover Markers Supplemental Table S2. Individual Bone Biopsy Assessments Supplemental Table S3. Additional Histomorphometric Parameters Supplemental Results. One patient was enrolled in this study based on inclusionary serum phosphorus levels per testing performed at a local laboratory. However, their serum phosphorus levels were within the normal range per central laboratory testing (data on file), which likely triggered the TEAE of hyperphosphatemia on study day 16. The patient received minimal burosumab dosing during the study (weeks 0, 8, 32, and 74, with doses ranging from 0.15 to 0.3 mg/kg) as the patient's serum phosphorus levels did not warrant dosing per protocol‐specified guidelines. Concentrations of burosumab in this subject were significantly lower than in patients who received regular dosing. This patient was discontinued due to minimal burosumab dosing. [file JBMR-36-627-s002.docx]

Supplemental **Results:**

One patient was enrolled in this study based on inclusionary serum phosphorus levels per testing performed at a local laboratory. However, their serum phosphorus levels were within the normal range per central laboratory testing (data on file), which likely triggered the TEAE of hyperphosphatemia on Study Day 16. The patient received minimal burosumab dosing during the study (Weeks 0, 8, 32, and 74, with doses ranging from 0.15 to 0.3 mg/kg) as the patient’s serum phosphorus levels did not warrant dosing per protocol-specified guidelines. Concentrations of burosumab in this subject were significantly lower than in patients who received regular dosing. This patient was discontinued due to minimal burosumab dosing.

## Supplemental Figure 1. CONSORT Diagram

**
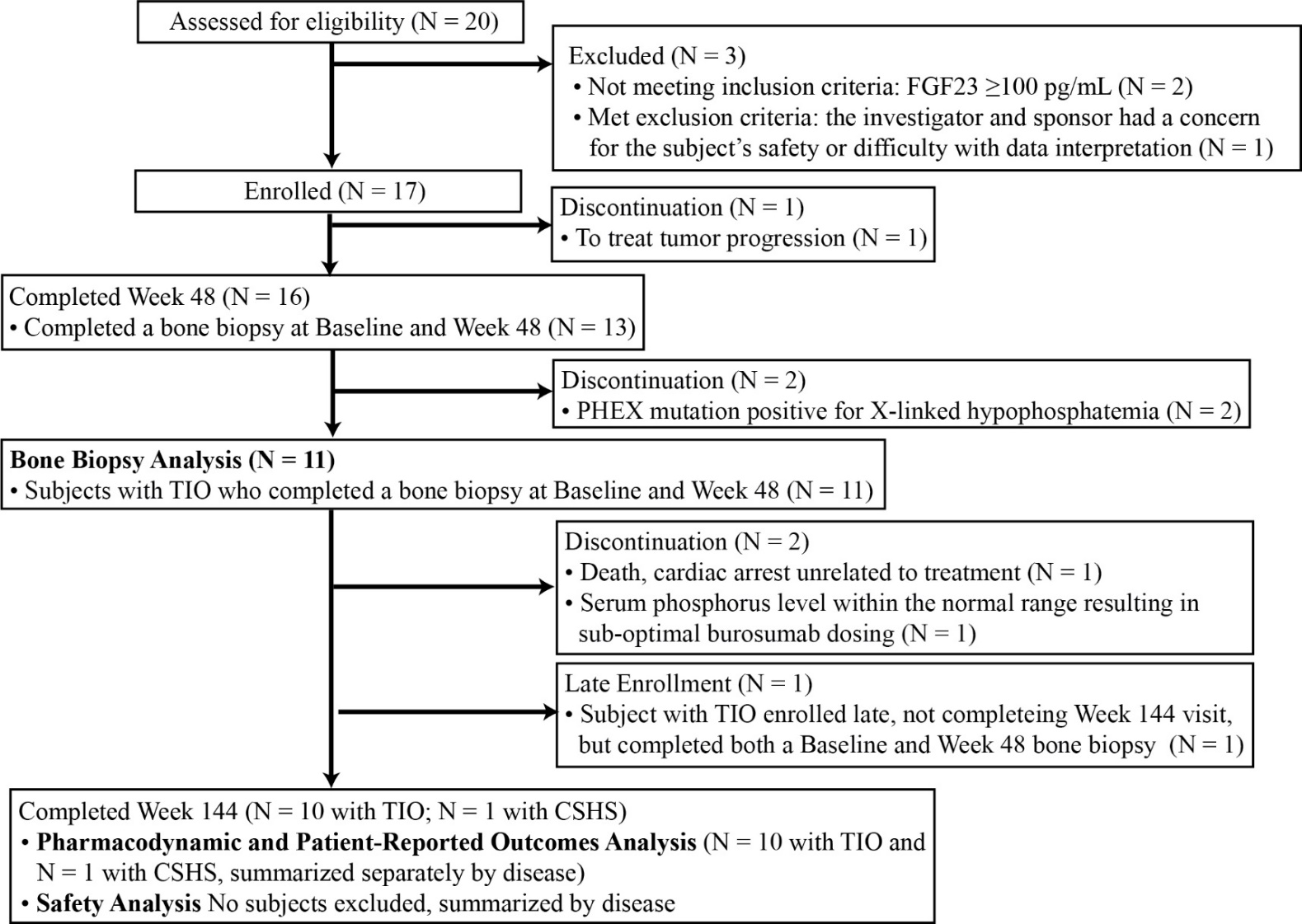
**

Data from the 14 patients with TIO are included in this manuscript. At baseline, 14/16 patients had bone biopsies. By Week 48, 11 of those 14 patients remained because of discontinuation and exclusion of 2 patients with XLH, resulting in 11 patients with bone biopsies at Week 48.

## Supplemental Figure 2. Pharmacodynamic Assessments (Mass Units)

Data are presented as mean ± standard deviation. Baseline data points are shown in black, end-point of the dose interval data points are shown in green, and mid-point of the dose interval data points are shown in blue, with the exception of Week 21 for 1,25(OH)_2_D shown in blue which is neither the mid- or end-point of the dose cycle. TmP/GFR, renal tubular reabsorption of phosphate.

## Supplemental Figure 3. Key Safety Labs (mass units)

Data are presented as mean ± standard deviation. All post-baseline data points were measured at the end of dose cycle.

## Supplemental Table 1. Bone Turnover Markers

|  | Baseline N=14 | Week 24 N=14 | Week 48 N=13 | Week 96 N=12 | Week 144 N=10 |
| --- | --- | --- | --- | --- | --- |
| CTx, pg/mL, mean (SE) | 582.1 (87.3) | 801.4 (173.3) | 693.1 (104.4) | 592.5 (82.2) | 561.0 (67.8) |
| P1NP, ng/mL, mean (SE) | 71.7 (7.7) | 105.1 (13.2) | 80.1 (9.7) | 81.1 (17.4) | 71.6 (5.9) |
| Osteocalcin, ng/mL, mean (SE) | 25.8 (6.1) | 35.5 (8.4) | 29.0 (7.4)* | 25.5 (4.2) | 26.0 (5.5)* |

## *N=11 at Week 48 and 9 at Week 144. CTx: carboxy terminal cross-linked telopeptide of type I collagen; P1NP: procollagen type 1 N-propeptide.

Normal ranges for CTX are 120-830 pg/mL for men, 50-670 pg/mL for pre-menopausal women, and 90-1005 pg/mL for postmenopausal women (Michelson 2013). Normal ranges for P1NP are 31-96 ng/mL for men, 19-76 ng/mL for pre-menopausal women, and 18-102 ng/mL for postmenopausal women. Normal ranges for osteocalcin are 9-38 ng/mL for men and 8-32 ng/mL for women.

## Supplemental Table 2. Individual Bone Biopsy Assessments

| **Patient** | **Diagnosis** | **Osteoid Volume/ Bone Volume, %** | | **Osteoid Surface/ Bone Surface, %** | | **Osteoid Thickness, µm** | | **Mineralization Lag Time, days** | |
| --- | --- | --- | --- | --- | --- | --- | --- | --- | --- |
|  |  | Baseline | Week 48 | Baseline | Week 48 | Baseline | Week 48 | Baseline | Week 48 |
| 1 | CSHS | NE | NE | NE | 16 | NE | 8.7 | NE | 12.2 |
| 2 | TIO | NE | 5.6 | NE | 52 | NE | 8.9 | NE | 63.2 |
| 3 | TIO | NE | 8.9 | NE | 54 | NE | 7.1 | NE | 1183.4 |
| 4 | TIO | 1.3^b^ | 4.6 | 15 | 41 | 7 | 9.3 | 518.5 | 52.2 |
| 5 | TIO | 1.9^b^ | 4.1 | 27 | 30 | 3.5 | 6.4 | 376.3 | 90.3 |
| 6 | TIO | 5.9 | 5.9 | 57 | 61 | 8.2 | 7.6 | 1102 | 147.7 |
| 7 | TIO | 5.9 | 1.8 | 21 | 29 | 19.6 | 5.4 | 277.3 | 59.2 |
| 8 | TIO | 6.5 | 3.1 | 31 | 23 | 6.4 | 5.1 | 4266.7 | 944.4 |
| 9 | TIO | 6.7 | 8.3 | 54 | 77 | 7.3 | 8.2 | 622.6 | 785.4 |
| 10 | TIO | 6.8 | NE | 56 | NE | 5.5 | NE | 1833.3 | NE |
| 11 | TIO | 11 | 7.8 | 53 | 64 | 13.6 | 8.1 | 1462.4 | 122.3 |
| 12 | TIO | 21 | 10.3 | 94 | 75 | 16.5 | 9.9 | 2750 | 329.2 |
| 13 | TIO | 33.2 | 3.5 | 83 | 35 | 28.5 | 7.4 | NE | 17.5 |
| 14 | TIO | 36.9 | 33.9 | 92 | 92 | 29.1 | 21.9 | 1672.4 | 419.6 |
| 15 | TIO | 63.4 | 50.2 | 98 | 96 | 41.3 | 35.4 | 2929.1 | 7375 |

^a^Using imputation; Upper limit of normal reference ranges for osteoid volume/bone volume 3.05%, osteoid thickness 8.9 µm, osteoid surface/bone surface 23.9%, and mineralization lag time 28.6 days and are from Glorieux et al. 2000;

^b^Regarding the two patients with no baseline osteomalacia, bone formation activity was extremely slow as indicated by low Baseline MS/BS of 0.7% and 0.9%, respectively, which may explain why osteoid indices were within the normal range despite presence of hypophosphatemia. The increase in amount of osteoid at Week 48 likely reflects an increase in bone cell activity as indicated by an increase in MS/BS (12.4% and 3.2%, respectively) rather than a worsening of the mineralization defect.

NE, not evaluable

## Supplemental Table 3. Additional Histomorphometric Parameters

| **Parameter** | **Current Study**  **UX023T-CL201** | |  | **Healthy Adult Reference Range^a^** |
| --- | --- | --- | --- | --- |
|  | **N** | **Mean (SD)**  **Median (min, max)** |  | **Mean (SD)**  **Median (min, max)** |
| **Bone volume/tissue volume**, % |  |  |  |  |
| Baseline | 10 | 23.4 (11.5)  22.4 (5.5, 41.8) |  | 27.8 (4.5)  27.4 (18.9, 34.7) |
| Week 48 | 10 | 23.6 (9.8)  23.4 (7.7, 40.6) |  |  |
| **Cortical width**, µm |  |  |  |  |
| Baseline | 9 | 1242 (763)  1027 (455, 2854) |  | 1010 (200)  1080 (630, 1300) |
| Week 48 | 10 | 1188 (722)  980 (480, 2835) |  |  |
| **Trabecular thickness**, µm |  |  |  |  |
| Baseline | 11 | 136 (32)  146 (60, 169) |  | 153 (24)  156 (111, 192) |
| Week 48 | 11 | 139 (31)  146 (77, 176) |  |  |
| **Mineralizing surface/bone surface, %** |  |  |  |  |
| Baseline | 10 | 2.1 (1.7)  1.8 (0.1, 5.4) |  | 7.9 (2.7)  8.3 (3.3, 12.4) |
| Week 48 | 10 | 6.4 (5.2)  5.1 (1.9, 9.7) |  |  |
| **Mineralizing surface/osteoid surface, %** |  |  |  |  |
| Baseline | 10 | 4.3 (3.9)  3.2 (0.5, 14.4) |  | 57.9 (13.8)  59.2 (39.9, 74.9) |
| Week 48 | 11 | 14.1 (14.7)  10.6 (1.6, 50.3) |  |  |
| **Mineral apposition rate, µm/d** |  |  |  |  |
| Baseline | 10^b^ | 0.34 (0.11)  0.30 (0.2, 0.6) |  | 0.75 (0.09)  0.77 (0.57, 0.86) |
| Week 48 | 11^b^ | 0.52 (0.16)  0.51 (0.3, 0.8) |  |  |
| **Osteoclast surface/bone surface, %** |  |  |  |  |
| Baseline | 9 | 0.3 (0.4)  0.1 (0.1, 1.3) |  | 1.0 (0.4)  0.9 (0.5, 1.9) |
| Week 48 | 9 | 0.3 (0.3)  0.2 (0.1, 1.0) |  |  |
| **Number of Osteoclasts/bone perimeter, /mm** |  |  |  |  |
| Baseline | 9 | 0.1 (0.1)  0.1 (0.0, 0.4) |  | 0.3 (0.1)  0.3 (0.2, 0.6) |
| Week 48 | 9 | 0.1 (0.1)  0.1 (0.0, 0.3) |  |  |

^a^Healthy reference range is based on data from 8-12 individuals between the ages of 17.0 and 22.9 years who underwent surgery for reasons independent of abnormalities in bone development and metabolism (Glorieux FH, Travers R, Taylor A, et al. Normative data for iliac bone histomorphometry in growing children. Bone 2000;26:103-9).

^b^Mineralization lag time was calculated using an established imputation technique (7 patients at baseline and 2 patients at Week 48) (Dempster DW, Compston JE, Drezner MK, et al. Standardized nomenclature, symbols, and units for bone histomorphometry: a 2012 update of the report of the ASBMR Histomorphometry Nomenclature Committee. J Bone Miner Res 2013;28:2-17. 10.1002/jbmr.1805).
