# Supplementary material for: Burosumab for the Treatment of Tumor‐Induced Osteomalacia
Source: J Bone Miner Res. 2021 Jan 12;36(4):627–35. doi: 10.1002/jbmr.4233 (PMC8247961; doi:10.1002/jbmr.4233)
Supplement: Supplementary file 2 — Appendix S1: Supporting Information [file JBMR-36-627-s001.pdf]

A phase 2 open-label trial to assess the efficacy and safety of KRN23, an antibody to FGF23, in subjects with tumor-induced osteomalacia (TIO) or epidermal nevus syndrome (ENS)-associated osteomalacia

**Protocol Number:**	**UX023T-CL201**
**Original Protocol:**	**29 August 2014**
**Amendment 1:**	**21 January 2015**
**Amendment 2:**	**14 October 2015**
**Amendment 3:**	**17 February 2016**
**Amendment 4:**	**10 November 2017**
**Amendment 5:**	**27 April 2018**
**Amendment 6:**	**26 September 2019**

**Investigational Product:**	KRN23 (recombinant human IgG_1 monoclonal antibody (mAb) to fibroblast growth factor 23 [FGF23])
**Indication:**	Treatment of tumor-induced osteomalacia (TIO) and epidermal nevus syndrome (ENS)-associated osteomalacia
**IND / EudraCT Number:**	123878 / Not Applicable
**Sponsor:**	Ultragenyx Pharmaceutical Inc.<br>60 Leveroni Court<br>Novato, CA USA 94949
**Sponsor's Responsible Medical Officer:**	Javier San Martin, MD<br>Senior Vice President, Global Clinical Development<br>Ultragenyx Pharmaceutical Inc.<br>60 Leveroni Court<br>Novato, CA USA 94949<br>PPD
**Coordinating Investigator:**	Suzanne Jan de Beur, MD<br>Johns Hopkins University School of Medicine<br>Baltimore, MD, 21224, USA

This study is to be performed in compliance with the protocol, Good Clinical Practice (GCP), and applicable regulatory requirements.

Confidentiality Statement: This document and the information it contains is confidential and proprietary and is the property of Ultragenyx Pharmaceutical Inc. (Ultragenyx). Unauthorized disclosure, reproduction, transmission, or use of this document or information is strictly prohibited. By accepting this document, the recipient agrees that the information it contains will not be disclosed without written authorization from Ultragenyx, except to the extent required to satisfy Institutional Review Board procedures /Ethics Committee, to obtain written informed consent from potential subjects, or as required by law.

CLINICAL STUDY PROTOCOL AMENDMENT

SUMMARY OF CHANGES AND RATIONALE

UX023T-CL201 Amendment 1

21 January 2015

Protocol UX023T-CL201 (dated 29 August 2014) has been modified by Amendment 1 to incorporate feedback provided by the US Food and Drug Administration (FDA) and investigational study sites, and to clarify details/schedule of procedures to be conducted in the study. The protocol changes are summarized below:

1. **Study Sites:** The number of study sites has been increased from a single site to multiple sites.

   Rationale: Due to the rare nature of TIO/ENS, additional sites may be necessary in order to identify enough subjects to fully enroll the study.

2. **Schedule of Events:** Updates have been made to the frequency or timing of certain assessments as detailed in Table 2.1 and Table 2.2 (Schedule of Events). In addition, the first telephone call was changed to a home health visit at Study Week 1 and a new home health visit (HHV10.1) was added at Study Week 21.

   Rationale: Changes have been made to reduce the number of measurements for reasons of subject convenience or to clarify differences in specific assessments taken at clinic visits versus home health visits. The sponsor believes the changes in assessment frequency and timing will not influence the ability to evaluate the safety and efficacy of study drug. Home health visits 1 and 10.1 will ensure that phosphorus, 1,25$(OH)_2$D, and calcium will be adequately collected and measured throughout the study.

3. **Inclusion Criteria:** Inclusion Criterion #6 (Section 7.3.1) has been modified to show that subjects with an estimated glomerular filtration rate (eGFR) of ≥30 but <60 mL/min will be considered eligible so long as, in the opinion of the investigator, the decline in renal function is not related to nephrocalcinosis.

   Rationale: Older adult subjects may routinely have eGFR <60 mL/min without there being any indication of nephrocalcinosis.

4. **Exclusion Criteria** (Section 7.3.2)**:**

   a. Exclusion Criterion #11 has been modified to show that a history of allergic reaction to or have shown adverse reactions to declomycin is no longer exclusionary.

Rationale: Labeling with declomycin is no longer required. Labelling subjects with 2 rounds of tetracycline HCL prior to each bone biopsy is sufficient to allow the biopsy to be read appropriately.

b. Exclusion Criterion #12 has been modified to clarify that the decision to exclude subjects who have conditions which could present a concern for either subject safety or difficulty with data interpretation can be made by the investigator and the sponsor.

Rationale: Many patients with ENS have a number of potentially confounding comorbidities. To help ensure subject safety and the integrity of the data collected in the study, both the investigator and the sponsor may decide to exclude subjects based on these potentially confounding comorbidities.

5. **Bone Biopsy Labeling:** Section 7.5 has been modified to show that subjects will be labelled with 2 rounds of tetracycline HCL prior to each bone biopsy rather than 1 round of declomycine (or equivalent) followed by 1 round of tetracycline.

Rationale: Labeling with declomycin is no longer required. Labelling subjects with 2 rounds of tetracycline HCL prior to each bone biopsy is sufficient to allow the biopsy to be read appropriately.

6. **Bone Biopsy:** Table 2.1 (Schedule of events) has been revised to note that a bone biopsy is not required at the Baseline visit if a previous bone biopsy confirmed the diagnosis of osteomalacia, the subjects' clinical manifestations have not changed significantly since the time of the previous biopsy/diagnosis, and the tissue collected at that biopsy is made available for testing for this protocol. A similar change has been made to Section 7.5.

Rationale: It is expected that there would be no significant change in untreated TIO/ENS subjects from the time that their original bone biopsy confirmed the diagnosis of osteomalacia; therefore a historical sample can be used as the baseline comparator within this study.

7. **Clinical Outcome Measures:** Section 7.5.3.2 has been revised to replace the 6MWT with the Sit to Stand (STS) test and the Weighted Arm Lift (WAL) test.

Rationale: The 6MWT has been removed because some subjects may not be ambulatory and thus not able to complete the test.

8. **Tumor Imaging:** Imaging of the tumor (Section 7.5.5.9) has been incorporated at the request of the FDA. Imaging will be completed at the screening visit, and every 6 months thereafter for tumors that are visible at screening.

Rationale: As part of the overall safety assessment of TIO/ENS patients, evaluation of overall change in tumor size and volume is being incorporated into this study.

9. **Fasting Serum Phosphate Range:** The synopsis and Sections 7.1, 7.4, and 7.5 were updated to change the target fasting serum phosphate range from 2.5 – 3.5 mg/dL to 2.5 – 4.0 mg/dL.

   Rationale: The fasting serum phosphate range is being expanded to allow for additional flexibility.

10. **Bone Scan Assessment of Bone Turnover:** Criteria for evaluation were updated; the bone scan will not be utilized to assess bone turnover and the number of central readers will be reduced from three readers to a single reader.

    Rationale: Due to high sensitivity but low specificity and the undefined temporal appearance of pseudofracture healing by bone scan, bone scan will be used to identify potential pseudofractures at BL, wk 24 and wk 48. For this purpose, a single central reader is deemed appropriate. Exploratory analyses of change from BL will be defined in the SAP.

CLINICAL STUDY PROTOCOL AMENDMENT

SUMMARY OF CHANGES AND RATIONALE

UX023T-CL201 Amendment 2

14 October 2015

Protocol UX023T-CL201 Amendment 1 (dated 21 January 2015) has been modified by Amendment 2 to increase the sample size, modify the description of ENS, clarify details/schedule of procedures to be conducted in the study, and to make other minor modifications. Important protocol changes are summarized below:

1. **Sample Size:** In Section 7.1, the sample size has been increased from approximately 6 to approximately 15 adult subjects.

   Rationale: The Sponsor has become aware of additional subjects meeting eligibility criteria.

2. **Description of ENS:** The description of ENS has been updated throughout the protocol to indicate that ENS is not merely a variant of TIO but a distinct congenital disorder of which one syndrome includes FGF23-induced hypophosphatemia and associated osteomalacia and shares a similar biochemical and clinical profile to TIO.

   Rationale: Based on a recent literature review on ENS, it is apparent that unlike the mesenchymal tumors that typically characterize TIO, epidermal nevi are non-neoplastic hematoma. Further, FGF23 protein or FGF23 mRNA is not detectable in samples of ENS-associated osteomalacia. Both TIO and ENS share the underlying pathophysiology of elevated serum FGF23 which leads to hypophosphatemia and the associated metabolic bone disease. Unlike TIO, complete resection and surgical cure leading to removal of the source of excess FGF23 is less likely. Nonetheless, in older literature case reports, there has been documentation of improved phosphorus levels following skin debridement/excision surgery in some but not all cases. ENS lesions may be extensive, often covering 10-60% of the skin. Thus the protocol was updated with this new information.

3. **Primary Efficacy Evaluation**: Section 7.5 and Section 7.6 have been updated to specify co-primary efficacy evaluations. Specifically, efficacy will be evaluated based on change from baseline in serum phosphorus levels over time and change from baseline in osteomalacia at 48 weeks as determined by histomorphometric evaluation of trans-iliac crest bone biopsies.

Rationale: TIO and ENS-associated osteomalacia are clinically heterogeneous; some patients experience substantial bone pain, and muscle weakness is common, but the frequency and intensity of these symptoms vary significant from patient to patient. However, all subjects with these disorders have elevated FGF23 levels and consequent hypophosphatemia and osteomalacia. Moreover, when surgical treatment of TIO and ENS-associated osteomalacia is successful, serum phosphorus levels normalize and osteomalacia resolves. Thus, improvement in serum phosphorus levels and improvement in osteomalacia as determined by bone biopsy assessment have been selected as the primary endpoints for this study and are meaningful endpoints to evaluate the efficacy of KRN23 treatment in this population of subjects whose tumor or skin lesions are considered inoperable.

4. **<u>Inclusion Criteria</u>**: Inclusion criterion number 4 has been updated to indicate an iFGF23 level ≥100 pg/mL as measured by Kainos assay is required for inclusion in the study. Previously the required FGF23 level was ≥2 times the upper limit of normal.

   Rationale: A review of the FGF23 assay literature suggested that a normal level was not well established for TIO and ENS-associated osteomalacia. A study of FGF23 levels in patients with TIO or ENS found that 12 of 13 patients studied had FGF23 levels at or above 100 pg/mL (Ito et al. 2005), thus the Sponsor has selected this level as a reasonable cutoff to ensure eligible patients do have elevated FGF23 consistent with TIO or ENS-associated osteomalacia.

5. **<u>Previous Bone Biopsy</u>**: Section 7.5.2 has been updated with additional details about the acceptability of using results from a previous trans-iliac crest bone biopsy as the Baseline bone biopsy assessment. A previous bone biopsy will be acceptable if it was obtained within 12 months of Screening, confirmed the diagnosis of osteomalacia, the subjects' clinical manifestations have not changed significantly since the time of the previous biopsy/diagnosis, and the tissue collected at that biopsy is made available for testing for this protocol. Evaluability of previous bone biopsy specimens must be confirmed by a central reader before Baseline. Dosing may begin once evaluability is confirmed.

   Rationale: Given that determining the effects of KRN23 on improvement in osteomalacia in trans-iliac crest bone biopsies is now a co-primary objective in the study, it is essential that a previously obtained bone biopsy is reflective of a subject's baseline disease and is evaluable for determination of the endpoint. Thus, it will now be required that the biopsy be taken within 12 months of Screening and that its evaluability is confirmed prior to baseline and dosing.

6. **<u>Study Procedures and Assessments</u>:** Section 7.5 has been amended to add or remove certain assessments.

   Functional Outcomes and PROs: The Six Minute Walk Test (6MWT) has been added and will be administered at Screening (for practice), Baseline (Day -2), and Weeks 12,

24, and 48 (or Early Termination). The Timed Up and Go (TUG) and WOMAC assessments have been removed.

Rationale: Testing of initial study subjects has provided insights into the functional abilities and limitations of subjects with TIO and ENS-associated osteomalacia leading to these changes. Screening data from the initial TUG administrations suggest that the test does not appear to be sensitive to the functional limitations experienced by patients with TIO or ENS-associated osteomalacia as all of the subjects were able to complete the test without difficulty. The TUG has been replaced with the 6MWT, which is longer in duration and requires more energy expenditure for completion. These features increase the likelihood that the 6MWT will capture the impact of the mobility deficits, bone/joint pain, and muscle weakness experienced by patients with TIO or ENS-associated osteomalacia. In addition to the TUG, the WOMAC has been removed due to issues identified during the initial administrations of the PRO measure. A standardized administration of the WOMAC, which was originally developed for use in osteoarthritis, requires the subject to complete all items based on the impact to the hips and/or knees or a single joint. Subjects have reported difficulty with the identification of a target area given the impact of TIO on various bones and joints throughout the body, including the neck, back and upper and lower extremities. Subjects have reported difficulty with the identification of a single joint for assessment given the impact of TIO on various bones and joints throughout the body. The remaining PROs will continue to be administered in the protocol.

Tumor Imaging: In Section 7.5.6.9, changes have been made to clarify the methodology related to tumor imaging. Tumor imaging will be done for TIO subjects only and not subjects with ENS-associated osteomalacia. (Assessment of skin lesions in ENS subjects is addressed below.) CT and MRI imaging will be performed with the capability of assessing tumor size if the tumor is localized. The radiologist will indicate whether tumor size and volume have changed over time. If applicable, RECIST measurements will be done.

Rationale: Many TIO tumors are difficult to localize because they are very small, slow growing, and often are in areas of bone or soft tissue which make them difficult to find using recommended imaging tools. Common localization techniques involve the use of high resolution whole body CT or MRI often in combination with FDG PET scans and/or octreotide scans, which are both highly sensitive but not specific. A proportion of the patients who will be enrolled in the UX023T-CL201 may not have had their tumor localized. In instances where these tumors were previously localized, these patients may not have responded to surgical excision. Images from CT/MRI assessments will be analyzed for assessment by independent radiologist readers using software that is compatible with RECIST. If a lesion is present it is measured and marked with an arrow. The radiologist will review and indicate whether tumor size and volume have changed over 6 monthly intervals. Given the knowledge of the slow growing natural history of these mesenchymal tumors, it is expected that little change in tumor size will be detected. RECIST scores will be assessed if applicable.

Dermatologic Assessment for Subjects with ENS-associated Osteomalacia: Section 7.5.6.10 has been updated to add that serial photographs of skin lesions will be taken at Baseline and post treatment at Weeks 12, 24, 36, and 48 to assess progress in the skin lesions over time with KRN23 treatment (ENS subjects only).

Rationale: Serial photographs for dermatologic assessment has been added to formally capture progress in skin lesions over time as a safety assessment.

7. **Safety Contact Information:** The safety contact information in Section 8.5.5 has been updated to reflect the most current information.

   Rationale: Administrative change.

CLINICAL STUDY PROTOCOL AMENDMENT

SUMMARY OF CHANGES AND RATIONALE

UX023T-CL201 Amendment 3

17 February 2016

Protocol UX023T-CL201 Amendment 2 (dated 14 October 2015) has been modified by Amendment 3 to extend the study duration by 96 weeks, to clarify assessments in the screening skeletal survey; to add routine lipase testing and reflexive testing for serum amylase isoenzymes if serum amylase levels are elevated; to update a follow-up period for adverse events and duration of effective contraception after stopping study drug; to update an inclusion and exclusion criteria; to specify language for tetracycline labeling; to update that echocardiogram will be read centrally; and to update disease information for TIO/ENS and summary of overall risks and potential benefits of KRN23 to be consistent with the Investigator's Brochure. Important protocol changes are summarized below:

1. **Overall Study Design and Plan**: Section 7.1 has been updated to extend the study duration. After completing the initial treatment period of the study (48 weeks), subjects may continue into a planned Treatment Extension Period in which subjects will receive KRN23 treatment for up to an additional 96 weeks or until one of the following occurs: the study drug is commercially available in the subject's local territory, the subject withdraws consent and discontinues from the study, the subject is discontinued from the study at the discretion of the Investigator or Ultragenyx, or the study is terminated.

   Rationale: The goal of the 96-week Treatment Extension Period is to evaluate the long-term safety and efficacy of KRN23 in patients with TIO/ENS. In eligible patients where tumors are considered inoperable due to the location of the tumor or extent of disease or an inability to localize the tumor, hypophosphatemia is a chronic disease. It is expected that the maintenance of phosphate control will allow for continued healing of osteomalacia in patients with TIO/ENS. The 96-week Treatment Extension Period will provide long-term data on safety and efficacy to further characterize the benefit:risk profile of KRN23 treatment in this patient population.

2. **Clinical Assessments**: Section 7.5.4.3 has been updated to clarify assessments in the screening skeletal survey.

   Rationale: The protocol has been amended to provide clarification and alignment with central imaging requirements with regards to the skeletal survey of the lateral spine, AP chest, right and left hand/wrist, right and left humerus, right and left radius/ulna, right and

left femur/pelvis, right and left tibia/fibula, and right and left foot to be obtained at screening.

3. **Safety Measurements**: Section 7.5.6.4 has been updated to add assessments of serum lipase in all subjects and to specify additional laboratory analyses that will be performed reflexively on prospectively drawn specimens if serum amylase levels are elevated to ≥2x the upper limit of the reference range (ULRR).

   Rationale: Mild asymptomatic elevations in serum amylase have been observed in some subjects at baseline and during the study. Amylase is produced by several organs including the pancreas and salivary gland and so elevated amylase levels are not diagnostic in the absence of other information. In ongoing and completed KRN23 studies at baseline, mild elevations of amylase (<2x ULRR) have been noted. Post treatment mild shifts in amylase elevation (< 2xULRR) has been noted without association with GI symptoms. No adverse events of pancreatitis have been observed. The reflexive testing for serum amylase isoenzymes when serum amylase levels are elevated will allow a determination of whether the elevations are from pancreatic or salivary gland sources.

4. **Pregnancy Testing and Reporting and Follow-up of Adverse Events:** In Section 7.5.6.13 and Section 8.5, the follow-up period for adverse events (AEs) and duration of contraception after the end of the study have been extended from 30 days to 12 weeks.

   Rationale: Subjects will need to be followed for approximately 5 times the elimination half-life. The half-life is 16.4 days making the duration of follow up approximately 3 months. Thus, participants of child-bearing potential or with partners of child-bearing potential who have not undergone a bilateral salpingo-oophorectomy and are sexually active must consent to use two forms of effective method of contraception as determined by the principal investigator from the period following the signing of informed consent through 12 weeks (approximately 5 times the elimination half-life) after stopping the study drug. Language in the inclusion criteria has also been amended to reflect this change (see below).

   Additionally, all AEs will be collected from the time the subject signs informed consent through 12 weeks (approximately 5 times the elimination half-life) following the last dose of study drug.

4. **Inclusion and Exclusion Criteria**: An inclusion criterion in Section 7.3.1 and an exclusion criterion in Section 7.3.2 have been updated.

   Rationale: Inclusion criterion #9 was updated to clarify that "*subjects must be willing to use two forms of effective methods of contraception while participating in the study (sexually active subjects) and for 12 weeks after the last dose of study drug*." This change is intended to align with the revised Pregnancy Testing and Reporting language in Section 7.5.6.13 as described above. In addition, exclusion criterion #9 was updated to "*Have a history of malignancy within 5 years of study entry with the exception of PMT-MCT (Phosphaturic mesenchymal tumors of the mixed connective tissue type)*

tumors or non-melanoma skin cancers such as basal cell skin cancer." This criterion was amended to ensure that the safety profile of KRN23 can be properly characterized in patients with classic TIO (PMT-MCT) before being studied in subjects with TIO-like syndrome, such as prostate cancer, lung cancer, etc.

5. **Study Procedures and Assessments**: In Section 7.5 and throughout the document, the language for use of a tetracycline has been updated to provide clarity.

   Rationale: The current language in the protocol was narrowly worded to allow use of tetracycline hydrochloride (HCl) to facilitate histomorphometric analysis. To provide greater clarity and flexibility, this language has been revised to allow for use of "*a tetracycline (e.g., tetracycline hydrochloride ([HCl] and demeclocycline)*."

6. **Echocardiogram**: In Section 7.5.6.7, the procedure for assessments of echocardiograms (ECHO) has been revised to central reading.

   Rationale: ECHO results will be read centrally rather than at local sites to ensure consistent criteria across the study are used to assess ECHO parameters by trained personnel. Clinically significant abnormalities detected following central ECHO reading will be promptly reported to the principal investigator for attention as indicated.

7. **Overview of the Disease and Summary of Overall Risks and Potential Benefits:** In Section 5.2 and Section 5.4, the disease information for TIO/ENS and the summary of overall risks and potential benefits of KRN23 have been updated.

   Rationale: The disease description of TIO/ENS and the overall risks and potential benefits of KRN23 were revised to reflect the recently updated KRN23 Investigator's Brochure (IB) (19 November 2015).

CLINICAL STUDY PROTOCOL AMENDMENT

SUMMARY OF CHANGES AND RATIONALE

UX023T-CL201 Amendment 4

10 November 2017

Protocol UX023T-CL201 Amendment 3 (dated 17 February 2016) has been modified by Amendment 4 to increase the duration of the Treatment Extension period and add safety follow-up telephone calls; update and clarify the Schedule of Events; update the clinical study background information; update the contraception requirements; update the maximum injection volume of KRN23; update the safety reporting language; and make administrative updates regarding the medical monitor and record retention language. Minor edits and typographical corrections have also been made. Important protocol changes are summarized below:

1. **Study Duration**: Section 7.1 and related sections have been updated to extend the treatment duration from 140 weeks to up to 216 weeks or until June 30, 2019, plus an additional safety follow-up period.

   Rationale: The study is being extended until the approximate expected date of commercial drug availability to allow continuation of treatment in these subjects.

2. **Investigational Product Administration**: Section 7.4.2.1 has been updated to increase the maximum volume of KRN23 that may be administered in a single injection from 1.0 to 1.5 mL and to indicate that rotation of injections may include rotation to a different quadrant of the abdomen.

   Rationale: This modification in dosing volume will allow more flexibility in subcutaneous administration of investigational product and more specific information about the dosing procedures.

3. **Introduction**: In Section 5, Information on Previous and Ongoing Clinical Studies (Section 5.3.3) and Summary of Overall Risks and Potential Benefits (Section 5.4) have been updated to incorporate the most recent clinical trial data from the X-linked hypophosphatemia (XLH) program.

   Rationale: Updated to reflect the currently available clinical data on KRN23 use in XLH and TIO.

4. **Removal of Subjects from Therapy or Assessment**: Section 7.3.3 has been updated to indicate that a subject may be removed if she becomes pregnant during the study or if a previously unidentified causal tumor is identified and determined to be amenable to surgical removal.

   Rationale: Experience with KRN23 in pregnant women is limited and the study drug may involve risks to a pregnant female or unborn baby that are currently unknown. Therefore, use of a highly effective method of contraception is required during the study, and

subjects who become pregnant during the study will be discontinued as a safety precaution. Removing a subject from study who is determined to have a tumor that is amenable to complete resection is consistent with the inclusion criterion requiring a tumor that is not amenable to cure by surgical excision.

5. **Study Procedures and Assessments**: Several changes have been made to Section 7.5 and the Schedules of Events, and a new Schedule of Events Table 2.4 has been added to describe assessments during Weeks 145 through the End of Study (EOS) visit and the Safety Follow-up period.

    a. In Section 7.5 and the Schedules of Events for Screening and Baseline and the Treatment Period (Table 2.1 and Table 2.2), the Day −21, −20, and −19 time points before the Baseline and Week 48 Day 2 Visits at which subjects should take the first course of tetracycline have been revised to Days −20, −19, and −18 before those visits.

       Rationale: The time points for administration of the first course of tetracycline before biopsy have been made consistent with the Transiliac Bone Biopsy Manual.

    b. A Home Health visit has been added at Week 142 for collection of peak serum phosphorous levels. Adverse events (AEs), vital signs, and concomitant medications will also be recorded. In addition, peak serum phosphorus levels will be collected during the new portion of the extension period (Weeks 145 – End of Study) at Weeks 166, 190, and 214.

       Rationale: To obtain peak serum phosphorus levels at time points later in the study.

    c. Physical Examinations at Home Health visits have been removed during the treatment extension period. Physical Examinations will continue to be performed at all site visits.

       Rationale: Removing physical examinations will streamline Home Health visits as the physical exam is not needed at such frequency.

    d. Anti-KRN23 antibody assessments have been added at site visits (ie, every 24 weeks) during the treatment extension period.

       Rationale: The addition of anti-KRN23 antibody assessments will allow ongoing monitoring of potential anti-KRN23 antibody formation in subjects during the treatment extension period of the study.

    e. Serum KRN23 concentration assessments have been added at site visits (ie, every 24 weeks) during the treatment extension period.

       Rationale: The addition of serum KRN23 assessments will allow ongoing monitoring of KRN23 concentrations during the treatment extension period of the study.

f. In Section 7.5.6.9 and the Schedules of Events the frequency of tumor imaging during the treatment extension period has been changed from once every 6 months to once every 12 months. In addition, language has been added to indicate that a historical tumor image obtained within 12 months of Screening may be used as the Screening image. The section has also been updated to indicate that when applicable, mint Lesion™ software, rather than RECIST, will be used to assess changes in tumor size and volume over time.

   Rationale: Reducing the frequency of tumor imaging decreases subject burden and remains in line with standard medical practice of imaging every 6-12 months. Similarly, use of a historical tumor image within 12 months is consistent with medical practice. Use of the mint Lesion software™ reflects a software update and is most applicable to the timing and location of tumor imaging in the study.

g. Section 7.5.6.9 and the Schedules of Events have been updated to indicate that for subjects with tumor-induced osteomalacia (TIO) in whom the tumor had not been located at baseline, yearly evaluation will be performed beginning at Week 120 in an attempt to locate the tumor. The preferred imaging technique is ^{68}Ga-DOTATATE positron emission tomography (PET)/computed tomography (CT). Octreotide-SPECT (SPECT/CT) or FDG-PT/CT may be used if DOTATATE PET/CT is not available. (If none of these methods is available, another imaging technique may be used at investigator discretion.)

   Rationale: The protocol previously did not specify ongoing attempts at tumor identification during the study in those subjects for whom tumors were not identified at baseline. This change clarifies that imaging for potential tumor identification will be ongoing during the study.

h. Dual-energy X-ray absorptiometry (DXA) assessment of the wrist has been removed.

   Rationale: Wrist DXA was added in Amendment 2 but not implemented so it is being removed. DXA of the lumbar spine and hip will continue to be performed through Week 144.

i. Ambulatory status has been added to the Schedule of Events and to Section 7.5.4.4. Ambulatory status at baseline will be retrospectively collected; prospective collection of ambulatory status will begin at the first scheduled site visit following implementation of and consent to this amendment and will continue at all subsequent site visits.

   Rationale: This addition will allow evaluation of functional disability in these subjects with TIO and any changes with KRN23 treatment.

j. Serial photographs of skin lesions in subjects with epidermal nevus syndrome (ENS)-associated osteomalacia have been removed as an assessment. Dermatologic assessment will continue to be performed as part of the physical examination.

Rationale: Administrative decision given only one subject with ENS-associated osteomalacia is enrolled in the study.

6. **Study Endpoints:** In Section 7.6.1 the wording of the co-primary endpoint regarding histomorphometry assessments has been updated to be: The **change** from baseline in excess osteoid based on analysis of iliac crest bone biopsies after 48 weeks of KRN23 treatment using the following histomorphometric indices: O.Th, OS/BS, OV/BV, and MLt. Previously the endpoint had specified **percent change** from baseline. In addition, Secondary endpoints of additional measures to assess serum phosphorus levels over time have been updated to indicate that these reflect a Week 24 time point. In addition, in the synopsis, the description of exploratory endpoints was updated to align with the description of these endpoints in Section 7.6.1 and with the Statistical Analysis Plan.

   Rationale: Wording was updated to align with the Statistical Analysis Plan v 1.0 (dated 04 May 2016).

7. **Treatments**: Sections 7.1, 7.4.1, and 7.4.5 have been updated to indicate that if a subject undergoes treatment of the underlying tumor (ie, radiation therapy or excision) at any point during the study, KRN23 treatment should be interrupted. If the investigator determines, based on serum phosphorus levels below the lower level of normal in the subject, that KRN23 treatment should be resumed, the dose should be restarted at 0.3 mg/kg Q4W and titrated to a new maintenance dose based on peak and trough serum phosphorus levels, up to a maximum dose of 2.0 mg/kg.

   Rationale: Radiation therapy or tumor excision has the potential to reduce tumor activity or tumor ability to produce FGF23, thereby altering previous FGF23 levels. Therefore, resuming KRN23 treatment at a low dose and re-titrating to a new maintenance dose is being implemented as a safety measure.

8. **Prohibited Medications:** In Section 7.4.7.1, an exception has been added to allow use of oral phosphate and/or pharmacologic vitamin D metabolites in the case of a subject who needs to discontinue treatment with KRN23 for more than 4 weeks to undergo treatment of the underlying tumor.

   Rationale: This change has been added in conjunction with the requirement that a subject discontinues KRN23 treatment if they will undergo treatment for the underlying tumor (see Summary of Changes #7) as management of hypophosphatemia may still be needed in this circumstance.

9. **Anti-KRN23 Antibodies:** In Section 7.5.6.5, the term HAHA (human anti-human antibody) in reference to anti-KRN23 antibody testing has been replaced with the term ADA (anti-drug antibody). The assay methodology has also been updated.

   Rationale: This change is a clarification. The immunogenicity of KRN23 is evaluated by quantifying total ADA, independent of isotype, in human serum. While the study protocol previously used the term “HAHA” for this assessment, it has been replaced with the more correct and specific term, ADA. The updated assay methodology language reflects the current assay.

10. **Contraception Methods:** In Section 7.5.6.12, the list of examples of highly effective contraception methods was updated. In addition, Section 7.3.3 was updated to indicate that subjects who become pregnant will be discontinued from study.

    Rationale: These change was made to align the acceptable methods of contraception in compliance with the Clinical Trial Facilitation Group (CTFG) guideline, "Recommendations related to contraception and pregnancy testing in clinical trials" and to be consistent with clinical trials in adults with XLH.

11. **Data Quality Assurance and Record Retention:** Sections 8.4.2 and 8.4.3 have been updated to state that all study records must be retained for at least 25 years after the end of the trial or in accordance with national law and the right of regulatory authorities, the IRB, and/or Ultragenyx or its designees to access these records will also be retained for at least 25 years or in accordance with national law.

    Rationale: This administrative change has been made to reflect regulations by health authorities.

12. **Safety Assessments**: In the Synopsis, electrocardiogram (ECG) is now listed as a general safety assessment. Previously it was listed within the safety assessments for ectopic mineralization.

    Rationale: ECG is performed to evaluate for changes associated with left ventricular hypertrophy. Ectopic mineralization is not expected to affect ECG parameters and ECG was inadvertently listed in that section in the original protocol. Ectopic mineralization in the heart will be assessed by centrally read ECHOs.

13. **Reporting and Follow-up of Adverse Events**. Section 8.5 and subsections have been updated to indicate that wherever possible, the severity of all AEs will be graded using the NCI CTCAE version 4.0. In addition, language regarding procedures for AE reporting to Ultragenyx have been updated as well as the name and contact information for the medical monitor.

    Rationale: These updates to AE reporting and follow up were made to align with current guidelines and clarify current practices. The update to the medical monitor contact information was an administrative change.

CLINICAL STUDY PROTOCOL AMENDMENT

SUMMARY OF CHANGES AND RATIONALE

UX023T-CL201 Amendment 5

27 April 2018

Protocol UX023T-CL201 Amendment 4 (dated 10 November 2017) has been modified by Amendment 5 to extend the study duration until December 2019, to clarify procedures for dose optimization in later stages of the study, to allow Q2W dosing in certain cases, to add and clarify study procedures, and to note US and EU approval of KRN23 for the treatment of X-linked hypophosphatemia (XLH), a separate indication. Minor edits and typographical corrections have also been made. Important protocol changes are summarized below:

1. **Study Duration**: Section 7.1 and related sections have been updated to extend the treatment duration from up to 216 weeks or June 30, 2019 to up to 244 weeks or until December 31, 2019, plus an additional safety follow-up period. These additional study visits are reflected in the Schedule of Events Table 2.5.

   Rationale: The study is being extended until the approximate expected date of commercial drug availability to allow continuation of treatment in these subjects.

2. **Selection and Timing of Doses**: Dose adjustment beyond the 16-week titration period is permitted during the study. Section 7.4.5 and related sections have been updated to clarify the procedures related to these later dose adjustments. Specifically, if fasting serum phosphorus levels at trough (i.e., 4 weeks after a dose and before the subsequent dose) remain below the lower limit of normal (< 2.5 mg/dL), the dose may be increased in 0.5 mg/kg increments up to a dose of 2.0 mg/kg Q4W. In addition, if trough levels of serum phosphorus remain < 2.5 mg/dL after the dose is increased to 2.0 mg/kg Q4W, the dose frequency may be increased to every 2 weeks (Q2W). In these subjects, Q2W dosing will be initiated at 60% of the Q4W total dose. Serum phosphorus levels will be measured every 4 weeks (prior to subsequent doses) for 12 weeks and if levels remain < 2.5 mg/dL, the dose may be increased to 2.0 mg/kg Q2W, the maximum dose.

   Rationale: Clarifying the procedures for dose adjustment later in the study will provide additional guidance for investigators to manage dosing in subjects. Allowing an increased dose frequency to Q2W in subjects who do not maintain a trough serum phosphorus within the normal range at a dose of 2.0 mg/kg Q4W will allow evaluation of whether more frequent dosing can maintain serum phosphorus levels within the normal range.

3. **Study Assessments:** (a) A supplemental Schedule of Events has been added (Table 2.6) to identify additional assessments to be performed for subjects who have a dose optimization at or after Week 96. (b) The Sit to Stand (STS) test has been added to Table 2.4 and Table 2.5 only for those subjects who have a dose optimization at or after Week 96. (c) Home health visits at Weeks 166, 190, and 214 have been changed to site visits to allow assessment of PROs and the STS. (d) Assessment of serum concentrations of KRN23 in stable samples has been added at Weeks 8, 32, 36, and 40 in Table 2.2.

(e) Section 7.5.6.9 has been clarified to indicate that in subjects for whom a tumor was not identified at baseline but who have the tumor located by 68Ga-DOTATATE PET/CT imaging or another method while on study, MRI or CT should be performed within 21 (±7 days) days of tumor identification to assess tumor size. Thereafter, tumor imaging should be performed using the same method (MRI or CT) according to the Schedules of Events. If the MRI/CT imaging following tumor identification has occurred within 3 months of the next scheduled MRI/CT assessment, that assessment will be skipped and the Schedules of Events will be followed thereafter. (f) A radiographic skeletal survey has been added to the Schedule of Events at Week 144 in Table 2.4. (g) Genetic testing to evaluate for mutations in PHEX, the gene responsible for XLH, in subjects with TIO symptoms from childhood and for whom the tumor has not been identified has been added to the Schedule of Events in Table 2.3.

Rationale: (a), (b), and (c) The additional assessments at the start of and following dose optimization include serum phosphorus measurements, PRO assessments (Brief Pain Inventory, Brief Fatigue Inventory, and SF-36), and functional assessments (STS test). These assessments will allow evaluation of the new dose on how the subject feels and functions both at the mid-point (with Q4W dosing) and at the end of the dose cycle.

(d) The assessment of serum KRN23 concentrations at Weeks 8, 32, 36, and 40 were included in a previous version of the protocol and removed in Amendment 3. The assessments are being reinstated to enable retrospective evaluation of stable stored study samples collected at these time points to allow a more complete assessment of the relationship between serum phosphorus, KRN23 dose, and KRN23 concentration.

(e) This is a clarification that the MRI or CT to assess tumor size should be performed within 21 (±7) days of identification by ^{68}Ga-DOTATATE PET/CT imaging and then according to the Schedule of Events. Previously it stated imaging should be performed according to the Schedule of Events.

(f) Addition of the skeletal survey at Week 144 will enable a full evaluation of fractures and pseudofractures at a time point when bone scans are also performed. This may allow a better assessment of the effects of KRN23 on fracture burden in these subjects with TIO or ENS.

(g) Mutational analysis of the PHEX gene has been added to rule out a possible XLH diagnosis due to the similar clinical features of the diseases in specific subjects who have had clinical symptoms and hypophosphatemia since childhood and in whom the tumor has not been identified.

4. **Contraception Requirement**: In Section 7.3.1, language regarding contraception in Inclusion Criteria #8 and #9 has been updated for consistency with changes made previously to other parts of the protocol.

   Rationale: The definition of women not of childbearing potential was updated to align with the Clinical Trial Facilitation Group (CTFG) guideline. The change to a single highly effective form of contraception had previously been made to Section 7.5.6.12 (Pregnancy Testing and Contraception) but not to the Inclusion criteria because the study

was fully enrolled. The current update was made for the purpose of consistency throughout the protocol.

5. **Coordinating Investigator**: In Section 8.2, language has been added to detail selection and roles of a Coordinating Investigator for the study. Dr Suzanne Jan De Beur has been named as the Coordinating Investigator on the title page.

   Rationale: This change was made to indicate that a Coordinating Investigator has been identified for the study.

6. **Timing of Safety Follow-up Telephone Calls:** In Section 7.1 and related sections, the wording regarding Safety Follow-up Telephone Calls has been modified. The timing of the telephone calls is now based on the timing of the last dose of study drug; previously it had been based on the timing of the EOS/ET visit. The Safety Follow-up Telephone Calls will now be performed at 6 weeks (+5 days) and 12 weeks (+ 5 days) after the subject's last dose of study drug. In addition, the first safety follow up call will now be performed for all subjects, not only in those not continuing KRN23 treatment through commercial use or another mechanism upon completion of study.

   Rationale: Changing the timing of the Safety Follow-up Telephone Calls to be based on the last dose of study drug was done to allow consistency of timing from last dose, given that some subjects may be receiving Q2W dosing. All subjects will be called as a conservative approach to ensure no safety data are missed.

7. **Regulatory Approvals of KRN23 (burosumab) for XLH:** In Section 5.1, the FDA approval of burosumab for the treatment of XLH in adults and pediatric patients 1 year of age and older and conditional marketing authorization in the EU for the treatment of XLH with radiographic evidence of bone disease in children 1 year of age and older and adolescents with growing skeletons have been noted.

   Rationale: This change was made to update the Background section with the most current regulatory status of KRN23.

CLINICAL STUDY PROTOCOL AMENDMENT

SUMMARY OF CHANGES AND RATIONALE

UX023T-CL201 Amendment 6

26 September 2019

Protocol UX023T-CL201 Amendment 5 (dated 27 April 2018) has been modified by Amendment 6 to incorporate the following changes. Minor edits and typographical corrections have also been made as needed.

1. **Study duration:** Section 7.1 and related sections were updated to extend the total treatment duration from up to 244 weeks or 31 December 2019, to up to 300 weeks or 31 January 2021, or until commercial availability of KRN23 as prescribed by subjects' treating physician, whichever is sooner. Accordingly, the End of Study (EOS) assessments were removed from Week 244, and the additional study visits along with the revised EOS assessments are now shown in the Schedule of Events Table 2.6.

   Rationale: The study is being extended to allow continuation of treatment in subjects until availability of another mechanism of KRN23 treatment, such as commercially available treatment.

2. **Timing of Safety Follow-up Telephone Call:** The Schedule of Events Table 2.6, Section 7.1, and related sections were updated to indicate that the Safety Follow-up Telephone Call (TC) will occur approximately 6 weeks (+ 5 days) after a subject's last dose of study drug to collect information on any ongoing or new adverse events (AEs), serious adverse events (SAEs), and concomitant medications. An additional Safety Follow-up TC will occur approximately 12 weeks (+ 5 days) after the subject's last dose of study drug. If subjects have started receiving KRN23 (burosumab) via an alternative mechanism (eg, commercial treatment) by the time of the 6-week Safety Follow-up TC, the 12-week Safety Follow-up TC may not be necessary.

   Rationale: To clarify the timing of Safety Follow-up TCs with respect to subjects' last dose of study drug.

3. **Supplemental Schedule of Events table:** The supplemental Schedule of Events table that outlined the assessments to be performed following a dose optimization at or after Week 96, including serum KRN23, brief pain inventory (BPI), brief fatigue inventory (BFI), 36-item short form health survey (SF-36), and sit-to-stand (STS), was removed from the Schedule of Events and related sections throughout protocol.

   Rationale: To simplify the study assessments and minimize unnecessary repetition of assessments.

4. **Schedule of Events Tables 2.4 and 2.5:** Visits at Weeks 142, 166, 192, and 214 were removed from the Treatment Extension Period.

   Rationale: These visits were included in the protocol when dosing every 2 weeks (Q2W) was added to the study. However, the previous version of the protocol did not indicate that these additional visits are only for subjects who are undergoing Q2W dosing. This change is a clarification to avoid unnecessary site visits by all subjects.

5. **Sit-to-stand assessments during the Treatment Extension Period:** STS assessments were removed from the Treatment Extension Period (Schedule of Events Table 2.4 and Table 2.5) for subjects who have an upward dose or frequency adjustment at or after Week 96.

   Rationale: To simplify the study assessments in subjects undergoing dose optimization at or after Week 96.

6. **Radiographic assessments:** Section 7.5.4.3, the Schedule of Events, and related sections were updated to clarify that targeted radiographs will be completed at the anatomical location where a fracture or pseudofracture was identified at or after Screening every 24 weeks starting at Week 72, until resolution, or up to Week 240.

   Rationale: To clarify the timing of follow-up of fractures or pseudofractures identified at or after Screening. Previously, radiographic assessments were not included after Week 144.

7. **Tumor imaging and tumor identification:** Section 7.5.6.9 and the related assessments in the Schedule of Events (Table 2.6) were modified to clarify that for subjects with tumor-induced osteomalacia (TIO), if the tumor is visible and localized at Screening, it will be imaged at Screening and every 24 weeks through Week 144, and then every 48 weeks (or at next scheduled visit) for the remainder of the study. For subjects with TIO whose tumor was not identifiable at Screening, evaluations every 48 weeks (or in alignment with regularly scheduled clinic visits) will be performed beginning at Week 120 in an attempt to locate the tumor. The preferred imaging technique is ^{68}Ga-DOTATATE positron emission tomography (PET)/computed tomography (CT). Imaging to locate the tumor may also be performed at unscheduled visits if there is clinical suspicion of new tumor development. If the tumor is located and determined to be unresectable, magnetic resonance imaging (MRI) or CT imaging (at the discretion of the Investigator) should occur within 21 (± 7) days of identification to assess tumor size, at an unscheduled visit if necessary, and thereafter according to the Schedule of Events.

   Rationale: To clarify tumor imaging and tumor identification assessments throughout the remainder of the study.

8. **Definition of maintenance dose:** In Section 7.4.5 and related sections, a definition of maintenance dose was added (defined as a dose at which 2 consecutive trough serum phosphorous levels are within the normal range [2.5 – 4.0 mg/dL]).

   Rationale: To provide clarity on the criterion for a maintenance dose of KRN23.

9. KRN23 dose titration schemes:

(a) Table 7.4.5.1 was revised to include only the initial KRN23 dose titration scheme; dosing procedures for subjects undergoing treatment of an underlying tumor were removed and placed in Section 7.4.5.1 and Table 7.4.5.3.

(b) Table 7.4.5.2 and related text were updated to clarify the criteria and dose titration scheme for dose adjustments and increases in dose frequency at or after Week 96. Criteria for dose adjustments based on trough serum phosphorous levels were added. For subjects increasing to a KRN23 dose of 2.0 mg/kg Q2W based on the criteria in Table 7.4.5.2, additional predose serum phosphorus measurements were added every 4 weeks (Q4W) for 12 weeks to ensure that subjects do not experience dose-limiting toxicity (DLT) (Section 7.5.6.13).

(c) Section 7.4.5.1 and Table 7.4.5.3 were created to clarify dosing procedures and the dose titration scheme for subjects who undergo treatment of an underlying tumor. Language was added to clarify that serum phosphorus levels should be monitored every 4 weeks for 24 weeks following the resumption of KRN23 treatment through unscheduled blood draws at peak time points. The KRN23 dose will be titrated based on peak serum phosphorous levels as indicated in Table 7.4.5.3. Thereafter, dosing decisions will be made based on trough serum phosphorus levels as indicated in Table 7.4.5.2, and serum phosphorus measurements will proceed according to the Schedule of Events.

Rationale: (a, b, c) To clarify the study dosing procedures, dose titration schemes, and related assessments.

10. Introduction: Previous and Ongoing Clinical Studies (Section 5.3.3), and Summary of Overall Risks and Potential Benefits (Section 5.4) were updated to reflect the current information from clinical studies of KRN23.

Rationale: To update the protocol with the most current clinical data for KRN23.

11. Stopping rules: Minor updates were made to the Stopping Rules (Section 7.3.3.1) to clarify the criteria under which study treatment will be stopped, and/or the subject will be discontinued from the study.

Rationale: To more clearly define the Stopping Rules for this study.

12. Medical Monitor: In Section 8.5.4.6, the study Medical Monitor was changed to Mary Scott Roberts, MD.

Rationale: This was a minor administrative change to update the safety contact information to reflect the current Medical Monitor for the study.

13. Study safety review team: References to the study safety review team in Section 7.1, Section 7.3.3.1, and Section 7.5.6.13 were changed to clarify that the Sponsor will perform these safety monitoring activities. Key safety data will be reviewed by the Sponsor periodically on an ongoing basis coincident with formal data snap shots.

Rationale: To clarify the language regarding the safety monitoring activities performed by the Sponsor.

2 SYNOPSIS

**TITLE OF STUDY:**<br><br>A Phase 2 open-label trial to assess the efficacy and safety of KRN23, an antibody to FGF23, in subjects with tumor-induced osteomalacia (TIO) or epidermal nevus syndrome (ENS)-associated osteomalacia
**PROTOCOL NUMBER**:<br><br>UX023T-CL201
**STUDY SITES:**<br><br>Multiple study sites in the United States
**PHASE OF DEVELOPMENT:**<br><br>Phase 2
**RATIONALE FOR THIS STUDY:**<br><br>KRN23 is a fully human immunoglobulin G_1 (IgG_1) monoclonal antibody (mAb) that binds to and inhibits the activity of fibroblast growth factor 23 (FGF23), leading to an increase in serum phosphorus levels. There are multiple disorders (each with a unique underlying cause) that result in unusually high circulating levels of FGF23, which in turn result in renal phosphate wasting and reduced (or aberrantly normal in relationship to elevated FGF23) levels of 1,25-dihydroxyvitamin D (1,25[OH]$_2$D). Across these disorders the clinical symptoms are similar and often include osteomalacia (and, in children, rickets), muscle weakness, fatigue, bone pain, and fractures. KRN23 has been studied in 1 of these disorders, X-linked hypophosphatemia (XLH). Positive results were observed in a nonclinical pharmacology model of XLH. In single- and repeat-dose clinical studies in subjects with XLH, subcutaneous (SC) administration of KRN23 consistently increased and sustained serum phosphorus levels and tubular reabsorption of phosphate (TRP) without a major impact on urine calcium levels or vitamin D metabolism. KRN23 (burosumab) was approved by the Food and Drug Administration (FDA) on 17 April 2018 for the treatment of XLH in adult and pediatric patients 1 year of age and older, and a positive European Commission conditional marketing authorization was received on 23 February 2018 for the treatment of XLH with radiographic evidence of bone disease in children 1 year of age and older and adolescents with growing skeletons.

Similar to XLH, tumor-induced osteomalacia (TIO), also known as oncogenic osteomalacia, is an acquired condition in which tumors, whose pathology is usually phosphaturic mesenchymal tumors (PMT), cause excessive ectopic production of FGF23 that leads to renal phosphate wasting and impaired vitamin D synthesis. The high levels of FGF23 result in a similar biochemical profile to XLH that includes low serum phosphorus, phosphaturia, an abnormally low ratio of renal tubular maximum phosphate reabsorption rate to glomerular filtration rate (TmP/GFR), elevated alkaline phosphatase (ALP), normal calcium, and low to normal levels of 1,25(OH)$_2$D. In TIO, tumors that are typically benign cause excessive ectopic production of FGF23, and if the causal tumor can be completely removed, the syndrome remits. However, some tumors are considered inoperable because of the location of the tumor or extent of disease or an inability to localize the tumor.

Epidermal nevus syndrome (ENS) are rare congenital syndromes characterized by the presence of epidermal nevi in association with 1 or more other developmental abnormalities of other organ systems including the nervous, skeletal, cardiovascular, and ocular systems. Hypophosphatemia may rarely be 1 of the skeletal manifestations of ENS presenting in children primarily as rickets and in adults as osteomalacia. In all ENS-associated osteomalacia (also known as cutaneous skeletal hypophosphatemia syndrome) cases in which serum FGF23 levels were assessed, they were found to be elevated. The source of the excess FGF23 in ENS-associated osteomalacia is unclear. While early studies suggested it may be from the skin lesions themselves, more recent studies suggest it may be skeletally derived. Compared with TIO, complete resection and surgical cure leading to removal of the source of excess FGF23 is less likely. Nonetheless, published case reports of ENS-associated osteomalacia indicate improvement with skin debridement surgery in some cases. In many cases, improvement, if any, is modest and transient.

In both TIO and ENS-associated osteomalacia, when surgery is not curative or feasible, medical treatment comprises oral phosphate and/or active vitamin D replacement. Efficacy of these treatments is often limited; it does not treat the underlying disease; and its benefits must be balanced with monitoring for potential risks such as nephrocalcinosis, hypercalciuria, and hyperparathyroidism.

This is a Phase 2 study designed to assess the efficacy, safety, pharmacokinetics (PK), and pharmacodynamics (PD) of KRN23 in subjects with TIO whose tumor is inoperable (defined as ‘the inability to localize the tumor or the impractical nature of surgery due to the extent of disease or the location of the tumor’) or ENS-associated osteomalacia, where skin lesion removal therapy is not considered a valid treatment option. It is hypothesized that KRN23 may provide clinical benefit in this patient population because of the common underlying feature in patients with TIO or ENS-associated osteomalacia and in patients with XLH: abnormally elevated FGF23 levels.

OBJECTIVES:

The primary objectives of this study are to evaluate the following:

- Effect of KRN23 treatment on increasing serum phosphorus levels in adults with TIO or ENS-associated osteomalacia
- Effect of KRN23 treatment on improvement in TIO/ENS-associated osteomalacia as determined by the following histomorphometric indices:
    - Osteoid Thickness (O.Th)
    - Osteoid surface/Bone surface (OS/BS)
    - Osteoid volume/Bone volume (OV/BV)
    - Mineralization lag time (MLt)

Secondary objectives of the study are to evaluate the following:

- The PD profile of KRN23 as assessed by changes from Baseline over time in additional measures of serum phosphorus, serum FGF23, ALP, and 1,25$(OH)_2$D; TRP and TmP/GFR (the ratio of renal tubular maximum phosphate reabsorption rate to glomerular filtration rate)
- Effects of KRN23 on bone turnover markers, including bone-specific ALP (BALP), carboxy terminal cross-linked telopeptide of type I collagen (CTx), procollagen type 1 N-propeptide (P1NP), and osteocalcin
- Functional outcomes including upper and lower extremity muscle strength, walking and reaching ability, and mobility
- Patient-reported outcomes including self-reported pain, fatigue, and health-related quality of life

The PK objective of the study is to:

- Determine the PK profile of repeat SC injections of KRN23 at Baseline (Weeks 0, 2, and 4) and 6 Months (Weeks 20, 22, and 24) in subjects with TIO or ENS-associated osteomalacia

Exploratory objectives of the study are to evaluate the following:

- Changes in additional histomorphometry parameters in trans-iliac crest bone biopsies including both structural and dynamic measures
- Changes in underlying skeletal disease/osteomalacia as assessed by skeletal survey/standard radiographs, dual-energy X-ray absorptiometry (DXA), ^{99m}Tc-labelled bone scan, and high-resolution peripheral quantitative computed tomography (CT) (XtremeCT; where available)

The safety objective of the study is to:

- Assess the safety of KRN23 administration in subjects with TIO or ENS-associated osteomalacia, based on adverse events (AEs), laboratory assessments, cardiac imaging, renal ultrasound, and immunogenic response

STUDY DESIGN AND METHODOLOGY:

UX023T-CL201 is a Phase 2 open-label study to determine the efficacy, safety, PD, and PK of repeat SC injections of KRN23 in adult subjects with TIO or ENS-associated osteomalacia.

Subjects will be selected for Screening only if they are deemed to have inoperable disease (defined by an inability to localize the tumor or the impractical nature of surgery due to the extent of disease or the location of the tumor) or if they have ENS-associated osteomalacia and are not being considered for skin lesion removal treatment. Subjects who are eligible for Screening will discontinue oral phosphate and vitamin D metabolite therapy at least 2 weeks prior to Screening and may not resume supplementation for the duration of the study. Subjects who are eligible for study entry will receive tetracycline (eg, tetracycline hydrochloride [HCl] and demeclocycline) prior to the Baseline Visit to facilitate histomorphometric analysis of the Baseline trans-iliac crest bone biopsy (refer to Section 7.5.4.3).

The Baseline Visit will be conducted over approximately 4 days, including overnight stay if deemed necessary by the Investigator. Baseline safety and efficacy assessments (*excluding bone biopsy*) will occur on the first 1 to 2 days (Days −3 and −2). On the following day (Day −1), a trans-iliac crest bone biopsy will be performed (refer to Section 7.5.4.3). On the next day (Day 0, Week 0), subjects will receive their first SC injection(s) of KRN23.

All enrolled subjects will begin treatment with KRN23 every 4 weeks (Q4W) at a starting dose of 0.3 mg/kg (Week 0). Doses of KRN23 will then be titrated in an effort to achieve a target fasting serum phosphorus range of 2.5 to 4.0 mg/dL (Table 7.4.5.1). All serum phosphorus assessments will be taken with subjects in the fasting state. If the subject's peak serum phosphorus level at Week 2 remains below the target range, the subject's dose may be increased at Week 4 to 0.6 mg/kg, at the discretion of the Investigator. Doses will then continue to be titrated in increments of 0.2 mg/kg at Weeks 8, 12, and 16 in individual subjects to achieve the target peak serum phosphorus range. Doses may be titrated at later visits, at the discretion of the Investigator, if there are concerns about safety or suboptimal efficacy or if a subject has not yet achieved the target range of serum phosphorus.
The maximum dose allowed in this protocol is 2.0 mg/kg every 2 weeks (Q2W). If needed, the final dose adjustment increment may be less than the specified increment to reach the 2.0 mg/kg dose. If a subject undergoes treatment of the underlying tumor (ie, radiation therapy or excision) at any point during the study, KRN23 treatment should be interrupted (Section 7.4.5.1). If the Investigator with the study Medical Monitor determines, based on serum phosphorus below the lower limit of normal, that the subject should resume KRN23 treatment, the dose should be restarted at 0.3 mg/kg Q4W. Serum phosphorus levels should be subsequently monitored every 4 weeks for 24 weeks following the resumption of KRN23 treatment through unscheduled blood draws at peak (2 weeks postdose) time

points. The KRN23 dose will be titrated based on peak serum phosphorous levels as indicated in Table 7.4.5.3. Thereafter, dosing decisions will be made based on trough serum phosphorus levels (4 weeks ± 3 days postdose and prior to the subsequent dose) obtained at the previous study visit as indicated in Table 7.4.5.2, and serum phosphorus assessments will proceed according to the Schedule of Events (Table 2.1, Table 2.2, Table 2.3, Table 2.4, Table 2.5, and Table 2.6).

Subjects will be enrolled consecutively. Key safety data will be reviewed periodically on an ongoing basis coincident with formal data snap shots. If a dose-limiting toxicity (DLT; a Grade ≥ 3 toxicity that is probably or possibly treatment related or a confirmed serum phosphorus level ≥ 6.5 mg/dL) is identified at any point, the study will be paused until all of the efficacy and safety data collected to date are reviewed to evaluate the overall risks/benefits of the study and to make a determination about whether it will continue.

Screening, Baseline, and post-treatment PK, PD, efficacy, and safety assessments are shown by study visit in Table 2.1, Table 2.2, Table 2.3, Table 2.4, Table 2.5, and Table 2.6.

Upon regulatory approval and commercial availability of KRN23 for the treatment of TIO, subjects will have their End of Study (EOS) Visit, and transition to prescription drug, as recommended by their treating physician. Until commercial availability, subjects may continue treatment with KRN23 for up to 252 weeks in the Treatment Extension Period, or until 31 January 2021, whichever is sooner, and then undergo their EOS Visit. Therefore, the duration of the Treatment Extension Period will vary for individual subjects, and will be determined by the time from the start of Week 49 through their EOS Visit. Upon completion of study drug treatment (ie, EOS Visit) or early withdrawal from this study (ie, Early Termination [ET] Visit), a Safety Follow-up Telephone Call (TC) will occur approximately 6 weeks (+ 5 days) after a subject’s last dose of study drug to collect information on any ongoing or new AEs, serious adverse events (SAEs), and concomitant medications. An additional Safety Follow-up TC will occur approximately 12 weeks (+ 5 days) after the subject’s last dose of study drug. If subjects have started receiving KRN23 (burosumab) via an alternative mechanism (eg, commercial treatment) by the time of the 6-week Safety Follow-up TC, the 12-week Safety Follow-up TC may not be necessary. The end of this study is defined as the last day protocol-specified assessments (including telephone contact) are conducted for the last subject.

NUMBER OF SUBJECTS PLANNED:

Approximately 15 adult subjects will be enrolled in the study. Subjects who withdraw or are removed from the study may be replaced on a case-by-case basis, at the discretion of Ultragenyx and the Investigator.

DIAGNOSIS AND CRITERIA FOR INCLUSION AND EXCLUSION:

Individuals eligible to participate in this study must meet all of the following criteria at Screening:

1. Have a clinical diagnosis of TIO/ENS-associated osteomalacia based on evidence of excessive FGF23 that is not amenable to cure by surgical excision of the offending tumor/lesion (documented by Investigator)
2. Be ≥ 18 years of age
3. Have a fasting serum phosphorus level < 2.5 mg/dL
4. Have serum iFGF23 level ≥ 100 pg/ml by Kainos assay
5. Have a ratio of renal tubular maximum reabsorption rate of phosphate to glomerular filtration rate (TmP/GFR) < 2.5 mg/dL
6. Have an estimated glomerular filtration rate (eGFR) ≥ 60 mL/min (using Cockcroft-Gault formula). Subjects with an eGFR ≥ 30 but < 60 mL/min will be considered eligible so long as in the opinion of the Investigator the decline in renal function is not related to nephrocalcinosis
7. Have a corrected serum calcium level < 10.8 mg/dL
   [Corrected serum calcium = serum calcium in mg/dL + 0.8 × (4 − serum albumin in g/dL)]
8. Females of child-bearing potential must have a negative urine pregnancy test at Screening and Baseline and be willing to have additional pregnancy tests during the study. Females considered not to be of childbearing potential include those who have not experienced menarche, are post-menopausal (defined as having no menses for at least 12 months without an alternative medical cause) or are permanently sterile because of total hysterectomy, bilateral salpingectomy, or bilateral oophorectomy
9. Participants of child-bearing potential or fertile males with partners of child-bearing potential who are sexually active must consent to use a highly effective method of contraception as determined by the site Investigator from the period following the signing of the informed consent through the final Safety Follow-up TC (as defined in Section 7.1)
10. Be willing to provide access to prior medical records to determine eligibility including imaging, biochemical, and diagnostic, medical, and surgical history data
11. Provide written informed consent after the nature of the study has been explained, and prior to any research-related procedures
12. Be willing and able to complete all aspects of the study, adhere to the study visit schedule and comply with the assessments (in the opinion of the Investigator)

Individuals who meet any of the following exclusion criteria will not be eligible to participate in the study:

1. Have a prior diagnosis of human immunodeficiency virus, hepatitis B, and/or hepatitis C
2. Have a history of recurrent infection, a predisposition to infection, or a known immunodeficiency
3. Are pregnant or breastfeeding at Screening or are planning to become pregnant (self or partner) at any time during the study
4. Have participated in an investigational drug or device trial within 30 days prior to Screening or are currently enrolled in another study of an investigational product or device
5. Have used a therapeutic mAb, including KRN23, within 90 days prior to Screening or have a history of allergic or anaphylactic reactions to any mAb
6. Have or a have a history of any hypersensitivity to KRN23 excipients that, in the judgment of the Investigator, places the subject at increased risk for adverse effects
7. Have used a pharmacologic vitamin D metabolite or its analog (eg, calcitriol, doxercalciferol, and paricalcitol), phosphate, or aluminum hydroxide antacids (eg, Maalox® and Mylanta®) within 2 weeks prior to Screening or during the study
8. Have used medication to suppress parathyroid hormone (PTH) (eg, Sensipar®, cinacalcet, calcimimetics) within 2 months prior to Screening
9. Have a history of malignancy within 5 years of study entry with the exception of PMT-MCT (Phosphaturic mesenchymal tumors of the mixed connective tissue type) tumors or non-melanoma skin cancers such as basal cell skin cancer
10. Have donated blood or blood products within 60 days prior to Screening
11. Have a history of allergic reaction to or have shown adverse reactions to a tetracycline (eg, tetracycline HCl and demeclocycline), benzodiazepines, fentanyl or lidocaine
12. Have any condition, which in the opinion of the Investigator and Sponsor, could present a concern for either subject safety or difficulty with data interpretation

INVESTIGATIONAL PRODUCT(S), DOSE, AND MODE OF ADMINISTRATION:

KRN23 is a sterile, clear, colorless, and preservative-free solution in single-use 5-mL vials containing 1 mL of KRN23 (30 mg) at a concentration of 30 mg/mL. KRN23 will be administered without dilution via SC injection to the abdomen, upper arm, or thigh; the injection site will be rotated with each injection, including to a different quadrant of the abdomen. No more than 1.5 mL may be administered in a single injection. If a subject needs more than 1.5 mL per administration, multiple injections must be administered and a different site must be used for each injection.

KRN23 will initially be administered Q4W. Doses will be titrated at Weeks 4, 8, 12, and 16 in individual subjects to achieve a target peak serum phosphorus range of 2.5 to 4.0 mg/dL. Doses may be titrated at later visits, at the discretion of the Investigator, if there are concerns about safety or suboptimal efficacy or if a subject has not yet achieved the target range of serum phosphorus. Dose frequency may be increased to Q2W if trough serum phosphorus levels remain < 2.5 mg/dL at a dose of 2.0 mg/kg Q4W. Doses should be interrupted and may be retitrated if a subject undergoes treatment of the underlying tumor (ie, radiation therapy or excision) at any point during the study. The maximum dose allowed in this protocol is 2.0 mg/kg Q2W. If needed, the final dose adjustment increment may be less than the specified increment to reach a 2.0 mg/kg dose. Following a downward dose adjustment, the Investigator together with the Medical Monitor will determine how and when to dose titrate up.

REFERENCE THERAPY, DOSE AND MODE OF ADMINISTRATION:

This is an open-label study in which all subjects will receive investigational product. No placebo or reference therapy will be administered.

DURATION OF TREATMENT:

The total KRN23 treatment duration on study will be up to 300 weeks, or up to 31 January 2021, whichever is sooner, after which subjects will have their EOS Visit. Upon regulatory approval and commercial availability of KRN23 for the treatment of TIO, subjects will have their EOS Visit, and transition to prescription drug, as recommended by their treating physician. Subjects will undergo an ET Visit if 1 of the following occurs: the subject withdraws consent and discontinues from the study, the subject is discontinued from the study at the discretion of the Investigator or Ultragenyx, or the study is terminated.

CRITERIA FOR EVALUATION:

Primary Efficacy Evaluation:

The study will have co-primary endpoints:

- The proportion of subjects achieving mean serum phosphorus levels above the lower limit of normal (LLN; 2.5 mg/dL [0.81 mmol/L]) at the mid-point of the dose interval (2 weeks after dosing), as averaged across dose cycles between Baseline and Week 24
- The change from Baseline in excess osteoid based on analysis of iliac crest bone biopsies after 48 weeks of KRN23 treatment using the following histomorphometric indices:
    - O.Th
    - OS/BS

- OV/BV
- MLt

Secondary Endpoints:

- Additional measures to assess serum phosphorus levels over time include:
  - Proportion of subjects achieving mean serum phosphorus levels above the LLN (2.5 mg/dL [0.81 mmol/L]) at the end of the dosing cycle (4 weeks after dosing), as averaged across dose cycles between Baseline and Week 24
  - Mid-point of dosing cycle: mean change from Baseline and percent change from Baseline averaged across dose cycles between Baseline and Week 24
  - End of dosing cycle: mean change from Baseline averaged across dose cycles between Baseline and Week 24
  - Cumulative exposure: time-adjusted area under the curve between Baseline and Week 24
- Change from Baseline over time in serum FGF23, ALP, 1,25$(OH)_2$D; and urinary phosphorus, TRP, TmP/GFR
- Change and percent change from Baseline over time in serum biochemical markers of bone turnover, including BALP, CTx, P1NP, and osteocalcin
- Change from Baseline over time in muscle strength as assessed by hand-held dynamometry (HHD), sit-to-stand test (STS), weighted arm lift test (WAL), and 6-minute walk test (6MWT)
- Change from Baseline in brief pain inventory (BPI), brief fatigue inventory (BFI), and 36-item short form health survey (SF-36) over time

Exploratory Endpoints:

- Changes in other measures of structural and dynamic histomorphometry in trans-iliac crest bone biopsies
- Changes in bone condition and healing of prior long bone and pseudo-fractures as assessed by ^{99m}Tc-labelled bone scan
- Changes in bone mineral density and bone mineral content as measured by DXA at the lumbar spine and hip
- Changes in bone mineral density, bone mineral content, bone geometry, and microarchitecture in the cortical and trabecular compartments of the radius and tibia as measured by XtremeCT (when available)

Pharmacokinetics:

- Serum KRN23

Safety:

Safety and tolerability will be evaluated by the incidence, frequency, and severity of treatment-emergent AEs and SAEs, as well as changes from Baseline to scheduled time points in the following general safety variables:

- Vital signs and weight
- Physical examinations
- eGFR
- Serum calcium, phosphorus, intact parathyroid hormone (iPTH), and urinary calcium and creatinine
- Chemistry, hematology, and urinalysis, including additional KRN23/TIO biochemical parameters of interest (serum 25-hydroxyvitamin D [25(OH)D], lipase, amylase, creatinine, and FGF23)
- Tumor imaging (TIO) or dermatologic assessment of skin lesions (ENS-associated osteomalacia)
- Anti-KRN23 antibody formation
- Dose-limiting toxicities, defined as:
    - Unexpected SAEs occurring during treatment considered to be either probably or possibly related to the investigational product.
    - A confirmed serum phosphorus level of ≥ 6.5 mg/dL (defined as hyperphosphatemia) at any time after dosing.
- Concomitant medications
- Urine pregnancy testing
- Electrocardiogram (ECG)

Ectopic Mineralization Safety Assessments include:

- Echocardiogram (ECHO)
- Renal ultrasound

STATISTICAL METHODS:

A full description of the statistical evaluations will be provided in the Statistical Analysis Plan (SAP).

Analysis Populations:

Biopsy Analysis Set: The biopsy analysis set will include enrolled subjects with baseline and follow-up (either Week 48 or ET prior to Week 48) bone biopsy data.

Full Analysis Set: All efficacy (except bone biopsy endpoints), safety, and PK/PD analyses will be performed on the set of all subjects who receive at least 1 dose of investigational product.

Primary Efficacy Analysis:

The proportion of subjects who achieve a serum phosphorus level above the LLN (2.5 mg/dL [0.81 mmol/L]), at 2 weeks after dosing (between baseline and Week 24, on average) will be provided, along with the 2-sided 95% confidence interval. Additional analyses of serum phosphorus including observed values, change from Baseline, percent change from Baseline over time, and area under the curve will be summarized from Baseline to Week 24.

Histomorphometric indices O.Th, OS/BS, OV/BV, and MLt at Baseline and Week 48, and their change from Baseline at Week 48 will be summarized.

Other Pharmacodynamics and Efficacy Analysis:

PD and clinical efficacy endpoints will be summarized at each time point they are collected with descriptive statistics. For continuous variables, the mean, standard deviation, median, minimum, and maximum will be provided. For discrete data, the frequency and percent distributions will be provided. Changes over time and the association of the efficacy with the PD variables will also be summarized and evaluated.

Safety Analysis:

All subjects who receive any amount of study drug will be included in the safety analysis.

All AEs will be coded using the Medical Dictionary for Regulatory Activities (MedDRA). The incidence and frequency of AEs will be summarized by System Organ Class, Preferred Term (PT), severity, and relationship to KRN23 treatment. A by-subject listing will be provided for those subjects who experience a SAE, including death, or experience an AE associated with early withdrawal from the study or study drug treatment.

Clinical laboratory data will be summarized by the type of laboratory test. The frequency and percentage of subjects who experience abnormal clinical laboratory results (ie, outside of reference ranges) and/or clinically significant abnormalities will be presented for each clinical laboratory measurement.

Table 2.1: Schedule of Events – Screening and Baseline

	Screening	Baseline[2]					
**STUDY VISIT**	**Screening Visit**	**TC1[1]**	**TC2[1]**	**Visit 1[3]**			
**STUDY WEEK or STUDY DAY**	**Week −4**	**Week −3**	**Week −1**	**Day −3**	**Day −2**	**Day −1**	**Week / Day 0[4]**
Informed Consent	X						
Inclusion/Exclusion Criteria	X			X			
Medical History & Demographics	X						
**PHARMACODYNAMICS**							
Serum Phosphorus[5]	X			X			
Serum FGF23[5]	X			X			
Serum ALP[6]				X			
Serum Creatinine[5,6]	X			X			
1,25$(OH)_2D$[5]				X			
2-Hour Urine[5,6]	X			X			
Bone turnover markers[5,7]				X			
**EFFICACY**							
^{99m}Tc Bone Scan[8]					X		
DXA[8]					X		
Standard Radiographs[9]	X						
Xtreme CT of Radius and Tibia[10]	X						
Bone Biopsy[11]						X	
BPI, BFI, SF-36[12]	X				X		

STUDY VISIT	Screening	Baseline[2]					
	Screening Visit	TC1[1]	TC2[1]	Visit 1[3]			
**STUDY WEEK or STUDY DAY**	**Week −4**	**Week −3**	**Week −1**	**Day −3**	**Day −2**	**Day −1**	**Week / Day 0[4]**
HHD, STS, WAL, 6MWT[12]	X				X		
Ambulatory Status				X[19]			
**Pharmacokinetics**							
Serum KRN23							X
**Safety**							
Vital Signs[13]				X	X	X	X
Weight	X				X		
Height	X						
Physical Examination	X			X			
Concomitant Medications	X			X			
Adverse Events	X	X	X	X	X	X	X
Chemistry[14], Hematology[15]	X			X			
Anti-KRN23 Antibody[16]							X
Serum Calcium[4]	X			X			
Serum iPTH[4]	X			X			
25(OH)D	X						
Urinalysis	X			X			
24-hour Urine[17]				X			
ECHO, ECG[8]				X			
Renal Ultrasound[18]	X						

STUDY VISIT	Screening	Baseline[2]					
	Screening Visit	TC1[1]	TC2[1]	Visit 1[3]			
**STUDY WEEK or STUDY DAY**	**Week −4**	**Week −3**	**Week −1**	**Day −3**	**Day −2**	**Day −1**	**Week / Day 0[4]**
Tumor Imaging[20]	X						
Urine Pregnancy Test[21]	X			X			
**DRUG ADMINISTRATION**							
Tetracycline Labeling		X	X				
KRN23[22]							X

[1] TC1 will be a telephone call to the subject within 1 week of the Screening Visit to communicate test results related to eligibility. If eligible for the study with only the FGF23 criterion pending, the subject will be instructed to begin taking a tetracycline (eg, tetracycline HCl and demeclocycline) on Days −20, −19, and −18 prior to the Baseline Visit. TC2 will be a second telephone call to the subject and should be conducted at least 7 days prior to the Baseline Visit to instruct the subject to begin taking a tetracycline on Days −8, −7, and −6 prior to the Baseline Visit.

[2] At the discretion of the Investigator, Baseline Day −3 and Day −2 assessments may be completed in any order to allow for flexibility in scheduling. All assessments from Days −3 and −2 must be completed prior to the bone biopsy, which must be completed on its own day with no other assessments and 1 day prior to dosing on Day 0.

[3] Visit 1 may include overnight stay if deemed necessary by the Investigator. Discharge will occur approximately 24 hours after the first dose of KRN23 but may occur later in cases of prolonged recovery from the bone biopsy procedure

[4] All Day 0 assessments must be performed prior to administration of the first dose of KRN23.

[5] Blood and urine to be collected after a minimum overnight fasting time of 8 hours and prior to drug administration.

[6] Fasting 2-hour urine collections for TmP/GFR, TRP, and urinary calcium. Blood draw for serum creatinine should occur at 1 hour into the urine collection.

[7] Bone turnover markers will include serum measures of CTx, P1NP, BALP, and osteocalcin.

[8] May be completed within ± 5 days of the projected visit date to accommodate scheduling.

[9] At Screening, standard radiographs (skeletal survey) will be obtained of the lateral spine, AP chest, right and left hand/wrist, right and left humerus, right and left radius/ulna, right and left femur/pelvis, right and left tibia/fibula, and right and left foot as well as any location(s) where the subject is currently experiencing tenderness or pain that may reflect underlying pathology, or where the subject has a history of recent (< 3 months) fracture(s).

[10] May be performed any time after the subject has enrolled in the study (after meeting all eligibility criteria) until Baseline.

[11] A bone biopsy of the trans-iliac crest will be performed using general or local anesthesia as per institution practice(s) and performed by a physician trained and experienced in the procedure. The bone biopsy is not required at the Baseline Visit if a previous bone biopsy taken within 12 months of screening confirmed

the diagnosis of osteomalacia, the subjects' clinical manifestations have not changed significantly since the time of the previous biopsy/diagnosis, and the tissue collected at that biopsy is made available for testing for this protocol.

[12] BPI, BFI, SF-36, HHD, STS, WAL, and 6MWT may be completed within ± 5 days of the projected visit date to accommodate scheduling.

[13] Vital sign measurements consist of seated systolic/diastolic blood pressure measured in millimeters of mercury (mm Hg), heart rate (beats per min), respiration rate (breaths per min), and temperature in degrees Celsius (°C). Obtain at the beginning of each visit before any additional assessments are completed and after the subject has rested for 5 minutes.

[14] Serum chemistry panels may include PD parameters (ie, serum phosphorus) and safety parameters of interest (ie, calcium) to avoid duplication of testing. See Table 7.5.6.4.1 for a complete list of Clinical Laboratory Assessments for Safety.

[15] Complete blood count, differential, and platelet count.

[16] If anti-KRN23 antibodies are identified in a given subject, additional samples may be obtained to perform further testing.

[17] 24-hour urine collections for urinary phosphorus, calcium, creatinine, and eGFR; fasting 2-hour urine collections for TmP/GFR, TRP, and urinary calcium/creatinine ratio.

[18] Screening results will be treated as Baseline data.

[19] Ambulatory status at Baseline will be collected retrospectively

[20] Subjects with a TIO diagnosis only. If visible, the tumor will be imaged at Screening using the same radiologic imaging technique (eg, computed tomography [CT] or magnetic resonance imaging [MRI]) used to identify the tumor. In cases where the tumor was initially identified using another imaging technology, a baseline CT scan or MRI (whichever is more clinically appropriate) will be performed. During follow up, the same imaging technique (CT scan or MRI) that was used at Baseline will be utilized to assess the size of the tumor.

[21] For women of childbearing potential only, a serum pregnancy test will be performed in the event of a positive or equivocal urine pregnancy test.

[22] All enrolled subjects will begin treatment with KRN23 at a starting dose of 0.3 mg/kg (Week 0, Day 0, and no sooner than the day after the bone biopsy is performed).

Table 2.2: Schedule of Events – Treatment Period (Weeks 1 – 48)

VISIT TYPE / NUMBER[1]	V1	HH V1	HH V2	V3	HH V4	V5	HH V6	V7	HH V8	V9	HH V10	HH V10.1	HH V11	V12[2]	HH V13	HH V14	V15	HH V16	HH V17	TC3[3]	TC4[3]	V18[4]	
**STUDY WEEK**	**DAY1**	**1**	**2**	**4**	**6**	**8**	**10**	**12**	**14**	**16**	**20**	**21**	**22**	**24**	**28**	**32**	**36**	**40**	**44**	**45**	**47**	**48**	
																						**D1**	**D2**
**PHARMACODYNAMICS**																							
Serum Phosphorus[5]		X	X	X	X	X	X	X	X	X	X	X	X	X	X	X	X	X	X			X	
Serum FGF23[5]	X			X				X		X				X			X					X	
Serum ALP[5]								X		X				X			X					X	
Serum Creatinine[5,6]				X		X		X		X				X			X					X	
1,25(OH)$_2$D[5]		X	X					X		X		X		X			X					X	
2-Hour Urine[5,6]				X		X		X		X				X			X					X	
Bone Biomarkers[5,7]										X				X								X	
**EFFICACY**																							
^{99m}Tc Bone Scan[8]														X								X	
DXA[8]														X								X	
Standard Radiographs[9]								X						X			X					X	
XtremeCT of Radius and Tibia[8]																						X	
Bone Biopsy[10]																							X
BPI, BFI, SF-36[11]								X						X								X	
HHD, STS, WAL, 6MWT[11]								X						X								X	

VISIT TYPE / NUMBER[1]	V1	HH V1	HH V2	V3	HH V4	V5	HH V6	V7	HH V8	V9	HH V10	HH V10.1	HH V11	V12[2]	HH V13	HH V14	V15	HH V16	HH V17	TC3[3]	TC4[3]	V18[4]	
STUDY WEEK	DAY1	1	2	4	6	8	10	12	14	16	20	21	22	24	28	32	36	40	44	45	47	48	
																						D1	D2
**PHARMACOKINETICS**																							
Serum KRN23[12]	X		X	X		X				X	X		X	X		X	X	X				X	
**SAFETY**																							
Vital Signs[13]	X	X	X	X	X	X	X	X	X	X	X	X	X	X	X	X	X	X	X			X	
Weight						X				X				X			X					X	
Physical Examination						X				X				X			X					X	
Concomitant Medications		X	X	X	X	X	X	X	X	X	X	X	X	X	X	X	X	X	X			X	
Adverse Events		X	X	X	X	X	X	X	X	X	X	X	X	X	X	X	X	X	X			X	
Chemistry,[14] Hematology[15]				X		X		X		X	X			X	X	X	X	X	X			X	
Anti-KRN23 Antibody[16]						X				X				X			X					X	
Serum Calcium[5]	X	X	X	X	X	X	X	X	X	X	X	X	X	X	X	X	X	X	X			X	
Serum iPTH[5]	X					X				X				X			X					X	
25(OH)D														X								X	
Urinalysis				X		X				X				X								X	
24-hour Urine[17]						X				X				X			X					X	
ECHO, ECG[8]														X								X	
Renal Ultrasound[8,18]														X								X	
Tumor Imaging[8,19]														X								X	
Urine Pregnancy Test[20]				X		X		X		X	X			X	X	X	X	X	X			X	

VISIT TYPE / NUMBER[1]	V1	HH V1	HH V2	V3	HH V4	V5	HH V6	V7	HH V8	V9	HH V10	HH V10.1	HH V11	V12[2]	HH V13	HH V14	V15	HH V16	HH V17	TC3[3]	TC4[3]	V18[4]	
**STUDY WEEK**	**Day 1**	**1**	**2**	**4**	**6**	**8**	**10**	**12**	**14**	**16**	**20**	**21**	**22**	**24**	**28**	**32**	**36**	**40**	**44**	**45**	**47**	**48**	
																						**D1**	**D2**
**DRUG ADMINISTRATION**																							
Tetracycline Labeling																				X	X		
KRN23[21]				X		X		X		X	X			X	X	X	X	X	X			X	
Dose Adjustment (as needed)				X		X		X		X													

[1] Home health (HH) visits may also be conducted at the clinic depending on proximity of the subject to the investigational site and local availability of home health care services. The visit window is ± 3 days.

[2] The Week 24 visit may be up to 2 days in duration due to the volume of testing; required blood draws may be split across the 2 days.

[3] TC3 will be a telephone call to the subject to instruct the subject to begin taking tetracycline (eg, tetracycline HCl and demeclocycline) on Days, −20, −19, and −18 prior to the Week 48 Day 2 visit. TC4 will be a second telephone call to the subject and should be conducted at least 7 days prior to the Week 48 Day 2 visit to instruct the subject to begin taking a tetracycline on Days −8, −7, and −6 prior to the Week 48 Day 2 visit when the bone biopsy will be performed. Tetracycline will be given to the subject at the clinic during the Week 36 visit.

[4] The Week 48 visit (if applicable) may be up to 2 days in duration due to the volume of testing; required blood draws may be split across the 2 days. The bone biopsy must be completed on its own day with no other assessments.

[5] Blood and urine to be collected after a minimum overnight fasting time of 8 hours and prior to drug administration.

[6] Fasting 2-hour urine collections for TmP/GFR, TRP, and urinary calcium. Blood draw for serum creatinine should occur at 1 hour into the urine collection.

[7] Bone biomarkers will include serum measures of CTx, P1NP, BALP, and osteocalcin.

[8] May be completed within ± 5 days of the projected visit date to accommodate scheduling.

[9] Standard radiographs will be completed in the anatomical location where a fracture or pseudo-fracture is identified at Screening every 12 weeks (at Weeks 24, 36, and 48) until resolution. Unscheduled radiographs will be completed for any new fractures or pseudo-fractures identified during the Treatment Period approximately every 12 weeks from the date of fracture until resolution.

[10] A bone biopsy of the trans-iliac crest will be performed using general or local anesthesia as per institution practice(s) and performed by a physician trained and experienced in the procedure.

[11] BPI, BFI, SF-36, HHD, STS, WAL, and 6MWT may be completed within ± 5 days of the projected visit date to accommodate scheduling.

[12] Analysis of serum KRN23 in samples drawn at Weeks 8, 32, 36, 40, and 48 may be performed retrospectively on available stable stored samples once appropriate approvals and consents are in place.

[13] Vital sign measurements consist of seated systolic/diastolic blood pressure measured in millimeters of mercury (mm Hg), heart rate (beats per min), respiration rate (breaths per min), and temperature in degrees Celsius (°C). Obtain at the beginning of each visit before any additional assessments are completed and after the subject has rested for 5 minutes.

[14] Serum chemistry panels may include PD parameters (ie, serum phosphorus) and safety parameters of interest (ie, calcium) to avoid duplication of testing. See Table 7.5.6.4.1 for a complete list of Clinical Laboratory Assessments for Safety.

[15] Complete blood count, differential, and platelet count.

[16] If anti-KRN23 antibodies are identified in a given subject, additional samples may be obtained to perform further testing.

[17] 24-hour urine collections for urinary phosphorus, calcium, creatinine, and eGFR; fasting 2-hour urine collections for TmP/GFR, TRP, and urinary calcium/creatinine ratio.

[18] Screening results will be treated as Baseline data.

[19] Subjects with a TIO diagnosis only. If visible at Screening, the tumor will be imaged every 24 weeks using the same radiologic imaging technique (eg, CT scan or MRI) used to identify the tumor in order to assess the size of the tumor.

[20] For women of childbearing potential only, a serum pregnancy test will be performed in the event of a positive or equivocal urine pregnancy test.

[21] After the starting dose, doses of KRN23 will be titrated in an effort to achieve a target fasting serum phosphorus range of 2.5 to 4.0 mg/dL. If the subject’s peak serum phosphorus level at Week 2 remains below the target range, the subject’s dose may be increased at Week 4 to 0.6 mg/kg, at the discretion of the Investigator. Doses will then be titrated in increments of 0.2 mg/kg at Weeks 8, 12, and 16 in individual subjects to achieve a target peak serum phosphorus range. If needed, the final dose adjustment increment may be less than the specified increment to reach the 2.0 mg/kg dose. Doses may be titrated at later visits, at the discretion of the Investigator, if there are concerns about safety or suboptimal efficacy, or if a subject undergoes treatment of the underlying tumor.

Table 2.3: Schedule of Events – Treatment Extension Period[1] (Weeks 49 – 120)

VISIT TYPE/NUMBER[2]		HH V19	HH V20	HH V21	HH V22	HH V23	V24	HH V25	HH V26	HH V27	HH V28	HH V29	V30	HH V31	HH V32	HH V33	HH V34	HH V35	V36
**STUDY WEEK**	**Q2W[22]**	**52**	**56**	**60**	**64**	**68**	**72**	**76**	**80**	**84**	**88**	**92**	**96**	**100**	**104**	**108**	**112**	**116**	**120**
**PHARMACODYNAMICS**																			
Serum Phosphorus[4]				X			X			X			X			X			X
Serum FGF23[4]							X						X						X
Serum ALP[4]							X						X						X
Serum Creatinine[4,5]							X						X						X
$1,25(OH)_2D$[4]							X						X						X
2-Hour Urine[4,5]							X						X						X
Bone Biomarkers[4,6]							X						X						X
**EFFICACY**																			
^{99m}Tc Bone Scan[7]													X						
DXA[7]													X						
Standard Radiographs[8]							X						X						X
XtremeCT of Radius and Tibia[7]													X						
BPI, BFI, SF-36[10]							X						X						X
Ambulatory Status[11]							X						X						X
**PHARMACOKINETICS**																			
Serum KRN23							X						X						X
**SAFETY**																			
Vital Signs[12]	X	X	X	X	X	X	X	X	X	X	X	X	X	X	X	X	X	X	X
Weight							X						X						X

VISIT TYPE/NUMBER[2]		HH V19	HH V20	HH V21	HH V22	HH V23	V24	HH V25	HH V26	HH V27	HH V28	HH V29	V30	HH V31	HH V32	HH V33	HH V34	HH V35	V36
**STUDY WEEK**	**Q2W[22]**	**52**	**56**	**60**	**64**	**68**	**72**	**76**	**80**	**84**	**88**	**92**	**96**	**100**	**104**	**108**	**112**	**116**	**120**
Physical Examination							X						X						X
Concomitant Medications	X	X	X	X	X	X	X	X	X	X	X	X	X	X	X	X	X	X	X
Adverse Events	X	X	X	X	X	X	X	X	X	X	X	X	X	X	X	X	X	X	X
Chemistry,[13] Hematology[14]				X			X						X						X
Anti-KRN23 Antibody							X						X						X
Serum Calcium[4]				X			X			X			X			X			X
Serum iPTH[4]				X			X			X			X			X			X
25(OH)D							X						X						X
Urinalysis							X						X						X
24-hour Urine[15]							X						X						X
ECHO, ECG[7]													X						
Renal Ultrasound[7,16]													X						
Tumor Imaging[7,17]							X						X						X
Tumor Identification[18]																			X
PHEX mutational analysis[19]													X						
Urine Pregnancy Test[20]		X	X	X	X	X	X	X	X	X	X	X	X	X	X	X	X	X	X
**DRUG ADMINISTRATION**																			
KRN23[21,22]	X	X	X	X	X	X	X	X	X	X	X	X	X	X	X	X	X	X	X

Footnotes for Table 2.3 follow Table 2.6.

Table 2.4: Schedule of Events – Treatment Extension Period[1] (Weeks 121 – 192)

VISIT TYPE/NUMBER[2]		HH V37	HH V38	HH V39	HH V40	HH V41	V43	HH V44	HH V45	HH V46	HH V47	HH V48	V50	HH V51	HH V52	HH V53	HH V54	HH V55	V57
**STUDY WEEK**	Q2W[22]	124	128	132	136	140	144	148	152	156	160	164	168	172	176	180	184	188	192
**PHARMACODYNAMICS**																			
Serum Phosphorus[4]				X			X			X			X			X			X
Serum FGF23[4]							X						X						X
Serum ALP[4]							X						X						X
Serum Creatinine[4,5]							X						X						X
$1,25(OH)_2D$[4]							X						X						X
2-Hour Urine[4,5]							X						X						X
Bone Biomarkers[4,6]							X						X						X
**EFFICACY**																			
^{99m}Tc Bone Scan[7]							X												
XtremeCT of Radius and Tibia[7]							X												
Skeletal Survey[9]							X												
Targeted radiographs[8]													X						X
BPI, BFI, SF-36[10]							X						X						X
Ambulatory Status[11]							X						X						X
**PHARMACOKINETICS**																			
Serum KRN23							X						X						X
**SAFETY**																			
Vital Signs[12]	X	X	X	X	X	X	X	X	X	X	X	X	X	X	X	X	X	X	X
Weight							X						X						X

VISIT TYPE/NUMBER[2]		HH V37	HH V38	HH V39	HH V40	HH V41	V43	HH V44	HH V45	HH V46	HH V47	HH V48	V50	HH V51	HH V52	HH V53	HH V54	HH V55	V57
**STUDY WEEK**	Q2W[22]	124	128	132	136	140	144	148	152	156	160	164	168	172	176	180	184	188	192
Physical Examination							X						X						X
Concomitant Medications	X	X	X	X	X	X	X	X	X	X	X	X	X	X	X	X	X	X	X
Adverse Events	X	X	X	X	X	X	X	X	X	X	X	X	X	X	X	X	X	X	X
Chemistry,[13] Hematology[14]							X			X			X						X
Anti-KRN23 Antibody							X						X						X
Serum Calcium[4]				X			X			X			X			X			X
Serum iPTH[4]				X			X			X			X			X			X
25(OH)D							X						X						X
Urinalysis							X						X						X
24-hour Urine[15]							X						X						X
ECHO, ECG[7]							X												X
Renal Ultrasound[7,16]							X												X
Tumor Imaging[7,17]							X												X
Tumor Identification[18]													X						
Urine Pregnancy Test[20]		X	X	X	X	X	X	X	X	X	X	X	X	X	X	X	X	X	X
**DRUG ADMINISTRATION**																			
KRN23[21,22]	X	X	X	X	X	X	X	X	X	X	X	X	X	X	X	X	X	X	X

Footnotes for Table 2.4 follow Table 2.6.

Table 2.5: Schedule of Events – Treatment Extension Period[1] (Weeks 193 – 244)

VISIT TYPE/NUMBER[2]		HH V58	HH V59	HH V60	HH V61	HH V62	V64	HH V65	HH V66	HH V67	HH V68	HH V69	V70	HH V71
**STUDY WEEK**	**HH Q2W[22]**	**196**	**200**	**204**	**208**	**212**	**216**	**220**	**224**	**228**	**232**	**236**	**240**	**244**
**PHARMACODYNAMICS**														
Serum Phosphorus[4]				X			X			X			X	
Serum FGF23[4]							X						X	
Serum ALP[4]							X						X	
Serum Creatinine[4,5]							X						X	
1,25(OH)$_2$D[4]							X						X	
2-Hour Urine[4,5]							X						X	
Bone Biomarkers[4,6]							X						X	
**EFFICACY**														
Targeted radiographs[8]							X						X	
BPI, BFI, SF-36[10]							X						X	
Ambulatory Status[11]							X						X	
**PHARMACOKINETICS**														
Serum KRN23							X						X	
**SAFETY**														
Vital Signs[12]	X	X	X	X	X	X	X	X	X	X	X	X	X	X
Weight							X						X	
Physical Examination							X						X	
Concomitant Medications	X	X	X	X	X	X	X	X	X	X	X	X	X	X
Adverse Events	X	X	X	X	X	X	X	X	X	X	X	X	X	X
Chemistry,[13] Hematology[14]							X			X			X	

VISIT TYPE/NUMBER[2]		HH V58	HH V59	HH V60	HH V61	HH V62	V64	HH V65	HH V66	HH V67	HH V68	HH V69	V70	HH V71
STUDY WEEK	HH Q2W[22]	196	200	204	208	212	216	220	224	228	232	236	240	244
Anti-KRN23 Antibody							X						X	
Serum Calcium[4]				X			X			X			X	
Serum iPTH[4]				X			X			X			X	
25(OH)D							X						X	
Urinalysis							X						X	
24-hour Urine[15]							X						X	
ECHO, ECG[7]							X						X	
Renal Ultrasound[7,16]							X						X	
Tumor Imaging[7,17]													X	
Tumor Identification[18]							X							
Urine Pregnancy Test[20]		X	X	X	X	X	X	X	X	X	X	X	X	X
**DRUG ADMINISTRATION**														
KRN23[21,22]	X	X	X	X	X	X	X	X	X	X	X	X	X	X

Footnotes for Table 2.5 follow Table 2.6.

Table 2.6: Schedule of Events – Treatment Extension Period[1] (Weeks 248 – End of Study) and Safety Follow-up

VISIT TYPE/NUMBER[2]		HH V72	HH V73	HH V74	HH V75	V76	HH V77	HH V78	HH V79	HH V80	HH V81	V82	HH V83	HH V84	V85	Safety Follow-up TC[23]	Safety Follow-up TC[24]
STUDY WEEK	HH Q2W[22]	248	252	256	260	264	268	272	276	280	284	288	292	296	W300/ EOS/ET[3]	6 Weeks From Last Dose	12 Weeks From Last Dose
**PHARMACODYNAMICS**																	
Serum Phosphorus[4]			X			X			X			X			X		
Serum FGF23[4]						X						X			X		
Serum ALP[4]						X						X			X		
Serum Creatinine[4,5]						X						X			X		
$1,25(OH)_2D$[4]						X						X			X		
2-Hour Urine[4,5]						X						X			X		
Bone Biomarkers[4,6]						X						X			X		
**EFFICACY**																	
^{99m}Tc Bone Scan[7]															X		
BPI, BFI, SF-36[10]						X						X			X		
Ambulatory Status[11]						X						X			X		
**PHARMACOKINETICS**																	
Serum KRN23						X						X			X		
**SAFETY**																	
Vital Signs[12]	X	X	X	X	X	X	X	X	X	X	X	X	X	X	X		
Weight						X						X			X		
Physical Examination						X						X			X		
Concomitant Medications	X	X	X	X	X	X	X	X	X	X	X	X	X	X	X	X	X

VISIT TYPE/NUMBER[2]		HH V72	HH V73	HH V74	HH V75	V76	HH V77	HH V78	HH V79	HH V80	HH V81	V82	HH V83	HH V84	V85	Safety Follow-up TC[23]	Safety Follow-up TC[24]
**STUDY WEEK**	**HH Q2W[22]**	**248**	**252**	**256**	**260**	**264**	**268**	**272**	**276**	**280**	**284**	**288**	**292**	**296**	**W300/ EOS/ET[3]**	**6 Weeks From Last Dose**	**12 Weeks From Last Dose**
Adverse Events	X	X	X	X	X	X	X	X	X	X	X	X	X	X	X	X	X
Chemistry,[13] Hematology[14]						X			X			X			X		
Anti-KRN23 Antibody						X						X			X		
Serum Calcium[4]			X			X			X			X			X		
Serum iPTH[4]			X			X			X			X			X		
25(OH)D						X						X			X		
Urinalysis						X						X			X		
24-hour Urine[15]						X						X			X		
ECHO, ECG[7]						X						X			X		
Renal Ultrasound[7],[16]						X						X			X		
Tumor Imaging[7],[17]												X			X		
Tumor Identification[18]						X									X		
Urine Pregnancy Test[20]		X	X	X	X	X	X	X	X	X	X	X	X	X	X		
**DRUG ADMINISTRATION**																	
KRN23[21,22]	X	X	X	X	X	X	X	X	X	X	X	X	X	X			

[1] During the Treatment Extension Period (Weeks 49 – 300), clinic visits will occur at approximately 24-week intervals.

[2] Home health (HH) visits may also be conducted at the clinic depending on proximity of the subject to the investigational site and local availability of home health care services. The visit window is ± 3 days.

[3] Upon regulatory approval and commercial availability of KRN23 for the treatment of TIO, subjects will have their End of Study (EOS) Visit, and transition to prescription drug, as recommended by their treating physician. Until commercial availability, subjects may continue treatment with KRN23 for up to 252 weeks in the Treatment Extension Period, or until 31 January 2021, whichever is sooner, and then undergo their EOS Visit. Subjects will undergo an

Early Termination (ET) Visit if the subject withdraws consent and discontinues from the study, the subject is discontinued from the study at the discretion of the Investigator or Ultragenyx, or the study is terminated. The EOS Visit or ET Visit may be up to 2 days in duration due to the volume of testing; required blood draws may be split across 2 days. ECHO will not be performed at the EOS or ET Visit if the assessment was conducted within 3 months of termination. ^{99m}TC bone scan will not be performed at the EOS or ET visit if the assessment was performed within 12 months of termination. BPI, BFI, SF-36, anti-KRN23 antibody assessment, and serum iPTH will not be performed at the EOS or ET Visit if they were conducted within 4 weeks of termination.

[4] Blood and urine to be collected after a minimum overnight fasting time of 8 hours and prior to drug administration.

[5] Fasting 2-hour urine collections for TmP/GFR, TRP, and urinary calcium. Blood draw for serum creatinine should occur at 1 hour into the urine collection.

[6] Bone biomarkers will include serum measures of CTx, P1NP, BALP, and osteocalcin.

[7] May be completed within ± 5 days of the projected visit date to accommodate scheduling.

[8] Targeted radiographs will be completed in the anatomical location where a fracture or pseudo-fracture was identified at Screening every 24 weeks starting at Week 72, until resolution or up to Week 240. The skeletal survey at Week 144 will be used to capture the 24-week follow-up targeted radiographs for identified fractures or pseudo-fractures.

[9] Skeletal survey includes standard radiographs of the chest, lateral spine, right and left hand/wrist, right and left humerus, right and left radius/ulna, right and left femur/pelvis, right and left tibia/fibula, and right and left foot

[10] BPI, BFI, and SF-36 may be completed within ± 5 days of the projected visit date to accommodate scheduling.

[11] Ambulatory assessment will be performed for each subject at the first site visit after proper approvals and consents are obtained and at all site visits thereafter.

[12] Vital sign measurements consist of seated systolic/diastolic blood pressure measured in millimeters of mercury (mm Hg), heart rate (beats per min), respiration rate (breaths per min), and temperature in degrees Celsius (°C). Obtain at the beginning of each visit before any additional assessments are completed and after the subject has rested for 5 minutes.

[13] Serum chemistry panels may include PD parameters (ie, serum phosphorus) and safety parameters of interest (ie, calcium) to avoid duplication of testing. See Table 7.5.6.4.1 for a complete list of Clinical Laboratory Assessments for Safety.

[14] Complete blood count, differential, and platelet count.

[15] 24-hour urine collections for urinary phosphorus, calcium, creatinine, and eGFR; fasting 2-hour urine collections for TmP/GFR, TRP, and urinary calcium/creatinine ratio.

[16] Screening results will be treated as Baseline data.

[17] Subjects with a TIO diagnosis only. If visible at Screening, the tumor will be imaged every 24 weeks through Week 144 and then every 48 weeks for the remainder of the study using the same radiologic imaging technique (eg, CT scan or MRI) used to identify the tumor in order to assess the size of the tumor. For subjects in whom the tumor is identified during the study by ^{68}Ga-DOTATATE positron emission tomography (PET)/computed tomography (CT) or another method, tumor imaging (CT or MRI, at the discretion of the Investigator) should be performed within 21 (± 7) days of identification to assess tumor size, at an unscheduled visit if necessary, and thereafter every 48 weeks per the Schedule of Events. If the MRI/CT imaging following tumor identification

has occurred within 3 months of the next scheduled MRI/CT assessment, the scheduled assessment will be skipped. Tumor imaging will not be performed at the EOS/ET Visit if it has been performed within the past 12 months.

[18] To be performed only in subjects with TIO for whom the tumor was not identified at Baseline or for subjects who have a clinical indication of a possible new tumor every 48 weeks (or in alignment with regularly scheduled clinic visits) after Week 120. The preferred imaging technique is ^{68}Ga-DOTATATE PET/CT. Octreotide-SPECT (SPECT/CT) or FDG-PT/CT may be used if ^{68}Ga-DOTATATE PET/CT is not available. (If none of these methods are available, another imaging technique may be used at Investigator discretion.) Tumor identification will not be performed at the EOS or ET visit if it has been performed within the past 12 months.

[19] A blood sample for *PHEX* mutational analysis will be collected at Week 96 or the next appropriate visit after appropriate approvals and consents have been obtained for any subject who experienced TIO symptoms beginning in childhood and for whom a causative tumor has not been identified. A *PHEX* mutational analysis previously performed by a qualified laboratory as part of routine medical care may be used if available.

[20] For women of childbearing potential only, a serum pregnancy test will be performed in the event of a positive or equivocal urine pregnancy test.

[21] After the starting dose, doses of KRN23 will be titrated in an effort to achieve a target fasting serum phosphorus range of 2.5 to 4.0 mg/dL (see Table 7.4.5.1). If the subject's peak serum phosphorus level at Week 2 remains below the target range, the subject's dose may be increased at Week 4 to 0.6 mg/kg, at the discretion of the Investigator. Doses will then be titrated in increments of 0.2 mg/kg at Weeks 8, 12, and 16 in individual subjects to achieve a target peak serum phosphorus range. If needed, the final dose adjustment increment may be less than the specified increment to reach the 2.0 mg/kg dose. Doses may be titrated at later visits, at the discretion of the Investigator, if there are concerns about safety or suboptimal efficacy, or if a subject undergoes treatment of the underlying tumor (see Table 7.4.5.2 and Table 7.4.5.3).

[22] Dosing frequency may be increased to Q2W dosing if the subject reaches a dose of 2.0 mg/kg Q4W and serum phosphorus levels remain below the lower limit of normal on 2 consecutive trough measurements (see Table 7.4.5.2). Additional HH visits will be scheduled for Q2W dose administration if necessary. Vital signs, adverse events, and concomitant medications will also be collected at these additional HH visits.

[23] The site personnel will initiate a Safety Follow-up telephone call (TC) approximately 6 weeks (+ 5 days) after the last dose of study drug to collect information on any ongoing or new AEs, SAEs, or concomitant medications. An additional Safety Follow-up TC will occur at 12 weeks (+ 5 days) after the last dose of study drug (see footnote 24). If subjects have started receiving KRN23 (burosumab) via an alternative mechanism (eg, commercial treatment) by the time of the 6-week Safety Follow-up TC, the 12-week Safety Follow-up TC may not be necessary.

[24] Only for subjects not continuing KRN23 (burosumab) therapy under commercial use or another mechanism at the time of the 6-week Safety Follow-up TC. The site personnel will initiate a Safety Follow-up TC at 12 weeks (+ 5 days) after the last dose of study drug to collect information on any ongoing or new AEs, SAEs, or concomitant medications.

3 TABLE OF CONTENTS

1 TITLE PAGE 1

2 SYNOPSIS..5

3 TABLE OF CONTENTS...34

4 LIST OF ABBREVIATIONS AND DEFINITION OF TERMS 39

5 INTRODUCTION 42

5.1 Study Rationale 42

5.2 Overview of the Disease 43

5.3 Brief Overview of the Development of the Product 44

5.3.1 Brief Description of the Investigational Product...44

5.3.2 Nonclinical Studies..45

5.3.3 Previous and Ongoing Clinical Studies...46

5.4 Summary of Overall Risks and Potential Benefits...50

6 STUDY OBJECTIVES...51

7 INVESTIGATIONAL PLAN...53

7.1 Overall Study Design and Plan 53

7.2 Discussion of Study Design, Including Choice of Control Group.........................55

7.3 Selection of Study Population..55

7.3.1 Inclusion Criteria..55

7.3.2 Exclusion Criteria...56

7.3.3 Removal of Subjects from Therapy or Assessment 57

7.3.3.1 Stopping Rules 58

7.4 Treatments..58

7.4.1 Treatments Administered 58

7.4.2 Identity of Investigational Product(s)..59

7.4.2.1 Investigational Test Product 59

7.4.2.2 Placebo or Active Comparator...59

7.4.3 Method of Assigning Subjects to Treatment Groups 59
7.4.4 Selection of Doses in the Study 59
7.4.5 Selection and Timing of Dose for Each Subject 60
7.4.5.1 Dosing in Subjects Undergoing Treatment of Underlying Tumor 63
7.4.6 Blinding 64
7.4.7 Prior and Concomitant Therapy 64
7.4.7.1 Prohibited Medications 64
7.4.7.2 Permitted Medications 65
7.4.8 Treatment Compliance 65
7.5 Study Procedures and Assessments 65
7.5.1 Schedule of Events 66
7.5.2 Primary Efficacy Assessments 66
7.5.3 Additional Pharmacodynamic Measurements 67
7.5.4 Clinical Assessments 67
7.5.4.1 Clinical Outcomes Measurements 68
7.5.4.2 Patient Reported Outcomes Measurements 69
7.5.4.3 Bone Health Measurements 69
7.5.4.4 Ambulatory Assessment 70
7.5.5 Pharmacokinetic Measurements 70
7.5.6 Safety Measurements 70
7.5.6.1 Medical History 70
7.5.6.2 Concomitant Medications/Therapies 71
7.5.6.3 Adverse Events 71
7.5.6.4 Clinical Laboratory Tests for Safety 72
7.5.6.5 Anti-KRN23 Antibody Screening 73
7.5.6.6 Renal Ultrasound and Glomerular Filtration Rate 74
7.5.6.7 Echocardiogram 74

7.5.6.8 Electrocardiogram 74
7.5.6.9 Tumor Imaging 74
7.5.6.10 Vital Signs and Weight 75
7.5.6.11 Physical Examination....75
7.5.6.12 Pregnancy Testing and Contraception 76
7.5.6.13 Dose Limiting Toxicity....77
7.5.7 Appropriateness of Measurements 77
7.6 Statistical Methods and Determination of Sample Size....78
7.6.1 Efficacy Endpoints 79
7.6.1.1 Primary Efficacy Endpoints....79
7.6.1.2 Secondary Endpoints 79
7.6.1.3 Exploratory Endpoints 80
7.6.2 Statistical and Analytical Plans 80
7.6.2.1 Analysis Populations....80
7.6.2.2 Statistical Principles....80
7.6.2.3 Demography, Baseline Characteristics, and Disposition....80
7.6.2.4 Efficacy Analyses 81
7.6.2.5 Safety Analyses....82
7.6.2.6 Exposure 83
7.6.2.7 Anti-KRN23 Antibody Assessment....83
7.6.3 Determination of Sample Size....83
8 STUDY CONDUCT....84
8.1 Ethics....84
8.1.1 Institutional Review Board....84
8.1.2 Ethical Conduct of Study 84
8.1.3 Subject Information and Consent 84
8.2 Investigators and Study Administrative Structure 85

8.3 Investigational Product Accountability....85
8.4 Data Handling and Record Keeping....86
8.4.1 Case Report Forms and Source Documents....86
8.4.2 Data Quality Assurance....86
8.4.3 Record Retention....87
8.5 Reporting and Follow-up of Adverse Events....87
8.5.1 Definition of Adverse Events....87
8.5.2 Severity of Adverse Events....88
8.5.3 Relationship of Adverse Events to Study Drug....89
8.5.4 Adverse Event Reporting to Ultragenyx....90
8.5.4.1 General....90
8.5.4.2 Serious Adverse Events, Serious Adverse Drug Reactions, and Requirements for Immediate Reporting....90
8.5.4.3 Urgent Safety Reporting....91
8.5.4.4 Adverse Drug Reaction Reporting....91
8.5.4.5 Pregnancy in Subject or Partner....92
8.5.4.6 Safety Contact Information....92
8.6 Financing and Insurance....92
8.7 Publication Policy....92
9 REFERENCES....93
10 SIGNATURE PAGE....95

LIST OF TABLES

Table 2.1: Schedule of Events – Screening and Baseline....16
Table 2.2: Schedule of Events – Treatment Period (Weeks 1 – 48)....20
Table 2.3: Schedule of Events – Treatment Extension Period[1] (Weeks 49 – 120)....24

Table 2.4: Schedule of Events – Treatment Extension Period[1] (Weeks 121 – 192) 26

Table 2.5: Schedule of Events – Treatment Extension Period[1] (Weeks 193 – 244) 28

Table 2.6: Schedule of Events – Treatment Extension Period[1] (Weeks 248 – End of Study) and Safety Follow-up 30

Table 5.3.3.1: Summary of Clinical Studies with KRN23....47

Table 7.4.5.1: KRN23 Initial Dose Titration Scheme....61

Table 7.4.5.2: KRN23 Dose Titration Scheme for Dose Adjustments and Increases in Dose Frequency from Q4W to Q2W At or After Week 96 62

Table 7.4.5.1.1: KRN23 Dose Titration Scheme Following Treatment of an Underlying Tumor 63

Table 7.5.6.4.1: Clinical Laboratory Assessments....73

4 LIST OF ABBREVIATIONS AND DEFINITION OF TERMS

Abbreviations

1,25(OH)$_2$D	1,25-dihydroxyvitamin D
25(OH)D	25-hydroxyvitamin D
6MWT	6-minute walk test
ADA	Anti-drug antibody
AE	Adverse event
ALP	Alkaline phosphatase
BALP	Bone-specific alkaline phosphatase
BFI	Brief fatigue inventory
BPI	Brief pain inventory
BS	Bone surface
BV	Bone volume
CFR	Code of Federal Regulations
CRF	Case Report Form
CT	Computed tomography
CTCAE	Common Terminology Criteria for Adverse Events
CTx	Carboxy terminal cross-linked telopeptide of type 1 collagen
DLT	Dose-limiting toxicity
DXA	Dual-energy X-ray absorptiometry
ECG	Electrocardiogram
ECHO	Echocardiogram
EDC	Electronic Data Capture
eGFR	Estimated glomerular filtration rate
ENS	Epidermal nevus syndrome
EOS	End of study
ET	Early Termination
EudraCT	European Union Drug Regulating Authorities Clinical Trials
FDA	Food and Drug Administration
FGF23	Fibroblast growth factor 23
GCP	Good Clinical Practice
GFR	Glomerular filtration rate
HCl	Hydrochloride
HH	Home Health
HHD	Hand-held dynamometry
HIPAA	Health Insurance Portability and Accountability Act
ICF	Informed Consent Form
ICH	International Conference on Harmonisation of Technical Requirements for Registration of Pharmaceuticals for Human Use
IgG1	Immunoglobulin G1
IND	Investigational New Drug (Application)
iPTH	Intact parathyroid hormone
IRB	Institutional Review Board

IV	Intravenous(ly)
KHK	Kyowa Hakko Kirin Pharma, Inc. (the Sponsor’s development partner for KRN23)
KRN23	Investigational product, an anti-FGF23 antibody
LLN	Lower limit of normal
LVH	Left ventricular hypertrophy
mAb	Monoclonal antibody
MedDRA	Medical Dictionary for Regulatory Activities
MLt	Mineralization lag time
MRI	Magnetic resonance imaging
NOAEL	No adverse effect level
OS	Osteoid surface
O.Th	Osteoid thickness
OV	Osteoid volume
P1NP	Procollagen type 1 N-propeptide
PD	Pharmacodynamics
PET	Positron emission tomography
PHEX	Phosphate-regulating gene with Homology to Endopeptidases located on the X chromosome
PK	Pharmacokinetics
PMT	Phosphaturic mesenchymal tumor
PT	Preferred Term
PTH	Parathyroid hormone
QTc	Corrected QT interval
Q2W	Every 2 weeks
Q4W	Every 4 weeks
RLS	Restless legs syndrome
SAE	Serious adverse event
SAP	Statistical Analysis Plan
SC	Subcutaneous(ly)
SF-36	36-item short form health survey
STS	Sit-to-stand (test)
SUSAR	Suspected unexpected serious adverse reaction
TC	Telephone call
TIO	Tumor-induced osteomalacia (also known as oncogenic osteomalacia)
TmP/GFR	Ratio of renal tubular maximum phosphate reabsorption rate to glomerular filtration rate
TRP	Tubular reabsorption of phosphate
USA	United States of America
WAL	Weighted arm lift (test)
XLH	X-linked hypophosphatemia

Definition of Terms

°C	Degrees Celsius
dL	Deciliter
g	Gram
kDa	Kilodalton
kg	Kilogram
L	Liter
mg	Milligram
mL	Milliliter
mmol	Millimole

5 INTRODUCTION

5.1 Study Rationale

KRN23 is a fully human immunoglobulin G1 (IgG1) monoclonal antibody (mAb) that binds to and inhibits the activity of fibroblast growth factor 23 (FGF23), leading to an increase in serum phosphorus levels. There are multiple disorders (each with a unique underlying cause) that result in unusually high circulating levels of FGF23, which in turn results in renal phosphate wasting and reduced (or aberrantly normal in relationship to the elevated FGF23 levels) levels of 1,25-dihydroxy vitamin D (1,25[OH]$_2$D). Across these disorders the clinical symptoms are similar and often include osteomalacia (and, in children, rickets), muscle weakness, fatigue, bone pain, and fractures. KRN23 has been studied in 1 of these disorders, X-linked hypophosphatemia (XLH), in which a mutation in the Phosphate-regulating gene with Homology to Endopeptidases located on the X chromosome (*PHEX*; a phosphate-regulating gene with highest expression in bone cells) leads to elevated levels of FGF23. In the Hyp mouse model, a relevant murine model of XLH, treatment with a mouse anti-FGF23 mAb normalized or ameliorated many of the characteristic abnormalities associated with XLH (Aono et al. 2009; Aono et al. 2011). In single- and repeat-dose clinical studies in subjects with XLH, subcutaneous (SC) administration of KRN23 consistently increased and sustained serum phosphorus levels and tubular reabsorption of phosphate (TRP) without a major impact on urine calcium levels or vitamin D metabolism. KRN23 (burosumab) was approved by the Food and Drug Administration (FDA) on 17 April 2018 for the treatment of XLH in adult and pediatric patients 1 year of age and older, and a positive European Commission conditional marketing authorization was received on 23 February 2018 for the treatment of XLH with radiographic evidence of bone disease in children 1 year of age and older and adolescents with growing skeletons.

Similar to XLH, tumor-induced osteomalacia (TIO), also known as oncogenic osteomalacia, is an acquired condition in which tumors, whose pathology is usually phosphaturic mesenchymal tumors (PMT), cause excessive ectopic production of FGF23 that leads to renal phosphate wasting and impaired vitamin D synthesis. The high levels of FGF23 result in a similar biochemical profile to XLH that includes low serum phosphorus, phosphaturia, an abnormally low ratio of renal tubular maximum phosphate reabsorption rate to glomerular filtration rate (TmP/GFR), elevated alkaline phosphatase (ALP), normal calcium, and low to normal levels of 1,25(OH)$_2$D. In TIO, tumors that are typically benign cause excessive ectopic production of FGF23, and if the causal tumor can be completely removed, the syndrome remits. In patients for whom the tumor is inoperable, the current standard of care consists of oral phosphate and/or vitamin D replacement. Efficacy of this treatment is often limited; it does not treat the underlying disease; and its benefits must be balanced with monitoring for potential risks such as nephrocalcinosis, hypercalciuria, and hyperparathyroidism. Epidermal nevus syndrome (ENS) are rare congenital syndromes characterized by the presence of epidermal nevi in association with 1 or more other developmental abnormalities of other organ systems including the nervous, skeletal, cardiovascular, and ocular systems. Hypophosphatemia may rarely be 1 of the skeletal manifestations of ENS presenting in children primarily as rickets and in adults as

osteomalacia. In all ENS-associated osteomalacia (also known as cutaneous skeletal hypophosphatemia syndrome) cases in which serum FGF23 levels were assessed, they were found to be elevated (Lim et al. 2014). The source of the excess FGF23 in ENS-associated osteomalacia is unclear. Although some previous studies had suggested that the source of excess FGF23 was from skin lesions, more recent studies have failed to detect FGF23 protein or FGF23 mRNA from samples of skin lesions. Thus, it is possible that an as-yet unknown substance produced from the skin may induce FGF23 excess or that FGF23 excess in this syndrome may be from dysplastic bone or other skeletal lesions that are often part of the constellation of congenital malformations associated with the underlying somatic mosaic mutation that forms the basis of ENS-associated osteomalacia. Compared with TIO, complete resection and surgical cure leading to removal of the source of excess FGF23 is less likely.

Treatment with oral phosphate and calcitriol as well as excision of these skin lesions have produced mixed reports of efficacy (Olivares et al. 1999). In many cases, improvement, if any, is modest and transient.

This is a Phase 2 study designed to assess the efficacy, safety, pharmacokinetics (PK), and pharmacodynamics (PD) of KRN23 in subjects with TIO whose tumor is inoperable (defined by an inability to localize the tumor or the impractical nature of surgery due to the extent of disease or the location of the tumor) or ENS-associated osteomalacia, where skin lesion removal therapy is not considered a valid treatment option. It is hypothesized that KRN23 may provide clinical benefit in this patient population because of the common underlying feature: abnormally elevated FGF23 levels.

5.2 Overview of the Disease

TIO, also known as oncogenic osteomalacia, is an acquired condition in which typically benign tumors cause excessive ectopic production of FGF23 that leads to renal phosphate wasting and impaired calcitriol synthesis. The high levels of FGF23 result in a similar biochemical profile to XLH that includes low serum phosphorus, phosphaturia, an abnormally TmP/GFR, elevated ALP, normal calcium, and low to normal levels of 1,25$(OH)_2$D (Drezner 1999). However, because the FGF23 levels observed in TIO far exceed those seen in XLH, the clinical syndrome resulting from the renal phosphate wasting is often more severe and characterized by poor bone mineralization, fractures, and muscle and bone pain. The tumors characteristic of TIO are typically small, slow-growing masses that may be located in various areas, including long bones, soft tissues, nasal sinuses, and the groin. Most tumors are benign, phosphaturic mesenchymal connective tissue tumors, also known as PMTs, although a few cases of malignant tumors have been reported (Folpe et al. 2004; Weidner et al. 1987). TIO-related tumors can particularly difficult to find, even with the use of high-resolution imaging techniques (Jan de Beur et al. 2002). When the tumor can be identified and surgically removed, FGF23 levels decline dramatically and the clinical syndrome remits within days to weeks. If the tumor cannot be located, or if the tumor resides in an area that precludes surgical excision because of the risk of damage to neighboring structures, conventional therapy is the same as with XLH, ie, phosphorus supplementation in

combination with calcitriol. In some cases, calcitriol alone is administered (Geller et al. 2007). The efficacy of this treatment is limited given the magnitude of the FGF23 levels and the severity of the resulting phosphate wasting. Its benefits should be weighed against the need for frequent monitoring to mitigate the risks of nephrocalcinosis, hypercalciuria, and hyperparathyroidism.

Epidermal nevus syndromes are rare congenital syndromes, characterized by the presence of epidermal nevi in association with 1 or more other developmental abnormalities of other organ systems including the nervous, skeletal, cardiovascular, and ocular systems (Stavrianeas et al. 2004). The epidermal nevi are non-neoplastic overgrowth of various components of skin cells (eg, sebaceous cells- linear sebaceous nevus) that are usually distributed linearly along the lines of Blaschko, corresponding to the movement of skin cells during embryogenesis. While these epidermal nevi may be limited to the skin, the additional systemic abnormalities almost exclusively occur in the setting of extensive skin surface area involvement (10 – 60% of the body surface area) consistent with early embryonic somatic mutation in multipotent progenitor cells. Specific genetic defects and timing of mutation during fetal development influence the varied phenotypes of ENS (Lim et al. 2014). Hypophosphatemia may rarely be 1 of the skeletal manifestations of ENS presenting in children primarily as rickets and in adults as osteomalacia. In all ENS-associated osteomalacia cases in which serum FGF23 levels were assessed, they were found to be elevated (Lim et al. 2014). Standard of care with oral phosphate and calcitriol as well as excision of these skin lesions have produced mixed reports of efficacy (Olivares et al. 1999). In many cases, improvement, if any, is modest and transient.

5.3 Brief Overview of the Development of the Product

A brief overview of existing information on KRN23 is provided here. Comprehensive information on KRN23 is contained in the Investigator’s Brochure provided by Ultragenyx, which should be reviewed prior to initiating the study.

5.3.1 Brief Description of the Investigational Product

KRN23 is a fully human IgG_1 mAb that binds to and inhibits the activity of FGF23. KRN23 is expressed in Chinese hamster ovary dihydrofolate reductase deficient cells. The secreted KRN23 antibody is recovered from the culture medium and purified using a series of chromatographic and filtration steps. Based on the amino acid sequence, the predicted molecular mass of KRN23 is approximately 140 kDa.

FGF23 reduces serum phosphorus levels by 2 distinct mechanisms of action (Fukumoto 2008; Razzaque et al. 2007; Yamazaki et al. 2008). The primary mechanism is to inhibit phosphate reabsorption in the proximal tubule of the kidney. The secondary mechanism is to decrease phosphate absorption by the small intestine through the inhibition of $1,25(OH)_2D$ production in the kidney.

KRN23 has the potential to block or reduce FGF23 action and improve phosphate metabolism in patients with TIO or ENS-associated osteomalacia. KRN23 binds the amino-terminal domain of FGF23 that interacts with the FGFR1-binding portion of the combination FGFR1/Klotho receptor, preventing FGF23 from binding and signaling its receptor. Both intact and fragmented FGF23 polypeptides are immunoprecipitated with KRN23 (Yamazaki et al. 2008). By inhibiting FGF23, KRN23 restores the ratio of the tubular maximum reabsorption rate of phosphate to glomerular filtration rate (TmP/GFR) in the kidney and increases the production of 1,25$(OH)_2$D, which also enhances intestinal absorption of phosphate. The action on both kidney reabsorption and intestinal absorption, improves serum phosphorus levels, which is expected to improve bone mineralization and reduce the diverse bone and non-bone manifestations associated with hypophosphatemia in patients with TIO or ENS-associated osteomalacia.

5.3.2 Nonclinical Studies

The Sponsor’s development partner, Kyowa Hakko Kirin Pharma, Inc. (KHK), has conducted a series of nonclinical pharmacology, PK, and toxicology studies in rabbits and cynomolgus monkeys to support the use of KRN23 in clinical studies. Findings of potential clinical significance and relevance to this protocol are summarized below; additional information is provided in the Investigator’s Brochure.

- KRN23 binds to human, rabbit, and monkey FGF23 with comparable affinities. In a Good Laboratory Practice study, KRN23 cross-reactivity was evaluated against a full panel of human, rabbit (32 tissues), and cynomolgus monkey (33 tissues) tissues by immunohistochemistry. No specific KRN23 staining was observed, thus no untoward direct-effects of KRN23 are expected in any tissues of normal humans, rabbits, or cynomolgus monkeys
- In a 40-week toxicity study in male and female adult cynomolgus monkeys, KRN23 was administered every 2 weeks at doses of 0 (control), 0.03, 0.3, 3, and 30 mg/kg intravenously (IV) and 30 mg/kg SC. The main observed histopathological change was metastatic mineralization of various soft tissues, including the kidney, eyeball, lung, manus, skeletal muscle, and submandibular gland in the 3.0 mg/kg IV and 30 mg/kg SC and IV groups. Mineralization in the kidney and eyeball did not show obvious reversibility after a 13-week recovery period. The no adverse effect level (NOAEL) was 0.03 mg/kg for males and 0.3 mg/kg (IV) for females based on the histopathologic finding metastatic mineralization; in males mineralization and hyperstosis of cortical bone were more severe in males than females. The NOAEL in a 40-week toxicity study in juvenile cynomolgus monkeys and a single-dose study in rabbits was 0.3 mg/kg KRN23 IV
- Prolonged and excessive elevation of serum phosphate beyond normal levels was associated with soft tissue and organ mineralization including the kidney where nephrocalcinosis was observed at the highest doses tested (IV and SC); reversibility of mineralization could not be established

- The most prominent pharmacologic actions of KRN23 were dose-dependent changes in serum inorganic phosphorus and 1,25$(OH)_2$D in rabbits and in juvenile, adult, and pregnant cynomolgus monkeys
- No gross or histopathological abnormalities were observed at the IV infusion sites or SC injection sites in the 40-week repeat dose toxicity studies in adult and juvenile cynomolgus monkeys
- KRN23 demonstrated consistent and predictable PK behavior in both rabbits and cynomolgus monkeys based on the results of single- and repeat-dose studies where exposure was by either the IV or SC route of administration
- The NOAEL was the same in juvenile and adult monkeys, suggesting no difference in sensitivity to the adverse effects of KRN23. The results from single- and repeat-dose toxicology studies in rabbits and in juvenile, adult, and pregnant cynomolgus monkeys suggest that the primary toxicological effects of KRN23 are associated with prolonged and excessive antagonism of the normal regulatory actions of FGF23 on renal TRP and vitamin D metabolism

5.3.3 Previous and Ongoing Clinical Studies

Table 5.3.3.1 summarizes previous and ongoing clinical studies of KRN23. Details of study parameters and PK, PD, clinical efficacy, and safety results where available are provided in the Investigator’s Brochure.

Table 5.3.3.1: Summary of Clinical Studies with KRN23

Study Number	Study Design	Number of Subjects	Dosage and Regimen	Key Endpoints	Study Status
**XLH in Adults and Children**					
**KRN23-US-02**	Phase 1, Double-blind, Randomized, Placebo-controlled Single-dose, Dose-escalation Study	Adults<br>38 enrolled<br>38 dosed<br>38 completed	Single-dose KRN23<br>IV: 0, 0.003, 0.01, 0.03, 0.1, and 0.3 mg/kg (n=17)<br>SC: 0, 0.1, 0.3, 0.6 and 1.0 mg/kg (n=12)<br>Placebo: (n=9)	- Safety of single-dose administered IV or SC<br>- PK profile, PD effect, hypersensitivity	Completed
**KRN23-INT-001**	Phase 1/2, Open-label, Repeat-dose, Dose-escalation Study	Adults<br>32 enrolled<br>29 dosed<br>26 completed	Up to 4 doses of KRN23 (0.05, 0.1, 0.3 and 0.6 mg/kg) SC every 28 days over a 120-day period.<br>Placebo: (n=1)	- Safety and efficacy of repeat-doses administered SC<br>- PD, PK, and QoL measures	Completed
**KRN23-INT-002**	Phase 1/2 Extension of KRN23-INT-001 Study	Adults<br>23 enrolled<br>23 dosed<br>19 completed	Up to 12 doses of KRN23 (0.05, 0.1, 0.3, 0.6, and 1.0 mg/kg) SC every 28 days over a 120-day period.<br>Placebo: (n=1)	- Safety and efficacy of repeat-doses administered SC<br>- PD, PK, and QoL	Completed
**KRN23-001**	Phase 1, Open-label Single-dose Study	Japanese and Korean Adults<br>18 enrolled<br>18 dosed<br>18 completed	Single-dose KRN23<br>SC: 0.3, 0.6 and 1.0 mg/kg	- Safety and PD<br>- PK	Completed
**UX023-CL203**	Phase 2b Long-term, Open-label Extension of Studies KRN23-INT-001 and KRN23-INT-002	Adults<br>20 enrolled<br>20 dosed	KRN23: 0.3, 0.6, or 1.0 mg/kg SC Q4W for a minimum of 164 weeks and a maximum of 192 weeks.	- Safety<br>- Efficacy<br>- PD, bone health, immunogenicity	Ongoing; enrollment closed

Study Number	Study Design	Number of Subjects	Dosage and Regimen	Key Endpoints	Study Status
**UX023-CL303**	Phase 3, Randomized, Double-Blind, Placebo-Controlled, Fixed Dose Study	Adults<br>(18 – 65 yrs)<br>134 enrolled<br>134 dosed	KRN23: 1.0 mg/kg SC (rounded to the nearest 10 mg; max 90 mg) Q4W for up to 96 weeks (subjects in Japan or Korea) or 149 weeks (subjects in US and EU) or until availability of another mechanism of KRN23<br>Placebo: subjects in the placebo group will receive placebo for 24 weeks, and will then cross over to KRN23 1 mg/kg.	- Efficacy<br>- PD effect<br>- Pain (BPI) and other PROs<br>- Safety	Ongoing; enrollment closed
**UX023-CL304**	Phase 3, Open-label, Fixed Dose Study	Adults<br>(25 – 65 yrs)<br>14 enrolled<br>14 dosed	KRN23 1 mg/kg SC (rounded to the nearest 10 mg; max 90 mg) Q4W for up to 96 weeks (non-US subjects) or 141 weeks (US subjects).	- Bone histomorphometry<br>- PD, PROs<br>- Safety	Ongoing; enrollment closed
**UX023-CL201**	Phase 2, Open-label Repeat-dose, Dose–finding Study	Children<br>(5 – 12 yrs)<br>52 enrolled<br>52 dosed<br>52 completed	KRN23 SC Q2W (0.1 – 2.0 mg/kg) or Q4W (0.2 – 2.0 mg/kg) for a 16-week dose Titration Period and a 48-week Treatment Period, followed by KRN23 SC Q2W for a 96-week Treatment Extension Period. For US subjects, an additional 56-week Treatment Extension Period is included.<br>KRN23 dose is adjusted Q4W during the Titration Period in 0.3 mg/kg increments, and titration may continue during the Treatment Period until the target serum phosphorus range is reached. For subjects receiving KRN23 Q4W, dosing is adjusted at 60% of their Q4W dose during the Treatment Extension period to maintain serum phosphorus in the target range.	- Dose and dose regimen<br>- Safety and PD of repeat-doses<br>- Efficacy<br>- PK and QoL	Completed
**UX023-CL205**	Phase 2, Open-label Study	Children<br>(1 – 4 yrs)<br>13 enrolled<br>13 dosed	KRN23 SC Q2W at a starting dose of 0.8 mg/kg to a maximum of 1.2 mg/kg for 64 weeks, with an additional 96-week Treatment Extension Period.	- Safety and PD<br>- Efficacy<br>- Functional disability and pain<br>- PK	Ongoing; enrollment closed

Study Number	Study Design	Number of Subjects	Dosage and Regimen	Key Endpoints	Study Status
**UX023-CL301**	Phase 3, Open-label, Active-controlled Study	Children (1 to ≤ 12 yrs) 61 enrolled 61 dosed	KRN23 SC Q2W up to 64 weeks. Target dose of 0.8 mg/kg. Dose may be increased to 1.2 mg/kg at any time during the study if serum phosphorus increased by < 0.5 mg/dL from Baseline. The maximum allowable dose is 90 mg.<br>Active control: oral phosphate and active vitamin D individualized for each subject at Investigator's discretion within expert guidelines.<br>For subjects in the US, Canada, EU, and Australia, a 76-week Treatment Extension Period is included, during which subjects in the active-comparator controlled group will crossover to receive KRN23 treatment.	- Efficacy<br>- Safety and PD<br>- Functional disability and pain<br>- PK and QoL	Ongoing; enrollment closed
**KRN23-003**	Phase 3, Open-label Study	Children (1 to ≤ 12 yrs) 15 enrolled 15 dosed	KRN23 SC Q2W up to Week 88 or until commercial product is available, whichever comes first. Target dose of 0.8 mg/kg. Dose may be increased to 1.2 mg/kg at any time during the study if serum phosphorus increased by ≤ 0.5 mg/dL from Baseline.	- Safety<br>- Efficacy<br>- PK<br>- PD	Ongoing; enrollment closed
**KRN23-004**	Phase 3, Open-label, Long-term Extension Study	28 planned<br><br>Adults: 18 enrolled 18 dosed<br><br>Children: 1 enrolled 1 dosed	Adults: KRN23 1 mg/kg SC (max 90 mg) Q4W, until commercial product is available, or the end of September 2020, whichever comes first.<br>Children: Target dose of 0.8 mg/kg (minimum 10 mg) Q2W, until commercial product is available, or the end of September 2020. Dose may be increased to 1.2 mg/kg or 2.0 mg/kg at any time during study if serum phosphorus increased by ≤ 0.5 mg/dL from Baseline.	- Safety<br>- Efficacy<br>- PD effect<br>- PK	Ongoing; enrollment open
**BUR-02**	Phase 3, Open-label, Long-term Extension Study	38 adults planned 24 enrolled 22 dosed	KRN23 1 mg/kg SC (max 90 mg) Q4W for up to 120 weeks.	- Safety<br>- Efficacy<br>- PROs	Ongoing; enrollment open
**TIO and ENS-associated Osteomalacia in Adults**					
**KRN23-002**	Phase 2, Open-label Study	Japanese and Korean Adults 13 enrolled	KRN23 SC Q4W 0.3 – 2.0 mg/kg for up to 140 weeks or until commercial product is available, whichever comes first.	- Efficacy<br>- Safety<br>- PK, PD	Ongoing; enrollment closed

5.4 Summary of Overall Risks and Potential Benefits

The efficacy and safety of KRN23 in XLH has been established in a total of 13 completed and ongoing clinical studies (Section 5.3.3), which include a broad patient population ranging from 1 year of age to adults. In total, more than 200 adults and children with XLH have received KRN23 at various doses (up to 2 mg/kg every 2 weeks) and for durations up to 214 weeks. These studies have demonstrated that KRN23 treatment blocks FGF23 and leads to a sustained increase in serum phosphorus levels and restoration of phosphate homeostasis, thereby improving key manifestations of XLH in children and adults. In children with XLH, KRN23 treatment substantially improved rickets, growth, physical function, and pain. In adults with XLH, KRN23 treatment improved skeletal health, including osteomalacia; improved the healing of fractures and pseudofractures; improved physical function; and reduced subject-reported stiffness and pain.

Across all studies in XLH, KRN23 was well tolerated and demonstrated an acceptable safety profile with no significant safety concerns. Most adverse events were mild to moderate in severity and no KRN23-related deaths have occurred. In the adult placebo-controlled study in XLH, most frequent adverse events related to KRN23 were injection-site reactions in children and restless legs syndrome (RLS) in adults. Injection-site reaction events were mild in severity and readily managed. RLS events were generally mild to moderate in severity and often occurred in the setting of medical or family history of RLS. In adults, serum phosphorus levels above the normal range occurred in < 10% of subjects and were transient, mild, and reversible. No clinically meaningful changes related to potential ectopic mineralization were observed. Although ectopic mineralization is a known risk related to XLH disease, and is exacerbated by the current standard of care consisting of oral phosphate and/or 1,25$(OH)_2$D supplementation, KRN23 does not appear to be associated with progression of cardiac or renal ectopic mineralization beyond the natural course of pre-existing disease.

In conclusion, KRN23 inhibits the effects of FGF23, restoring phosphate, vitamin D, and bone metabolism homeostasis. By targeting FGF23 and correcting hypophosphatemia, it is expected that the underlying osteomalacia will be treated in subjects with TIO and ENS-associated osteomalacia, leading to improved clinical outcomes including a potential reduction in pain, fatigue, and fracture risk, as well as the impact of these factors on the health-related quality of life of adult patients with TIO. To date, KRN23 has a favorable safety profile without evidence of increased ectopic mineralization or other safety concerns associated with excess FGF23. Overall, based on the scientific rationale, unmet medical need, and results from nonclinical studies and clinical studies to date, investigation of KRN23 is warranted in patients with TIO/ENS-associated osteomalacia for whom complete surgical tumor/lesion resection is not a viable treatment option.

6 STUDY OBJECTIVES

The primary objectives of this study are to evaluate the following:

- Effect of KRN23 treatment on increasing serum phosphorus levels in adults with TIO or ENS-associated osteomalacia
- Effect of KRN23 treatment on improvement in TIO/ENS-associated osteomalacia as determined by the following histomorphometric indices:
  - Osteoid thickness (O.Th)
  - Osteoid surface/Bone surface (OS/BS)
  - Osteoid volume/Bone volume (OV/BV)
  - Mineralization lag time (MLt)

Secondary objectives of the study are to evaluate the following:

- The PD profile of KRN23 as assessed by changes from Baseline over time in additional measures of serum phosphorus, serum FGF23, alkaline phosphatase (ALP), and 1,25$(OH)_2$D; TRP and TmP/GFR (the ratio of renal tubular maximum phosphate reabsorption rate to glomerular filtration rate)
- Effects of KRN23 on bone turnover markers, including bone-specific ALP (BALP), carboxy terminal cross-linked telopeptide of type I collagen (CTx), procollagen type 1 N-propeptide (P1NP), and osteocalcin
- Functional outcomes including upper and lower extremity muscle strength, walking and reaching ability, and mobility
- Patient-reported outcomes including self-reported pain, fatigue, and health-related quality of life

The PK objective of the study is to:

- Determine the PK profile of repeat SC injections of KRN23 at Baseline (Weeks 0, 2, and 4) and 6 months (Weeks 20, 22, and 24) in subjects with TIO or ENS-associated osteomalacia

Exploratory objectives of the study are to evaluate the following:

- Changes in additional histomorphometry parameters in trans-iliac crest bone biopsies including both structural and dynamic measures
- Changes in underlying skeletal disease/osteomalacia as assessed by skeletal survey/standard radiographs, dual-energy X-ray absorptiometry (DXA), ^{99m}Tc-labelled bone scan, and high-resolution peripheral quantitative computed tomography (CT) (XtremeCT; where available)

The safety objective of the study is to:

- Assess the safety of KRN23 administration in subjects with TIO or ENS-associated osteomalacia, based on adverse events (AEs), laboratory assessments, cardiac imaging, renal ultrasound, and immunogenic response

7 INVESTIGATIONAL PLAN

7.1 Overall Study Design and Plan

UX023T-CL201 is an open-label, Phase 2 study of KRN23 in adult subjects with TIO or ENS-associated osteomalacia. Approximately 15 subjects with TIO or ENS-associated osteomalacia with inoperable disease (defined as 'the inability to localize the tumor or the impractical nature of surgery due to the extent of disease or the location of the tumor'), will be enrolled. The study will include an initial 48-week Treatment Period, a Treatment Extension Period of up to 252 weeks, and a Safety Follow-up Period of up to 12 weeks from the last dose of study drug to determine the efficacy, safety, PK, and PD of repeat SC injections of KRN23.

Subjects will be brought in for a Screening Visit and asked to provide written informed consent. An extensive review of historical records and images will be conducted to confirm the diagnosis and to determine the extent of the lesion and severity of disease. Subjects will be selected for Screening only if they are deemed to be inoperable for cure. A historical tumor image may be used as the Screening image if it was obtained within 12 months of Screening.

Subjects who are eligible for Screening will discontinue oral phosphate and vitamin D metabolite therapy at least 2 weeks prior to the Screening Visit and may not resume supplementation for the duration of the study. After Screening biochemistry parameters confirm eligibility, subjects will be provided a tetracycline (eg, tetracycline hydrochloride [HCl] and demeclocycline) to take prior to the Baseline Visit to facilitate histomorphometric analysis of the trans-iliac crest bone biopsy (refer to Section 7.5.4.3). Note that the bone biopsy is not required at the Baseline Visit if a previous bone biopsy performed within 12 months of Screening confirmed the diagnosis of osteomalacia, the subjects' clinical manifestations have not changed significantly since the time of the previous biopsy/diagnosis, and the tissue collected at that biopsy is made available for testing for this protocol.

The Baseline Visit will be conducted over approximately 4 days, including overnight stay if deemed necessary by the Investigator. Fasting blood samples and urine collections (of 2- and 24-hour durations), as well as predose Baseline safety and efficacy assessments (*excluding bone biopsy*) will occur on the first 1 to 2 days of the Baseline Visit. On the day following these assessments, a trans-iliac crest bone biopsy will be performed (refer to Section 7.5.4.3). No other assessments are to be performed or any study drug administered on that day. On the day following the biopsy (Day 0, Week 0), subjects will receive their first SC injection(s) of KRN23.

All enrolled subjects will begin treatment with KRN23 every 4 weeks (Q4W) at a starting dose of 0.3 mg/kg (Week 0). Doses of KRN23 will then be titrated in an effort to achieve a target fasting serum phosphorus range of 2.5 to 4.0 mg/dL (details are described in Section 7.4.5). If the subject's peak serum phosphorus level at Week 2 remains below the

target range, the subject's dose may be increased at Week 4 to 0.6 mg/kg, at the discretion of the Investigator. Doses will then continue to be titrated in increments of 0.2 mg/kg at Weeks 8, 12, and 16 in individual subjects to achieve the target peak serum phosphorus range. Doses may be titrated at later visits, at the discretion of the Investigator, if there are concerns about safety or suboptimal efficacy, or if a subject has not yet achieved the target range of serum phosphorus (refer to Section 7.4.5). Doses of KRN23 will be adjusted downward if serum phosphorous levels range from 4.0 to 4.5 mg/dL. Doses of KRN23 will be skipped if serum phosphorous levels exceed 4.5 mg/dL. Doses will be interrupted and may be retitrated if a subject undergoes treatment of the underlying tumor (ie, radiation therapy or excision) at any point during the study (refer to Section 7.4.5).

Subjects will be enrolled consecutively. The incidence, frequency, and severity of AEs and serious AEs (SAEs); AEs leading to discontinuation; deaths; clinically significant laboratory parameters (serum chemistry, hematology, and urinalysis); as well as serum intact parathyroid hormone (iPTH), phosphorus, 1,25(OH)$_2$D, creatinine, calcium:creatinine ratio, and serum and urinary calcium will be reviewed periodically on an ongoing basis coincident with formal data snap shots. If a dose-limiting toxicity (DLT; a Grade ≥ 3 toxicity that is probably or possibly treatment related or a confirmed serum phosphorus level ≥ 6.5 mg/dL) is identified at any point, the study will be paused until all of the efficacy and safety data collected to date are reviewed to evaluate the overall risks/benefits of the study and to make a determination about whether it will continue.

Upon regulatory approval and commercial availability of KRN23 for the treatment of TIO, subjects will have their End of Study (EOS) Visit, and transition to prescription drug, as recommended by their treating physician. Until commercial availability, subjects may continue treatment with KRN23 for up to 252 weeks in the Treatment Extension Period, or until 31 January 2021, whichever is sooner, and then undergo their EOS Visit. Therefore, the duration of the Treatment Extension Period will vary for individual subjects, and will be determined by the time from the start of Week 49 through their EOS Visit. Upon completion of study drug treatment (ie, EOS Visit) or early withdrawal from study (ie, Early Termination [ET] Visit), a Safety Follow-up telephone call (TC) will occur approximately 6 weeks (+ 5 days) after a subject's last dose of study drug to collect information on any ongoing or new AEs, SAEs, and concomitant medications. An additional Safety Follow-up TC will occur approximately 12 weeks (+ 5 days) after the subject's last dose of study drug. If subjects have started receiving KRN23 (burosumab) via an alternative mechanism (eg, commercial treatment) by the time of the 6-week Safety Follow-up TC, the 12-week Safety Follow-up TC may not be necessary. The end of this study is defined as the last day that protocol-specified assessments (including telephone contact) are conducted for the last subject.

Screening, Baseline, and post-treatment PK, PD, efficacy, and safety assessments are shown by study visit in Table 2.1, Table 2.2, Table 2.3, Table 2.4, Table 2.5, and Table 2.6.

7.2 Discussion of Study Design, Including Choice of Control Group

This is a Phase 2 study and is the first study of KRN23 in subjects with TIO or ENS-associated osteomalacia. Therefore, this is an open-label study in which there is no control arm, and all adult subjects will receive investigational product. Subjects will be enrolled sequentially; to minimize any safety risks the first 3 subjects must complete at least the first 2 doses with no safety or tolerability issues before the next 12 subjects may be enrolled.

7.3 Selection of Study Population

This is the first clinical study of KRN23 in subjects with TIO or ENS-associated osteomalacia. This study will be conducted in approximately 15 adult subjects with a clinical diagnosis of TIO or ENS-associated osteomalacia who are likely to benefit from treatment with KRN23 because they are deemed to have inoperable disease (defined by an inability to localize the tumor or the impractical nature of surgery due to the extent of disease or the location of the tumor) or if they have ENS-associated osteomalacia and are not being considered for skin lesion removal treatment.

7.3.1 Inclusion Criteria

Subjects eligible to participate in this study must meet all of the following criteria at Screening:

1. Have a clinical diagnosis of TIO/ENS-associated osteomalacia based on evidence of excessive FGF23 that is not amenable to cure by surgical excision of the offending tumor/lesion (documented by Investigator)
2. Be ≥ 18 years of age
3. Have a fasting serum phosphorus level < 2.5 mg/dL
4. Have serum iFGF23 level ≥ 100 pg/mL by Kainos assay
5. Have a TmP/GFR < 2.5 mg/dL
6. Have an estimated glomerular filtration rate (eGFR) ≥ 60 mL/min (using Cockcroft-Gault formula). Subjects with an eGFR ≥ 30 but < 60 mL/min will be considered eligible so long as in the opinion of the Investigator the decline in renal function is not related to nephrocalcinosis
7. Have a corrected serum calcium level < 10.8 mg/dL
   [Corrected serum calcium = serum calcium in mg/dL + 0.8 × (4 – serum albumin in g/dL)]

8. Females of child-bearing potential must have a negative urine pregnancy test at Screening and Baseline and be willing to have additional pregnancy tests during the study. Females considered not to be of childbearing potential include those who have not experienced menarche, are post-menopausal (defined as having no menses for at least 12 months without an alternative medical cause) or are permanently sterile because of total hysterectomy, bilateral salpingectomy, or bilateral oophorectomy
9. Participants of child-bearing potential or fertile males with partners of child-bearing potential who are sexually active must consent to use a highly effective method of contraception as determined by the site Investigator from the period following the signing of the informed consent through the final Safety Follow-up TC (as defined in Section 7.1)
10. Be willing to provide access to prior medical records to determine eligibility including imaging, biochemical, and diagnostic, medical, and surgical history data
11. Provide written informed consent after the nature of the study has been explained, and prior to any research-related procedures
12. Be willing and able to complete all aspects of the study, adhere to the study visit schedule and comply with the assessments (in the opinion of the Investigator)

7.3.2 Exclusion Criteria

Subjects meeting any of the following criteria must not be enrolled in the study:

1. Have a prior diagnosis of human immunodeficiency virus, hepatitis B, and/or hepatitis C
2. Have a history of recurrent infection, a predisposition to infection, or a known immunodeficiency
3. Are pregnant or breastfeeding at Screening or are planning to become pregnant (self or partner) at any time during the study
4. Have participated in an investigational drug or device trial within 30 days prior to Screening or are currently enrolled in another study of an investigational product or device
5. Have used a therapeutic mAb, including KRN23, within 90 days prior to Screening, or have a history of allergic or anaphylactic reactions to any mAb
6. Have or have a history of any hypersensitivity to KRN23 excipients that, in the judgment of the Investigator, places the subject at increased risk for adverse effects
7. Have used a pharmacologic vitamin D metabolite or its analog (eg, calcitriol, doxercalciferol, and paricalcitol), phosphate, or aluminum hydroxide antacids (eg, Maalox® and Mylanta®) within 2 weeks prior to Screening or during the study
8. Have used medication to suppress parathyroid hormone (PTH) (eg, Sensipar, cinacalcet, calcimimetics) within 2 months prior to Screening

9. Have a history of malignancy within 5 years of study entry with the exception of PMT-MCT (Phosphaturic mesenchymal tumors of the mixed connective tissue type) tumors or non-melanoma skin cancers such as basal cell skin cancer
10. Have donated blood or blood products within 60 days prior to Screening
11. Have a history of allergic reaction to or have shown adverse reactions to a tetracycline (eg, tetracycline HCl and demeclocycline), benzodiazepines, fentanyl, or lidocaine
12. Have any condition, which in the opinion of the Investigator and Sponsor, could present a concern for either subject safety or difficulty with data interpretation

7.3.3 Removal of Subjects from Therapy or Assessment

In accordance with the Declaration of Helsinki, subjects have the right to withdraw from the study at any time for any reason. The Investigator may withdraw a subject at any time at their discretion. Ultragenyx must be notified of all subject withdrawals as soon as possible. Ultragenyx also reserves the right to discontinue the study at any time for either clinical or administrative reasons and to discontinue participation of an individual subject or Investigator because of poor enrollment or noncompliance, as applicable.

Subjects may be removed from the study for the following reasons:

- Occurrence of an unacceptable AE
- Any clinical deterioration or worsening of serum biochemistries
- An illness that, in the judgment of the Investigator or Ultragenyx, might place the subject at risk or invalidate the study
- At the request of the subject, Investigator, or Ultragenyx, for administrative or other reasons
- Protocol deviation or noncompliance
- Pregnancy in subject

In addition, a subject may be removed if a previously unidentified causal tumor is identified and determined to be amenable to complete resection, consistent with the inclusion criterion requiring a tumor that is not amenable to cure by surgical excision.

If the reason for removal of a subject from the study is an AE, the AE and any related test or procedure results will be recorded in the source documents and transcribed onto the Case Report Form (CRF). AEs should be followed until the abnormality stabilizes, resolves, or until a decision is made that it is not likely to resolve. If AEs do not return to baseline within 30 days after the last dose given, their etiology should be identified and Ultragenyx should be notified. All unscheduled tests must be reported to Ultragenyx immediately.

If a subject discontinues from the study prematurely, every reasonable effort should be made to perform the ET Visit procedures within 4 weeks of discontinuation (Table 2.6).

Subjects who withdraw or are removed from the study after receiving study drug may be replaced on a case-by-case basis, at the discretion of Ultragenyx and the Principal Investigator.

7.3.3.1 Stopping Rules

Individual subjects who experience a Grade 3 or higher SAE that is unexpected and possibly, probably, or definitely related to KRN23 (Section 8.5.3) will be assessed by the Investigator and reviewed with the Medical Monitor to determine if any actions are needed. In these cases, a full clinical investigation will be performed to make a decision regarding what actions to take, including whether to recommend stopping the study drug and/or discontinuing the subject from the study and informing the Institutional Review Board/Ethics Committee (IRB). Subjects who experience unacceptable toxicity as determined by the full clinical investigation will be withdrawn from study treatment.

Individual subjects will be monitored for ectopic mineralization by renal ultrasound and echocardiogram (ECHO). If new or clinically significant worsening in mineralization is considered clinically meaningful by the Investigator and/or Sponsor and related to study drug, the subject will be discontinued from the study.

In addition, while the study is open for enrollment, after the first 3 subjects have completed the Week 8 assessments, those 3 subjects will continue on the study and the safety data collected to date will be reviewed by the Sponsor to determine whether to continue adding subjects to the study. If a dose limiting toxicity is identified, the study will be paused while all of the efficacy and safety data collected to date are reviewed to evaluate the overall risk-benefit of the study and make a determination about whether it will continue.

7.4 Treatments

7.4.1 Treatments Administered

This is an open-label study of the investigational product KRN23. All subjects will receive KRN23 treatment for up to 300 weeks, or until 31 January 2021, whichever is sooner, after which subjects will have their EOS Visit. Upon regulatory approval and commercial availability of KRN23 for the treatment of TIO, subjects will have their EOS Visit, and transition to prescription drug, as recommended by their treating physician. Subjects will undergo an ET Visit if 1 of the following occurs: the subject withdraws consent and discontinues from the study, the subject is discontinued from the study at the discretion of the Investigator or Ultragenyx, or the study is terminated. KRN23 will be administered Q4W. All enrolled subjects will begin treatment with KRN23 at a starting dose of 0.3 mg/kg (Week 0). Doses of KRN23 will then be titrated Q4W in an effort to achieve a target fasting serum phosphorus range of 2.5 to 4.0 mg/dL (as described in Section 7.4.5). All serum phosphorus assessments will be taken with subjects in the fasting state.

If the subject's peak serum phosphorus level at Week 2 remains below the target range, the subject's dose may be increased at Week 4 to 0.6 mg/kg, at the discretion of the Investigator.

Doses will then continue to be titrated in increments of 0.2 mg/kg at Weeks 8, 12, and 16 in individual subjects to achieve the target peak serum phosphorus range. Doses may be titrated at later visits, at the discretion of the Investigator, if there are concerns about safety or suboptimal efficacy or if a subject has not yet achieved the target range of serum phosphorus (refer to Section 7.4.5).

Doses of KRN23 will be adjusted downward if serum phosphorous levels range from 4.0 to 4.5 mg/dL. Doses of KRN23 will be skipped if serum phosphorous levels exceed 4.5 mg/dL. Doses will be interrupted and may be retitrated if a subject undergoes treatment of the underlying tumor (ie, radiation therapy or excision) at any point during the study (refer to Section 7.4.5). Following a downward dose adjustment, the Investigator together with the Medical Monitor will determine how and when to dose titrate up.

The maximum dose allowed in this protocol is 2.0 mg/kg every 2 weeks (Q2W) (refer to Section 7.4.5). If needed, the final dose adjustment increment may be less than the specified increment to reach the 2.0 mg/kg dose.

7.4.2 Identity of Investigational Product(s)

7.4.2.1 Investigational Test Product

KRN23 is supplied as a sterile, clear, colorless, and preservative-free solution in single-use 5-mL vials containing 1 mL of KRN23 (30 mg) at a concentration of 30 mg/mL. KRN23 should be securely stored at 2 to 8°C and protected from light. It should not be frozen. KRN23 will be administered SC without dilution. Subjects will receive study drug via SC injection to the abdomen, upper arm, or thigh; the injection site will be rotated with each injection, including to a different quadrant of the abdomen. No more than 1.5 mL may be administered in a single injection. If a subject needs more than 1.5 mL per administration, multiple injections must be administered and a different site must be used for each injection.

7.4.2.2 Placebo or Active Comparator

This is an open-label study in which all subjects will receive investigational product.
No placebo or reference therapy will be administered.

7.4.3 Method of Assigning Subjects to Treatment Groups

All subjects will receive the same investigational product, KRN23, and will follow the same dose regimen. Therefore, there is no randomization in this study.

7.4.4 Selection of Doses in the Study

In the first-in-human, single-dose, dose-escalation study of KRN23 (KRN-US-02), IV and SC doses were evaluated in subjects with XLH. SC doses ranged from 0.1 to 1.0 mg/kg, and meaningful increases in serum phosphorus, TmP/GFR, and serum 1,25(OH)$_2$D levels were observed beginning at the 0.3 mg/kg dose level.

In a Phase 1/2 multiple-dose, dose-escalation study (KRN23-INT-001), KRN23 was well tolerated following SC administration of 4 intra-subject escalating doses (0.05 mg/kg → 0.1 mg/kg → 0.3 mg/kg →0.6 mg/kg) administered Q4W in subjects with XLH. The proportion of KRN23-treated subjects with serum phosphorus levels in the target range (> 2.5 to ≤ 4.0 mg/dL) increased with KRN23 dose level but did not exceed the upper limit of normal (4.5 mg/dL) in any subject at any time point. A direct PK-PD relationship between serum KRN23 concentrations and serum phosphorus levels was noted in the study.

In an associated extension study (KRN-INT-002), doses were individually selected for each subject (up to 1.0 mg/kg every 4 weeks) based on serum phosphorus response (target range > 2.5 to ≤ 4.0 mg/dL) and safety considerations. Doses were relatively stable in the extension study and were generally either 0.6 or 1.0 mg/kg. Doses up to 1.0 mg/kg administered every 4 weeks were well tolerated over a period of 48 weeks.

In the ongoing pediatric study in patients with XLH, KRN23 doses up to 2.0 mg/kg administered Q2W or Q4W are permitted.

Based on these initial results in subjects with XLH, a starting dose of 0.3 mg/kg, with additional KRN23 injections Q4W, was selected for this study. Although the maximum dose administered to date in adults with XLH is 1.0 mg/kg Q4W, the maximum dose in this study is 2.0 mg/kg Q2W. This dose was selected because it is anticipated that doses > 1.0 mg/kg may be needed to bring serum phosphorus levels up to the lower end of the normal range in subjects with TIO. Compared to subjects with XLH, FGF23 levels are much higher in subjects with TIO and ENS-associated osteomalacia and the associated hypophosphatemia is more severe. It is important to note that dose escalation will be limited based on individual subject serum phosphorus response (see Section 7.4.5), and no off-target effects have been observed to date in nonclinical or clinical studies. Specific safety monitoring procedures, based on the target effects of KRN23, have been selected to mitigate safety risk to subjects (see Section 7.5.6).

7.4.5 Selection and Timing of Dose for Each Subject

KRN23 will initially be administered Q4W. All subjects will receive a KRN23 starting dose of 0.3 mg/kg (at Week 0). Doses of KRN23 will then be titrated in an effort to achieve a target fasting peak serum phosphorus range of 2.5 to 4.0 mg/dL based on peak serum phosphorus samples obtained 2 weeks after drug administration (described in Table 7.4.5.1). If the subject’s peak serum phosphorus level at Week 2 remains below the target range, the subject’s dose may be increased at Week 4 to 0.6 mg/kg, at the discretion of the Investigator. Doses will then continue to be titrated in increments of 0.2 mg/kg at Weeks 8, 12, and 16 in individual subjects to achieve the target peak serum phosphorus range.

Table 7.4.5.1: KRN23 Initial Dose Titration Scheme

Study Week	Peak Serum Phosphorus Level[1]	Dose Adjustment
Week 2	< 2.5 mg/dL	Increase the dose by 0.3 mg/kg to 0.6 mg/kg at Week 4
Weeks 8, 12, and 16	< 2.5 mg/dL	Increase the dose by 0.2 mg/kg[2]
	2.5 – 4.0 mg/dL	Repeat the current dose
	> 4.0 – 4.5 mg/dL	Reduce the dose by 0.2 mg/kg
	> 4.5 mg/dL	Skip dose

1 Peak fasting serum phosphorus level is the phosphorus level at 2 weeks post dose. Peak fasting serum phosphorus levels will be measured through Week 16 and at Week 22 or at unscheduled visits if needed. Thereafter, dosing decisions will be made based on trough serum phosphorus levels (4 weeks after dose and prior to the subsequent dose) obtained at the previous study visit as indicated in Table 7.4.5.2. Local labs may be obtained on the day of dosing (prior to the injection) if confirmation of serum phosphorus level is needed to determine whether to maintain the current dose level or to titrate the dose.

2 If a subject's fasting peak serum phosphorus level has not increased (defined as a change ≤ 0.2 mg/dL) after 2 consecutive dose escalations, even if the target peak serum phosphorus range has not been achieved, then the current dose will be considered that subject's maintenance dose and will not be escalated further.

Doses may be titrated at later visits, following the initial dose titration phase, at the discretion of the Investigator, if there are concerns about safety, suboptimal efficacy, or if a subject has not yet achieved the target range of serum phosphorus. To optimize serum phosphorus levels throughout the dose cycle, Investigators may increase the dose of KRN23 in 0.5 mg/kg increments in those subjects whose trough serum phosphorus level (4 weeks postdose, just prior to the next dose) is below the lower limit of normal (< 2.5 mg/dL) on 2 consecutive measurements performed at 4-week intervals (Table 7.4.5.2). Trough levels of serum phosphorus may be evaluated through unscheduled assessments, if necessary, until a new maintenance dose is established (defined as a dose at which 2 consecutive trough serum phosphorous levels are within the normal range [2.5 – 4.0 mg/dL]). Dose titration may continue up to a dose of 2.0 mg/kg Q4W, until fasting trough serum phosphorus is ≥ 2.5 mg/dL (Table 7.4.5.2).

If, after a subject reaches a dose of 2.0 mg/kg Q4W, the trough serum phosphorus level remains < 2.5 mg/dL on 2 consecutive trough assessments performed at 4-week intervals beginning after the second administration of the 2 mg/kg dose, the dosing frequency may be increased to Q2W (Table 7.4.5.2). Subjects transitioning to Q2W dosing should initially receive 60% of their established Q4W total dose level (equivalent to 1.2 mg/kg). Predose serum phosphorus levels will then be followed through assessments every 4 weeks (prior to subsequent doses) for 12 weeks. If, after 12 weeks from the first Q2W dose, predose serum phosphorus remains < 2.5 mg/dL, the dose may be increased to 2.0 mg/kg Q2W. Predose serum phosphorus levels will then be followed through assessments every 4 weeks (prior to subsequent doses) for 12 weeks to ensure that subjects do not experience DLT (Section 7.5.6.13).

Following a downward dose adjustment, the Investigator together with the Medical Monitor will determine how and when to dose titrate up.

The maximum dose allowed in this study is 2.0 mg/kg Q2W.

KRN23 will continue to be dosed Q4W or Q2W throughout the study, either at home using home health services or at the study site.

Table 7.4.5.2: KRN23 Dose Titration Scheme for Dose Adjustments and Increases in Dose Frequency from Q4W to Q2W At or After Week 96

Criteria for Dose Adjustments Based on Trough Serum Phosphorous Levels		
**Current Dose and Frequency**	**Trough Serum Phosphorus Level[1]**	**Dose/Frequency Adjustment**
< 2.0 mg/kg, Q4W	2 consecutive levels < 2.5 mg/dL at current dose	Increase the dose by 0.5 mg/kg, up to 2.0 mg/kg Q4W
	2.5 – 4.0 mg/dL	Repeat the current dose
	> 4.0 – 4.5 mg/dL	Reduce the dose by 0.5 mg/kg[2]
	> 4.5 mg/dL	Skip dose
2.0 mg/kg, Q4W	2 consecutive levels < 2.5 mg/dL, beginning after the second administration of the 2 mg/kg dose	Initiate Q2W dosing at 60% of the Q4W total dose (equivalent to 1.2 mg/kg)
1.2 mg/kg, Q2W	Levels remain < 2.5 mg/dL after 12 weeks on initial Q2W dose[3]	Increase dose to 2.0 mg/kg Q2W[4]

1 Trough serum phosphorous levels will be measured every 4 weeks, just prior to the next dose. Serum phosphorus levels may be measured at unscheduled visits if necessary.

2 Following a downward dose adjustment, the Investigator together with the Medical Monitor will determine how and when to dose titrate up.

3 Dosing decisions will be based on fasting trough serum phosphorus levels obtained every 4 weeks (prior to dosing) for 12 weeks. Local labs may be obtained on the day of dosing (prior to the injection) if confirmation of serum phosphorus level is needed to determine whether to maintain the current dose level or to titrate the dose.

4 Predose serum phosphorus levels will be followed through assessments every 4 weeks (prior to subsequent doses) for 12 weeks after initiation of 2.0 mg/kg Q2W dosing to ensure that subjects do not experience DLT (Section 7.5.6.13).

7.4.5.1 Dosing in Subjects Undergoing Treatment of Underlying Tumor

If a subject undergoes treatment of the underlying tumor (ie, radiation therapy or excision) at any point during the study, KRN23 treatment should be interrupted. If the Investigator with the study Medical Monitor determines, based on serum phosphorus levels below the lower limit of normal, that the subject should resume KRN23 treatment, the dose should be restarted at 0.3 mg/kg Q4W. Serum phosphorus levels should be subsequently monitored every 4 weeks for 24 weeks following the resumption of KRN23 treatment through unscheduled blood draws at peak (2 weeks postdose ± 3 days) time points. The KRN23 dose will be titrated based on peak serum phosphorous levels as indicated in Table 7.4.5.3. Thereafter, dosing decisions will be made based on trough serum phosphorus levels (4 weeks ± 3 days postdose and prior to the subsequent dose) obtained at the previous study visit as indicated in Table 7.4.5.2, and serum phosphorus measurements will proceed according to the Schedule of Events (Table 2.1, Table 2.2, Table 2.3, Table 2.4, Table 2.5, and Table 2.6).

Table 7.4.5.1.1: KRN23 Dose Titration Scheme Following Treatment of an Underlying Tumor

<table>
<tr><th colspan="2">Criteria for Resuming KRN23 Treatment</th><th>Initial Dose</th></tr>
<tr><td colspan="2">If the Investigator with the study Medical Monitor determines, based on serum phosphorous below the lower limit of normal (< 2.5 mg/dL) that the subject should resume KRN23 treatment.</td><td>The dose should be initiated at 0.3 mg/kg Q4W.</td></tr>
<tr><th colspan="3">Subsequent Criteria for Dose Adjustments Based on Peak Serum Phosphorous Levels[1]</th></tr>
<tr><th>Visit</th><th>Peak Serum Phosphorous Level</th><th>Dose Adjustment</th></tr>
<tr><td>After initial dose</td><td>If below target range of 2.5 – 3.5 mg/dL</td><td>Increase the dose by 0.3 mg/kg to 0.6 mg/kg at 4 weeks post resuming KRN23</td></tr>
<tr><td rowspan="4">After subsequent doses through 24 weeks post resuming KRN23</td><td>< 2.5 mg/dL</td><td>Increase the dose by 0.2 mg/kg</td></tr>
<tr><td>2.5 – 4.0 mg/dL</td><td>Repeat the current dose</td></tr>
<tr><td>> 4.0 – 4.5 mg/dL</td><td>Reduce the dose by 0.2 mg/kg</td></tr>
<tr><td>> 4.5 mg/dL</td><td>Skip dose</td></tr>
</table>

[1] Peak fasting serum phosphorus level is the phosphorus level 2 weeks after a KRN23 dose. Peak fasting serum phosphorus levels will be measured through 24 weeks post resuming KRN23, with dosing decisions made based on peak serum phosphorous levels as indicated. Unscheduled visits can be utilized for assessments as needed. Thereafter (> 24 weeks after resuming KRN23), dosing decisions will be made based on trough serum phosphorus levels (4 weeks ± 3 days postdose and prior to the subsequent dose) obtained at the previous study visit as indicated in Table 7.4.5.2. Local labs may be obtained on the day of dosing (prior to the injection) if confirmation of serum phosphorus level is needed to determine whether to maintain the current dose level or to titrate the dose.

7.4.6 Blinding

This is an open-label study in which all subjects will receive investigational product, and doses will be individually titrated. Therefore, no blinding or masking procedures of the investigational product are necessary.

7.4.7 Prior and Concomitant Therapy

Throughout the study, there should be no significant changes to a subject's diet or medication schedule unless medically indicated. Investigators may prescribe any concomitant medications or treatments deemed necessary to provide adequate supportive care, except those listed in Section 7.4.7.1. All concomitant medications taken during the study will be recorded in the CRF with indication, dose information, and dates of administration.
Any changes to concomitant medication will also be documented. Any intervention to treat the underlying tumor (ie, radiation therapy or resection) should also be recorded. The Investigator should notify the Medical Monitor before initiating treatment of the underlying tumor.

7.4.7.1 Prohibited Medications

To be eligible for the study, subjects must agree to discontinue use of certain medications for the indicated timeframe prior to dosing with KRN23. These medications will remain prohibited throughout the conduct of the study. Any subject who resumes or requires the use of any of these medications during the Treatment Period or Treatment Extension Period will be discontinued from the study.

One exception is in the case of a subject who needs to discontinue treatment with KRN23 for more than 4 weeks to undergo treatment of the underlying tumor. In this case, treatment with oral phosphate and/or pharmacologic vitamin D metabolites may be necessary. Initiation of oral phosphate/vitamin D should not begin before 4 weeks after the last dose of KRN23. Before resuming KRN23 treatment, 14-day washout of oral phosphate/vitamin D should occur, and serum phosphorus below the lower limit of normal should be confirmed (refer to Section 7.4.5).

- Pharmacologic vitamin D metabolites or analogs (eg, calcitriol, doxercalciferol, and paricalcitol): 14-day washout required prior to Screening and during study
- Oral phosphate: 14-day washout required prior to Screening and during study
- Aluminum hydroxide antacids (eg, Maalox® and Mylanta®): 14-day washout required prior to Screening and during study
- Chronic use of systemic corticosteroids (short courses acceptable if indicated)
- PTH suppressors (eg, Sensipar®, cinacalcet)
- Chemotherapeutic agents

- Any mAb therapy (other than study drug)

NOTE: Oral phosphate treatment must be down-titrated slowly to avoid hypercalciuria. Vitamin D metabolites or analogs may be discontinued without titration.

7.4.7.2 Permitted Medications

Other than the medications specifically prohibited in this protocol, subjects may receive concomitant medications as required. If serum 25-hydroxy vitamin D (25[OH]D) levels fall below 20 ng/mL, oral supplementation with vitamin D, eg, multivitamins (non-active vitamin D metabolites) may be provided. Medications (investigational, prescription, over-the-counter, and herbal) and nutritional supplements taken during the 30 days prior to Screening Visit will be reviewed and recorded.

7.4.8 Treatment Compliance

Trained personnel will administer study drug by SC injection at the investigational site or during home health visits as indicated in the Schedule of Events (Table 2.1, Table 2.2, Table 2.3, Table 2.4, Table 2.5, and Table 2.6). Each administration of study drug will be recorded on the CRF. If a subject does not receive a dose within 21 days of a scheduled dose, that dose should be skipped and the next dose will be administered at the next scheduled dosing visit.

7.5 Study Procedures and Assessments

Subjects will be brought in for a Screening Visit and asked to provide written informed consent. Prior to the visit, extensive review of historical records and images will be conducted to confirm the diagnosis, and to determine the extent of the lesion and severity of disease. Subjects will be selected for Screening only if they are deemed to be inoperable for cure.

Subjects who are eligible for Screening will discontinue oral phosphate and vitamin D metabolite therapy at least 2 weeks prior to the Screening Visit and may not resume supplementation for the duration of the study. After Screening biochemistry parameters to confirm eligibility, subjects will be provided a tetracycline (eg, tetracycline HCl and demeclocycline) to take prior to the Baseline Visit to facilitate histomorphometric analysis of the trans-iliac crest bone biopsy. Subjects will be contacted by telephone within 1 week of the Screening Visit and directed to take a tetracycline on Days −20, −19, and −18 prior to the Baseline Visit. A second telephone call to the subject will occur at least 7 days prior to the Baseline Visit and will direct the subject to begin taking a tetracycline on Days −8, −7, and −6 prior to the Baseline Visit. XtremeCT of the radius and tibia will be performed between the Screening and Baseline Visit (when available).

All enrolled subjects will begin treatment with KRN23 Q4W at a starting dose of 0.3 mg/kg (Week 0). Doses of KRN23 will then be titrated in an effort to achieve a target fasting serum phosphorus range of 2.5 to 4.0 mg/dL (details are described in Section 7.4.5).

If the subject's peak serum phosphorus level at Week 2 remains below the target range, the subject's dose may be increased at Week 4 to 0.6 mg/kg, at the discretion of the Investigator. Doses will then continue to be titrated in increments of 0.2 mg/kg at Weeks 8, 12, and 16 in individual subjects to achieve the target peak serum phosphorus range. Doses may be titrated at later visits, at the discretion of the Investigator, if there are concerns about safety or suboptimal efficacy (refer to Section 7.4.5). Doses will also be titrated if a subject undergoes treatment of the underlying tumor (ie, radiation therapy or excision) at any point during the study (refer to Section 7.4.5).

The Baseline Visit will be conducted over approximately 4 days, including overnight stay if deemed necessary by the Investigator. Fasting blood samples, urine collections (of 2- and 24-hour durations), imaging, and assessments of upper and lower extremity strength and function, walking and reaching ability, mobility and self-reported pain, disability and quality of life will occur on the first 1-2 days of the Baseline Visit. On the day following these assessments, a trans-iliac crest bone biopsy will be performed; the bone biopsy is not required at the Baseline Visit if a previous bone biopsy confirmed the diagnosis of osteomalacia, the subjects' clinical manifestations have not changed significantly since the time of the previous biopsy/diagnosis, and the tissue collected at that biopsy is made available for testing for this protocol. No other assessments are to be performed and no study drug is to be administered on that day. The first dose of study drug will be administered the day after the biopsy is performed. Subcutaneous injections will be given in the Clinical Research Unit with the volume to be determined based on the weight measurement obtained on Day −2 (the day prior to dosing) or Day 0 (the day of dosing). Blood samples for PK and PD analyses will be obtained on Day 1 (24 hours following the initial dose). Subjects will be released from the Clinical Research Unit on Day 1 following the completion of all Day 1 assessments. Release may be delayed at the discretion of the Investigator if prolonged recovery from the biopsy procedure is needed.

Subjects will receive KRN23 treatment for up to 300 weeks, or up to 31 January 2021, whichever is sooner, after which subjects will have their EOS Visit. Upon regulatory approval and commercial availability of KRN23 for the treatment of TIO, subjects will have their EOS Visit, and transition to prescription drug, as recommended by their treating physician (Table 2.6).

7.5.1 Schedule of Events

Parameters to be assessed, along with timing of assessments, are provided in Table 2.1, Table 2.2, Table 2.3, Table 2.4, Table 2.5, and Table 2.6.

7.5.2 Primary Efficacy Assessments

First line treatment for TIO is complete tumor resection. However, when either the tumor cannot be localized or cannot be completely resected, the current standard of care is treatment with phosphorus supplementation, usually along with active vitamin D. The primary goals of this treatment are to:

- Maintain serum phosphorus levels at the lower end of the age-appropriate normal range
- Improve or heal osteomalacia, thereby improving its associated symptoms, including fractures, bone and muscle pain, and muscle weakness and loss of function.

Thus, the primary efficacy assessments in the study are as follows:

- *Serum phosphorus:* KRN23 binds to and inhibits FGF23. FGF23 plays an important role as a specific regulator of serum phosphorus levels. Increasing serum phosphorus directly bears on the hypophosphatemia and consequent osteomalacia that characterize TIO and ENS-associated osteomalacia. Serum phosphorus levels will be measured pre- and post-treatment as indicated in the Schedule of Events (Table 2.1, Table 2.2, Table 2.3, Table 2.4, Table 2.5, and Table 2.6).
- *Trans-iliac crest bone biopsy:* To assess O.Th, OS/BS, OV/BV, MLt as well as other parameters, a bone biopsy will be performed pre- and post-treatment as indicated in the Schedule of Events (Table 2.1 and Table 2.2). Note that the bone biopsy is not required at the Baseline Visit if a previous bone biopsy performed within 12 months of Screening confirmed the diagnosis of osteomalacia, the subjects' clinical manifestations have not changed significantly since the time of the previous biopsy/diagnosis, and the tissue collected at that biopsy is made available for testing for this protocol. Bone biopsy histomorphometry will be interpreted centrally by trained personnel who are blinded to other subject data.

7.5.3 Additional Pharmacodynamic Measurements

In addition to the effects on serum phosphorus, the PD effect of KRN23 will be assessed in adults with TIO or ENS-associated osteomalacia, by measuring the following:

- Urinary phosphorus
- Serum FGF23
- Serum ALP
- Serum 1,25$(OH)_2$D
- TmP/GFR and TRP
- Biomarkers of bone remodeling (from blood), including BALP, CTx, P1NP, and osteocalcin

7.5.4 Clinical Assessments

Clinical efficacy measures will evaluate the effect of KRN23 on bone health and functional outcomes in adults with TIO or ENS-associated osteomalacia. Efficacy measures will assess bone health (Section 7.5.4.3), clinical outcomes (Section 7.5.4.1), and subject reported

outcomes (Section 7.5.4.2). Refer to the Study Reference Manual for additional details on clinical efficacy measures.

7.5.4.1 Clinical Outcomes Measurements

Gross motor impairment, including diminished walking ability, pain, and muscle weakness are potential complications associated with TIO-related skeletal deformities. Endpoints to establish clinical outcomes associated with KRN23 treatment include upper and lower extremity strength and function, walking, and reaching ability and mobility.

Hand-Held Dynamometry (HHD): To assess muscle strength, HHD will be administered pre- and post-treatment as indicated in the Schedule of Events (Table 2.1 and Table 2.2). Formal training will be conducted with the trained clinicians administering the HHD testing to standardize technique and minimize variability. The maximum voluntary isometric contraction against a dynamometer will be used to measure bilateral strength in the following muscle groups: elbow flexors, elbow extensors, knee flexors and knee extensors. Absolute values will be recorded, and the percent of predicted values will be calculated using published normative data based on age and gender (Bohannon 1997; Peters et al. 2011).

Sit-to-stand (STS): To assess lower extremity strength and mobility, the STS test will be administered pre- and post-treatment as indicated in the Schedule of Events (Table 2.1, Table 2.2). Formal training will be conducted with the trained clinicians administering the STS test to standardize technique and minimize variability. Absolute values will be recorded.

Weighted Arm Lift (WAL): To assess upper extremity strength, mobility and reaching ability, the WAL test will be administered pre- and post-treatment as indicated in the Schedule of Events (Table 2.1 and Table 2.2). Formal training will be conducted with the trained clinicians administering the WAL test to standardize technique and minimize variability. Absolute values will be recorded.

6-Minute Walk Test (6MWT): The 6MWT will be administered by a trained clinician (preferably a licensed physical therapist) in accordance with general principles set forth in the American Thoracic Society guidelines (ATS 2002). Subjects will be instructed to walk the length of a premeasured course for 6 consecutive minutes. The total distance walked at the end of 6 minutes will be recorded in meters. The percent predicted values for the 6MWT will be calculated using published normative data based on age, gender, and height (Gibbons et al. 2001).

The 6MWT will be administered at Screening for practice purposes to minimize training effects. The 6MWT will also be administered at Baseline (Day –2), and Weeks 12, 24, and 48. Assistive devices may be used; any use will be noted on the CRF.

7.5.4.2 Patient Reported Outcomes Measurements

Pain, fatigue, and physical and mental health and quality of life will be assessed using validated self-report measures, including the brief pain inventory (BPI), the brief fatigue inventory (BFI) and the 36-item short form health survey (SF-36).

BPI: The BPI will be administered at pre- and post-treatment time points as indicated in the Schedule of Events (Table 2.1, Table 2.2, Table 2.3, Table 2.4, Table 2.5, and Table 2.6) to assess pain severity and the impact of pain on daily functioning as measured by subject self-report.

BFI: The BFI will be administered at pre- and post-treatment time points as indicated in the Schedule of Events (Table 2.1, Table 2.2, Table 2.3, Table 2.4, Table 2.5, and Table 2.6) to assess fatigue severity and the impact of fatigue on daily functioning as measured by subject self-report.

SF-36: The SF-36 will be administered at pre- and post-treatment time points as indicated in the Schedule of Events (Table 2.1, Table 2.2, Table 2.3, Table 2.4, Table 2.5, and Table 2.6) to assess physical and mental health based on summary and subscale scores in: vitality, physical functioning, bodily pain, general health perceptions, physical role functioning, emotional role functioning, social role functioning, and mental health.

7.5.4.3 Bone Health Measurements

^{99m}Tc-labelled Bone Scans: Whole body bone scans will be performed to evaluate bone condition and areas of bone damage, including current or prior long bone fractures and the presence of pseudo-fractures. Scans will be performed pre- and post-treatment as indicated in the Schedule of Events (Table 2.1, Table 2.2, Table 2.3 and Table 2.6), and pre- and post-treatment scans will be compared by a central reader who is blinded to time point and subject data.

Dual-energy X-ray absorptiometry: Bone mineral density and content of the lumbar spine and hip will be assessed by DXA at pre- and post-treatment time points as indicated in the Schedule of Events (Table 2.1, Table 2.2, and Table 2.3).

Radiographic Assessments: At Screening, skeletal survey (ie, standard radiographs) will be obtained of the lateral spine, AP chest, right and left hand/wrist, right and left humerus, right and left radius/ulna, right and left femur/pelvis, right and left tibia/fibula, and right and left foot as well as any location(s) where the subject is currently experiencing tenderness or pain that may reflect underlying pathology, or where the subject has a history of recent (< 3 months) fracture(s), or in bones where the bone scan suggests the presence of pseudo-fracture, non-vertebral fracture, vertebral fracture or other finding likely deemed related to osteomalacia. During follow-up, if a subject develops new symptoms of hip pain, rib pain or leg/femur pain, targeted radiographs will be ordered to assess for the presence of new fractures or pseudo-fractures. Follow-up targeted radiographs will be completed in the anatomical location where a fracture or pseudo-fracture was identified every 12 weeks during

the first 48 weeks (at Weeks 24, 36, and 48) and every 24 weeks starting at Week 72, until resolution or up to Week 240, as indicated in the Schedule of Events (Table 2.1, Table 2.2, Table 2.3, Table 2.4, Table 2.5, and Table 2.6). In addition, at Week 144 (Table 2.4) a skeletal survey will be performed. The skeletal survey includes standard radiographs of the chest, lateral spine, right and left hand/wrist, right and left humerus, right and left radius/ulna, right and left femur/pelvis, right and left tibia/fibula, and right and left foot. The skeletal survey at Week 144 will be used to capture the 24-week follow-up targeted radiographs for identified fractures or pseudo-fractures.

Radiographs will be interpreted locally for the identification of new abnormalities, and assessed centrally for changes over time. Central readers will be blinded to other subject data.

XtremeCT: XtremeCT scan of the radius and tibia will be performed (when available) pre- and post-treatment as indicated in the Schedule of Events (Table 2.1, Table 2.2, Table 2.3 and Table 2.4). As an exploratory efficacy measure, XtremeCT will be used to assess bone mineral density and content at the cortical and trabecular compartment. XtremeCT administration procedures will be standardized and results will be read locally by trained site personnel who are blinded to other subject data.

7.5.4.4 Ambulatory Assessment

To assess ambulatory status, subjects will be asked whether they typically walk within the community unassisted, need to use an assistive device (eg, cane, crutches, walker), or cannot walk. Ambulatory status at Baseline will be recorded retrospectively. Prospective assessment of ambulatory status will occur at all site visits beginning from the first site visit after appropriate approvals and consents are obtained and then as indicated on the Schedule of Events (Table 2.3, Table 2.4, Table 2.5, and Table 2.6).

7.5.5 Pharmacokinetic Measurements

To assess KRN23 concentration and possible accumulation, serum levels of KRN23 will be evaluated as a PK parameter in this study. Predose and postdose blood samples will be obtained as indicated in the Schedule of Events (Table 2.1, Table 2.2, Table 2.3, Table 2.4, Table 2.5, and Table 2.6). Stored stable samples may be retrospectively evaluated once proper approvals and consents are obtained.

7.5.6 Safety Measurements

7.5.6.1 Medical History

General medical information includes subject demographics (date of birth, ethnicity, and sex) and a history of major medical illnesses, diagnoses, and surgeries. The review will also include an assessment of symptoms and conditions associated with TIO/ENS-associated osteomalacia and standard of care treatment.

Subjects must be willing to provide access to prior medical records for the collection of historical biochemical and radiographic data, as well as disease history. The specific diagnosis of TIO/ENS-associated osteomalacia will be recorded, along with date of onset, clinical presentation, and date and method of diagnosis.

TIO/ENS-associated osteomalacia treatment history (including any prior attempts of surgical removal) and relevant concomitant medications will be recorded (start date, stop date, dose, dose regimen). Treatments may include calcitriol and oral phosphate. Medications include investigational, prescription, over-the-counter, herbal and nutritional supplements.
Any relevant concomitant therapy, including physical/occupational therapy will be recorded.

PHEX mutation analysis will be performed at Week 96 or at the next visit after appropriate approvals and consents are obtained for any subject who experienced symptoms of TIO since childhood and for whom the causative tumor has not been identified. If available,
PHEX mutation analysis previously performed by a qualified laboratory as part of standard medical care may be used. The Investigator will communicate any genetic testing results to the subject.

7.5.6.2 Concomitant Medications/Therapies

Concomitant medications and therapies will be reviewed and recorded in the subject's CRF at each study visit to the investigational site, beginning at the initial Screening Visit. Concomitant medications and therapies will also be assessed during all Home Health visits and during the Safety Follow-up Period (described in Section 7.1). Medications (investigational, prescription, over-the-counter, and herbal) and nutritional supplements taken during the 30 days prior to Screening will be reviewed and recorded. Therapies (physical therapy, occupational therapy as well as mobility and walking devices, including ankle foot orthoses, braces, cane, crutches, walker, wheelchair etc.) utilized during the 30 days prior to Screening will also be reviewed and recorded. At each subsequent visit, change in medications and therapies since the previous visit will be recorded.

7.5.6.3 Adverse Events

All AEs will be recorded from the time the subject signs the informed consent through the EOS/ET Visit or Safety Follow-up TC (described in Section 7.1). The determination, evaluation, reporting, and follow-up of AEs will be performed as outlined in Section 8.5.
At each visit, subjects will be asked about any new or ongoing AEs since the previous visit. Assessments of AEs will occur at each visit to the investigational site and at all home health visits.

Clinically significant changes from Baseline in physical examination findings, vital signs, clinical laboratory parameters, renal ultrasounds, eGFR, ECHO and electrocardiograms (ECGs) will be recorded as AEs or SAEs, if appropriate.

7.5.6.4 Clinical Laboratory Tests for Safety

A comprehensive serum metabolic panel (Chem-20), complete blood count, and urinalysis will be used as routine screens to assess KRN23 safety. Certain analytes (ie, ALP and serum phosphorus) in the routine Chem-20 panel are also designated as PD/efficacy parameters in this study (Section 7.5.3). KRN23 biochemical parameters of interest include serum 25-hydroxyvitamin D, lipase, amylase, total calcium, creatinine, FGF23, iPTH; and urinary calcium and creatinine. Reflexive testing for serum amylase isoenzymes will be performed if serum amylase levels are elevated by ≥ 2x the upper limit of the reference range.

Blood and urine samples will be collected at Screening, Baseline, and regular intervals throughout the study as indicated in the Schedule of Events (Table 2.1, Table 2.2, Table 2.3, Table 2.4, Table 2.5, and Table 2.6). The local lab at the investigational site will be used to measure laboratory safety parameters required for study eligibility (except FGF23). Fasting for a minimum of 8 hours (overnight) is required prior to each blood draw; the duration of fasting will be recorded on the CRF. Twenty-four-hour urine collection is required to assess urinary phosphorus:creatinine and calcium:creatinine ratios; urinary phosphorus (a PD parameter, Section 7.5.3) will also be obtained from 24-hour urine samples.

Clinical laboratory parameters to be assessed for safety are provided in Table 7.5.6.4.1. See Study Reference Manual for details on sample collection and processing.

Table 7.5.6.4.1: Clinical Laboratory Assessments

Chemistry	Hematology	Urinalysis
25(OH) D	Hematocrit	Appearance
1,25(OH)$_2$ D	Hemoglobin	Color
Alanine aminotransferase	Platelet count	pH
Alkaline phosphatase[1]	Red blood cell count	Specific gravity
Amylase[2]	White blood cell count	Ketones
Amylase isoenzymes[2]	Mean corpuscular volume	Protein
Aspartate aminotransferase	Mean corpuscular hemoglobin	Glucose
Bilirubin (direct and total)	Mean corpuscular hemoglobin concentration	
Blood urea nitrogen		
Calcium (total)		
Chloride		
Carbon dioxide		**24-hour Urine**
Cholesterol (total)		Calcium
Creatinine		Creatinine
Gamma-glutamyl transpeptidase		Phosphorus/creatinine ratio
Glucose		
FGF23		
Intact parathyroid hormone		
Lactate dehydrogenase		
Lipase		**2-hour Urine**
Phosphorus[1]		Calcium/creatinine ratio
Potassium		Phosphorus
Protein (albumin and total)		Creatinine
Sodium		
Uric acid		**Pregnancy Test** (if applicable)

[1] Also designated as PD/efficacy parameter.

[2] Will be assessed reflexively if amylase levels are ≥ 2x the upper limit of the reference range.

Subjects who experience an SAE considered to be possibly or probably related to study drug or other AE of concern may, at the discretion of the Investigator (and/or Medical Monitor), have additional blood samples taken for safety laboratory tests.

7.5.6.5 Anti-KRN23 Antibody Screening

To determine the immunogenicity profile of KRN23, blood samples will be obtained for analysis of anti-KRN23 antibodies (anti-drug antibodies [ADA]) at predose and postdose time points as indicated in the Schedule of Events (Table 2.1, Table 2.2, Table 2.3, Table 2.4, Table 2.5, and Table 2.6). The concentration of anti-KRN23 antibodies in human serum will be determined using a validated solid phase extraction with acid dissociation assay and a 3-tiered strategy: screening assay and specificity confirmation assay followed by titer measurement. If the development of anti-KRN23 antibodies is suspected in a given subject, samples may be obtained at additional time points on a case-by-case basis, if warranted.

7.5.6.6 Renal Ultrasound and Glomerular Filtration Rate

Renal ultrasounds will be conducted at predose and postdose time points as indicated in the Schedule of Events (Table 2.1, Table 2.2, Table 2.3, Table 2.4, Table 2.5, and Table 2.6). Ultrasonographic findings of nephrocalcinosis will be graded on a 5-point scale (Verge et al. 1991). Central readings will be performed for all Screening and post-treatment renal ultrasounds to evaluate changes in calcifications and all other renal abnormalities from Baseline (ie, Screening assessment). Results obtained at Screening will serve as Baseline data. Central readers will be blinded to all other subject data.

The eGFR will be calculated via urine collections at predose and postdose time points as indicated in the Schedule of Events (Table 2.1, Table 2.2, Table 2.3, Table 2.4, Table 2.5, and Table 2.6). Results obtained at Screening will serve as Baseline data.

7.5.6.7 Echocardiogram

ECHO will be performed at predose and postdose time points as indicated in the Schedule of Events (Table 2.1, Table 2.2, Table 2.3, Table 2.4, Table 2.5, and Table 2.6). The goal is twofold: 1) assess for evidence of ectopic mineralization in the heart and aorta, and 2) evaluate for signs of left ventricular hypertrophy (LVH) or cardiac dysfunction. Additional tests may be performed if any abnormalities are detected or if medically indicated. ECHO administration procedures will be standardized and results will be read centrally by a central reading procedure to ensure consistent criteria across the study are used to assess ECHO parameters by trained personnel. Clinically significant abnormalities detected on ECHO will be promptly reported to the Principal Investigator for action as appropriate, including determining if an AE should be recorded in the CRF.

7.5.6.8 Electrocardiogram

A standardized 12-lead ECG will measure PR, QRS, QT, and corrected QT intervals (QTc) at predose and postdose time points as indicated in the Schedule of Events (Table 2.1, Table 2.2, Table 2.3, Table 2.4, Table 2.5, and Table 2.6). The goal is to evaluate both for LVH changes, as well as for changes in conductivity and intervals. ECG administration procedures will be standardized, and results will be read centrally by a single central reader. The ECG results will be assessed for any clinically significant abnormality or relevant changes from Baseline, and these will be recorded as AEs on the CRF.

7.5.6.9 Tumor Imaging

For subjects with TIO, if the tumor is visible and localized at Screening, it will be imaged at Screening and every 24 weeks through Week 144 and then every 48 weeks (or at next scheduled visit) for the remainder of the study (Table 2.1, Table 2.2, Table 2.3, Table 2.4, Table 2.5, and Table 2.6) using the same radiologic imaging technique (either CT scan or magnetic resonance imaging [MRI]) used initially to identify the tumor. Screening images will be used as Baseline. If a subject has a historical scan obtained within 12 months of Screening, the historical scan may be used as the Screening image. In cases where the tumor

was initially identified using another imaging technology, a Baseline CT scan or MRI (whichever is more clinically appropriate) will be performed. During follow-up, the same imaging technique (CT scan or MRI) that was used at Screening will be utilized to assess tumor size. The radiologist will indicate whether tumor size and volume have changed over time. If applicable, tumor imaging will be assessed using the mint Lesion™ software.

For subjects with TIO whose tumor was not identifiable at Screening, evaluations every 48 weeks (or in alignment with regularly scheduled clinic visits) will be performed beginning at Week 120 in an attempt to locate the tumor (Table 2.3, Table 2.4, Table 2.5, and Table 2.6). The preferred imaging technique is ^{68}Ga-DOTATATE positron emission tomography (PET)/CT. Octreotide-SPECT (SPECT/CT) or FDG-PT/CT may be used if ^{68}Ga-DOTATATE PET/CT is not available. (If none of these methods are available, another imaging technique may be used at Investigator discretion.) Imaging to locate the tumor may also be performed at unscheduled visits if there is clinical suspicion of new tumor development.

If the tumor is located and determined to be unresectable, MRI or CT imaging (at the discretion of the Investigator) should occur within 21 (± 7) days of identification to assess tumor size, at an unscheduled visit if necessary, and thereafter according to the Schedule of Events (Table 2.1, Table 2.2, Table 2.3, Table 2.4, Table 2.5, and Table 2.6). If the MRI/CT imaging following tumor identification has occurred within 3 months of the next scheduled MRI/CT assessment, that assessment will be skipped and the Schedule of Events will be followed thereafter. A subject whose tumor is identified and determined to be amenable to complete surgical resection may be removed from study (Section 7.3.3).

7.5.6.10 Vital Signs and Weight

Vital signs will include seated systolic blood pressure and diastolic blood pressure measured in millimeters of mercury (mm Hg), heart rate in beats per minute, respiration rate in breaths per minute, and temperature in degrees Celsius (°C). Vital signs measurements will be performed at predose and postdose time points as indicated in the Schedule of Events (Table 2.1, Table 2.2, Table 2.3, Table 2.4, Table 2.5, and Table 2.6) before any additional assessments are completed and after the subject has rested for 5 minutes. At each site visit, weight (in kilograms) will be obtained using a scale. Weight measurements will be used to calculate the appropriate KRN23 dose to be administered on a mg/kg basis.

7.5.6.11 Physical Examination

Complete physical examinations will be performed at predose and postdose time points as indicated in the Schedule of Events (Table 2.1, Table 2.2, Table 2.3, Table 2.4, Table 2.5, and Table 2.6). During the Treatment Extension Period, physical examinations will be performed at site visits only. Physical examinations will include assessments of general appearance; head, eyes, ears, nose, and throat; the cardiovascular, dermatologic, lymphatic, respiratory, gastrointestinal, genitourinary, musculoskeletal, and neurologic systems.

7.5.6.12 Pregnancy Testing and Contraception

Female subjects of childbearing potential will have urine pregnancy tests at the time points specified in the Schedule of Events (Table 2.1, Table 2.2, Table 2.3, Table 2.4, Table 2.5, and Table 2.6). Female subjects with a positive pregnancy test at Screening do not meet eligibility criteria for enrollment and will not be enrolled in the study.

Additional pregnancy tests will be performed at any visit in which pregnancy status is in question. A serum pregnancy test will be performed in the event of a positive or equivocal urine pregnancy test result, or can be performed if pregnancy test by urine is not feasible. Pregnancy in subject or partner must be reported (Section 8.5.4.5); pregnant subjects will be discontinued from the study.

Experience with KRN23 in pregnant women is limited. The study drug may involve risks to a pregnant female or unborn baby that are currently unknown.

Female participants of child-bearing potential who have not undergone a total hysterectomy or bilateral salpingo-oophorectomy and are sexually active must consent to use a highly effective method of contraception as listed below from the period following the signing of the informed consent through Safety Follow-up (as described in Section 7.1). Sexually active male participants with female partners of childbearing potential must consent to use a highly effective method of contraception listed below from the period following the signing of informed consent through 12 weeks after stopping the study drug. Examples of highly effective methods (CTFG 2014) include:

- Combined (estrogen and progestogen containing) hormonal contraception associated with inhibition of ovulation (eg, oral, intravaginal, transdermal)
- Progestogen-only hormonal contraception associated with inhibition of ovulation (eg, oral, injectable, implantable)
- Intrauterine device or intrauterine hormone-releasing system
- Bilateral tubal occlusion
- Male sterilization, also called vasectomy
- Sexual abstinence (ie, refraining from heterosexual intercourse during the entire period of risk associated with the study treatments, when this is in line with the preferred and usual lifestyle of the subject)

7.5.6.13 Dose Limiting Toxicity

A DLT is defined as the occurrence of any of the following:

- A Grade ≥ 3 toxicity that is probably or possibly related to the investigational product
- A confirmed serum phosphorus level of ≥ 6.5 mg/dL (defined as hyperphosphatemia) at any time after dosing

If a subject experiences a DLT, the planned dosing for that subject will be evaluated by the Sponsor. The outcome of this investigation will determine the subject's continuation or withdrawal from the study, as described in Section 7.3.3.1.

7.5.7 Appropriateness of Measurements

The assessments and timing of assessments used in this study and the variables analyzed are typical of those used to evaluate skeletal defects, hypophosphatemia, renal function, and vitamin D metabolism in patients with TIO and ENS-associated osteomalacia. The primary goal of treatment in TIO and ENS-associated osteomalacia patients with inoperable or incompletely resected tumors/lesions is to normalize serum phosphorus levels and correct or minimize osteomalacia, fractures, and bone and muscle pain.

Trans-iliac crest bone biopsies will be performed at Baseline and at Week 48 for assessment of bone histology and histomorphometry to evaluate tissue level changes in bone remodeling. By performing the procedure before and after treatment with KRN23, each subject will serve as an individual control.

The trans-iliac crest bone biopsy is a well-established tool in research and clinical use for evaluating bone tissue in metabolic bone diseases such as renal osteodystrophy, osteomalacia, and osteoporosis. Cellular activity and the amount of bone can be accurately quantified by histomorphometry, providing information that cannot be obtained through other imaging or biochemical studies (Sullivan et al. 1992). Osteomalacia is characterized by an accumulation of osteoid tissue, reflecting a prolonged and defective mineralization process (Arnstein et al. 1967). Specific histomorphometric parameters chosen to evaluate changes in osteomalacia with KRN23 treatment include osteoid thickness, osteoid surface/bone surface, and osteoid volume/bone volume, which are static parameters providing information about the amount of unmineralized bone (Dempster et al. 2013).
In addition, mineralization lag time, a dynamic modeling parameter representing the mean time interval between the formation of osteoid and its subsequent mineralization, will be evaluated (Dempster et al. 2013). In TIO, surgical resection of the tumor has been shown to lead to complete resolution of osteomalacia by quantitative histomorphometry on bone biopsy (Shane et al. 1997).

Hypophosphatemia is the cause of the consequent osteomalacia and other symptoms that result in clinical manifestations of TIO and ENS-associated osteomalacia. Serum phosphorus will be measured throughout the study to evaluate changes in response to KRN23 treatment.

FGF23 plays an important role in phosphate homeostasis, and KRN23 binds to and inhibits FGF23; serum FGF23 levels will be monitored throughout the study.

Additional assessments are included both as PD and safety indicators of potential secondary complications associated with treatment, including serum calcium, 1,25(OH)$_2$D, and urinary calcium and creatinine, as hypercalciuria may occur in the absence of hypercalcemia.
Intact PTH levels and TmP/GFR are routinely measured as a part of medical management of TIO. Biomarkers of bone formation (P1NP, ALP, BALP, osteocalcin) and resorption (CTx) may provide an indication of treatment effect. The relatively extensive panel of biomarkers has been included in this study to provide the most information on relevant clinical laboratory parameters for endpoint confirmation and analysis. Where possible, timing of assessments has been coordinated with standard safety laboratory tests to minimize risk and discomfort and avoid unnecessary duplication of testing.

Radiographs, DXA, and XtremeCT will be used to evaluate healing of osteomalacia, prior fractures, and bone mineral density and bone mineral content. HHD, STS, WAL, and 6WMT will be used as performance measures and have been successfully used in other clinical development programs with similar diseases. BPI will be used as an assessment of overall pain. BFI will be used as an assessment of general fatigue. The SF-36 was included to assess physical and mental function and health-related quality of life.

The safety parameters to be evaluated in this study include standard assessments such as recording of medical history, AEs and SAEs, physical examination, vital signs, serum chemistry, concomitant medications, and other routine clinical and laboratory procedures. Routine, non-invasive procedures will provide relevant indicators of possible renal and cardiac risk; renal ultrasounds and ECHO will be used to detect any calcinosis in susceptible organs. Tumor imaging will be used to assess any changes in localized tumor size in subjects with TIO. Since elevated free FGF23 has been associated with LVH in patients with chronic kidney disease, ECHO and ECGs will examine the potential risk in TIO and ENS-associated osteomalacia subjects.

7.6 Statistical Methods and Determination of Sample Size

The completeness of the data affects the integrity and accuracy of the final study analysis. Therefore, every effort will be made to ensure complete, accurate, and timely data collection, and to avoid missing data. The procedures for handling missing, spurious, or unused data, and the detailed method for analyses will be presented in the Statistical Analysis Plan (SAP); the information below is intended as a guide to planned analyses.

7.6.1 Efficacy Endpoints

All efficacy parameters will be summarized at Baseline and at each observed time that they are collected.

7.6.1.1 Primary Efficacy Endpoints

The study will have co-primary endpoints:

- The proportion of subjects achieving mean serum phosphorus levels above the lower limit of normal (LLN; 2.5 mg/dL [0.81 mmol/L]) at the mid-point of the dose interval (2 weeks after dosing), as averaged across dose cycles between Baseline and Week 24
- The change from Baseline in excess osteoid based on analysis of iliac crest bone biopsies after 48 weeks of KRN23 treatment using the following histomorphometric indices:
    - O.Th
    - OS/BS
    - OV/BV
    - MLt

7.6.1.2 Secondary Endpoints

- Additional measures to assess serum phosphorus levels over time include:
    - Proportion of subjects achieving mean serum phosphorus levels above the LLN (2.5 mg/dL [0.81 mmol/L]) at the end of the dosing cycle (4 weeks after dosing), as averaged across dose cycles between Baseline and Week 24
    - Mid-point of dosing cycle: mean change from Baseline and percent change from Baseline averaged across dose cycles between Baseline and Week 24
    - End of dosing cycle: mean change from Baseline averaged across dose cycles between Baseline and Week 24
    - Cumulative exposure: time-adjusted area under the curve between Baseline and Week 24
- Change from Baseline over time in serum FGF23, ALP, 1,25$(OH)_2$D; and urinary phosphorus, TRP, TmP/GFR
- Change and percent change from Baseline over time in serum biochemical markers of bone turnover, including BALP, CTx, P1NP, and osteocalcin
- Change from Baseline over time in muscle strength as assessed by HHD, STS test, WAL test, and 6MWT
- Change from Baseline in BPI, BFI, and SF-36 over time

7.6.1.3 Exploratory Endpoints

- Changes in other measures of structural and dynamic histomorphometry in trans-iliac crest bone biopsies
- Changes in bone condition and healing of prior long bone and pseudo-fractures as assessed by ^{99m}Tc-labelled bone scan
- Changes in bone mineral density and bone mineral content as measured by DXA at the lumbar spine and hip
- Changes in bone mineral density, bone mineral content, bone geometry, and microarchitecture in the cortical and trabecular compartments of the radius and tibia as measured by XtremeCT (when available)

7.6.2 Statistical and Analytical Plans

7.6.2.1 Analysis Populations

Biopsy Analysis Set: The biopsy analysis set will include enrolled subjects with Baseline and follow-up (either Week 48 or ET prior to Week 48) bone biopsy data.

Full Analysis Set: All efficacy (except bone biopsy endpoints), safety, and PK/PD analyses will be performed on the set of all subjects who receive at least 1 dose of investigational product.

7.6.2.2 Statistical Principles

Descriptive statistics will be used to summarize the data. For continuous variables, the mean, the standard deviation, median, minimum, and maximum will be provided. For discrete data, the frequency and percent distributions will be provided. Statistical tests will use 2-sided alpha = 0.05 significance level. Two-sided 95% confidence intervals will also be presented.

Analyses of the data will be performed when the first 3 subjects have completed their Week 12 visit. An additional analysis will occur using all available data when the first subject completes the post-treatment bone biopsy and may occur at other time points. The final analysis will be performed at the end of the study.

7.6.2.3 Demography, Baseline Characteristics, and Disposition

Demographics (age, sex, and race) and other baseline disease characteristics will be summarized using descriptive statistics for the Safety Analysis Set.

The number of subjects screened, enrolled, treated, and completed will be summarized. Subject discontinuation from the study, and from treatment, will each be summarized including reason for discontinuation.

For parameters/assessments scheduled to be performed on the same day as the first study treatment, the Baseline value is the last value measured before the first administration of study treatment on that day. For parameters/assessments not scheduled to be performed (or scheduled but not performed) on the same day as the first administration of study treatment, the Baseline value is the value from the Screening Period measured closest to the day of first administration of study treatment.

7.6.2.4 Efficacy Analyses

7.6.2.4.1 Primary Efficacy Analysis

The proportion of subjects who achieve a serum phosphorus level above the LLN (2.5 mg/dL [0.81 mmol/L]), at 2 weeks after dosing (between baseline and Week 24, on average) will be provided, along with the 2-sided 95% confidence interval. Additional analyses of serum phosphorus including observed values, change from Baseline, percent change from Baseline over time, and area under the curve will be summarized from Baseline to Week 24.

Histomorphometric indices O.Th, OS/BS, OV/BV, and MLt at Baseline and Week 48, and their change from Baseline at Week 48 will be summarized.

7.6.2.4.2 Secondary and Exploratory Efficacy Analyses

Secondary and exploratory efficacy parameters will be summarized at Baseline and at each time point they are collected. The changes from Baseline (absolute and percent) will be described across subjects in terms of the mean and standard deviation and may be tested using the generalized estimating equation method, if the model converges. The models for change from Baseline (absolute and percent) will include Baseline, and time (weeks of treatment) as a categorical variable.

Correlations among PD parameters and efficacy endpoints may also be assessed.

The number and percent of subjects with postdose serum phosphorus levels will be summarized by the following ranges:

- ≤ 2.5 mg/dL (0.81 mmol/L)
- > 2.5 mg/dL (0.81 mmol/L) but ≤ 4.0 mg/dL (1.13 mmol/L)
- > 4.0 mg/dL (1.13 mmol/L)

Individual PD-time plots may be presented for each subject as well as mean PD-time plots. Additional analyses using statistical models may be performed. The relationship between various PD parameters as well as KRN23 concentrations may be examined.

7.6.2.5 Safety Analyses

All subjects in the Safety Analysis Data Set will be included in all summaries of safety endpoints.

The safety measures in this study are:

- Incidence and frequency of AEs, treatment-related AEs, and SAEs
- Clinically significant changes from Baseline in vital signs, weight, physical examination findings, and clinical laboratory tests
- ECHO, ECG, and renal ultrasound
- Tumor imaging or dermatologic assessment of skin lesions
- Concomitant medications
- Anti-KRN23 antibodies

7.6.2.5.1 Adverse Events

All AEs will be coded using the Medical Dictionary for Regulatory Activities (MedDRA). The incidence and frequency of AEs will be summarized by System Organ Class, Preferred Term (PT), relationship to study drug, and severity. All reported AEs with onset during the treatment (ie, treatment-emergent AEs) will be included in the analysis. For each AE, the percentage of subjects who experienced at least 1 occurrence of the given event will be summarized. The numbers (frequency) and incidence rates of AEs and SAEs will be summarized. Special attention will be given to those subjects who died, discontinued treatment because of an AE, or experienced a SAE (eg, summaries, listings, and narrative preparation may be provided, as appropriate).

7.6.2.5.2 Clinical Laboratory Evaluations

Clinical laboratory data will be summarized by the type of laboratory test. Reference ranges and markedly abnormal results (specified in the SAP) will be used in the summary of laboratory data. Descriptive statistics will be calculated for each laboratory analyte at Baseline and at each scheduled time point. Changes from Baseline results will be presented in pre- versus post-treatment cross tabulations (with classes for below, within, and above normal ranges). A listing of subjects with any markedly abnormal laboratory results will be provided. The frequency and percentage of subjects who experience abnormal clinical laboratory results (ie, outside of reference ranges) and/or clinically significant abnormalities will be presented for each clinical laboratory measurement. A list of reference ranges standardized across clinical laboratories will be provided.

7.6.2.5.3 Vital Signs

Vital signs will be summarized and listed by individual subject. Summaries of vital signs data over time and changes from Baseline over time will be provided.

7.6.2.5.4 Echocardiograms and Electrocardiograms

Echocardiogram and ECG data will be listed and summarized by individual subject and study population. Individual subject results will be examined in relation to Baseline recordings. Summaries of PR, QRS, QT and QTc intervals over time and changes from Baseline over time will be provided.

7.6.2.5.5 Renal Ultrasound

Renal ultrasound data will be listed and summarized for each subject and study population.

7.6.2.5.6 Prior and Concomitant Medications

The World Health Organization drug dictionary will be used to classify prior and concomitant medications by therapeutic class and PT. Prior medications include medications that were taken before the first administration of study drug, including those reported before dosing at the Baseline (Day 0) study visit. Concomitant medications include medications that were taken at any time after the start of treatment within this study until the End-of-Treatment visit.

7.6.2.6 Exposure

Exposure to KRN23 will be summarized using the number of doses and total amount of KRN23 administered to each subject during the study.

7.6.2.7 Anti-KRN23 Antibody Assessment

The anti-KRN23 antibody data, including ADA titer and neutralizing activity, will be listed and summarized by individual subject and study population.

7.6.3 Determination of Sample Size

Assuming the proportion of subjects with a phosphorus level above the lower limit of normal at 2 weeks after dosing, as averaged across dose cycles between Baseline and Week 24 is 60%, a sample size of 15 subjects will provide a 95% confidence interval with the half width no greater than 24.8%. A reduction in excess osteoid is expected to be shown in all subjects with paired biopsies, with an estimated ≥ 50% reduction from Baseline in osteoid thickness. The sample size and study duration are believed to be sufficient to enable characterization of KRN23 effects on serum phosphorus levels, excess osteoid histomorphometric indices, and the safety profile of KRN23.

8 STUDY CONDUCT

8.1 Ethics

8.1.1 Institutional Review Board

The IRB must be a properly constituted board or committee operating in accordance with 21 Code of Federal Regulations (CFR) Part 56, “Institutional Review Boards.” This protocol, any protocol amendments, the associated Informed Consent Forms (ICFs), and the informed consent procedures must be submitted to the IRB for review and must be approved before the enrollment of any subject into the study. Investigational product may not be shipped to the Investigator until Ultragenyx or its designee has received a copy of the letter or certificate of approval from the IRB for the protocol and any protocol amendments, as applicable.

All subject recruitment and/or advertising information must be submitted to the IRB and Ultragenyx or its designee for review and approval prior to implementation. IRB approval of any protocol amendments must be received before any of the changes outlined in the amendments are put into effect, except when the amendment has been enacted to protect subject safety. In such cases, the chair of the IRB should be notified immediately and the amendment forwarded to the IRB for review and approval.

8.1.2 Ethical Conduct of Study

This protocol is written in accordance with the principles established by the 18th World Medical Assembly General Assembly (Helsinki, 1964) and subsequent amendments and clarifications adopted by the General Assemblies. The Investigator will make every effort that the study described in this protocol is conducted in full conformance with those principles, current FDA regulations, International Conference on Harmonisation of Technical Requirements for Registration of Pharmaceuticals for Human Use (ICH) Good Clinical Practice (GCP) guidelines, and local ethical and regulatory requirements. Should a conflict arise, the Investigator will follow whichever law or guideline affords the greater protection to the individual subject. The Investigator will also make sure he or she is thoroughly familiar with the appropriate administration and potential risks of administration of the investigational product, as described in this protocol and the Investigator’s Brochure, prior to the initiation of the study.

8.1.3 Subject Information and Consent

Appropriate forms for documenting written informed consent will be provided by the Investigator and reviewed and approved by Ultragenyx or its designee before submission to the IRB. Ultragenyx or its designee must receive a copy of the IRB’s approval of the ICF before the shipment of investigational product to the study site.

It is the Investigator’s responsibility to obtain signed written informed consent from each potential study subject prior to the conduct of any study procedures. This written informed consent will be obtained after the methods, objectives, requirements, and potential risks of

the study have been fully explained to each potential subject. The Investigator must explain to each subject that the subject is completely free to refuse to enter the study or to withdraw from it at any time.

The method of obtaining and documenting informed consent and the contents of the ICF will comply with ICH GCP guidelines, the requirements of 21 CFR Part 50, “Protection of Human Subjects,” the Health Insurance Portability and Accountability Act (HIPAA) regulations, and all other applicable regulatory requirements. Subjects will be given a copy of the signed ICF and will be provided any new information during the course of the study that might affect their continued participation in the study. The Investigator or a qualified designee will be available to answer each subject's questions throughout the study, and all of the subject’s questions must be answered to the subject’s satisfaction. If the protocol is amended and the ICF is revised, each subject will be required to provide written informed consent again using the revised ICF.

Receipt of written informed consent will be documented in each potential subject’s CRF. The signed ICF will remain in each subject's study file and must be available to the Study Monitor(s) at all times.

8.2 Investigators and Study Administrative Structure

Each Investigator must provide Ultragenyx and/or its designee a completed and signed Form FDA 1572 and a Financial Disclosure Form. All subinvestigators must be listed on Form FDA 1572 and Financial Disclosure Forms must be completed for all subinvestigators listed on Form FDA 1572.

Ultragenyx and/or its designee will be responsible for managing and monitoring the clinical trial to ensure compliance with FDA and ICH GCP guidelines. Ultragenyx's trained designated representative (the Monitor) will conduct regular visits to the clinical site, to perform source document verification. The Monitor will verify the Investigator's ongoing qualifications, to inspect clinical site facilities, and to inspect study records, including proof of IRB review, with the stipulation that subject confidentiality will be maintained in accordance with local and federal regulations, including HIPAA requirements.

A Coordinating Investigator will be identified for multicenter trials. The Coordinating Investigator will be selected on the basis of active participation in the trial, thorough knowledge of the therapeutic area being studied, and the ability to interpret data.
The Coordinating Investigator will read and sign the Clinical Study Report in accordance with regulatory authority requirements.

8.3 Investigational Product Accountability

While at the clinical site, investigational product must be stored in a secure limited access location at controlled temperature as described in the Investigator’s Brochure and according to product packaging. The storage facility must be available for inspection by the study monitor at any time during the study.

A drug accountability record must be maintained for all investigational product received, dispensed, returned, and/or lost during the study. This record must be kept current and made available to the Study Monitor for inspection. Following the close-out of the study, all unused investigational product must be returned to Ultragenyx and/or its designee unless other instructions have been provided for final disposition of the investigational product.

8.4 Data Handling and Record Keeping

8.4.1 Case Report Forms and Source Documents

The Investigator is required to initiate and maintain, for each subject, an adequate and accurate case history that records all observations and other data related to the study for that subject. A validated Electronic Data Capture (EDC) system will be used for entry of the data into electronic CRFs. Data must be recorded on CRFs approved by Ultragenyx or its designee. All information recorded on CRFs for this study must be consistent with the subject's source documentation.

Initial data entry and any changes to the data will be made only by Ultragenyx-authorized users, and data entries and changes will be captured in an electronic audit trail. An explanation of any data change should be recorded in the CRF. All data entered in to the CRF must be verifiable; therefore, CRFs will be routinely checked for accuracy, completeness, and clarity and will be cross-checked for consistency with source documents, including laboratory test reports and other subject records by Ultragenyx or its designee. The Investigator must allow direct access to all source documents.

8.4.2 Data Quality Assurance

Monitoring and auditing procedures developed by Ultragenyx and/or its designee will be implemented to ensure compliance with FDA and ICH GCP guidelines. Ultragenyx's designated representative (the Monitor) will contact the Investigator and conduct regular visits to the study site. The Monitor will be expected and allowed to verify the Investigator's qualifications, to inspect clinical site facilities, and to inspect study records, including proof of IRB review, with the stipulation that subject confidentiality will be maintained in accordance with local and federal regulations, including HIPAA requirements. The Monitor will also be responsible for confirming adherence to the study protocol, inspecting CRFs and source documents, and ensuring the integrity of the data. CRFs will be checked for accuracy, completeness, and clarity and will be cross-checked for consistency with source documents, including laboratory test reports and other subject records. Instances of missing or uninterpretable data will be resolved in coordination with the Investigator.

The Monitor will also investigate any questions concerning adherence to regulatory requirements. Any administrative concerns will be clarified and followed. The Monitor will maintain contact with the site through frequent direct communications with the study site by e-mail, telephone, facsimile, and/or mail. The Investigator and all other site personnel agree

to cooperate fully with the Monitor and will work in good faith with the Monitor to resolve any and all questions raised and any and all issues identified by the Monitor.

The Investigator understands that regulatory authorities, the IRB, and/or Ultragenyx or its designees have the right to access all CRFs, source documents, and other study documentation for on-site audit or inspection and will retain this right from the start of the study to at least 25 years after the end of the clinical trial or in accordance with national law. The Investigator is required to guaranty access to these documents and to cooperate with and support such audits and inspections.

8.4.3 Record Retention

All study records must be retained for at least 25 years after the end of the clinical trial or in accordance with national law. Subject files and other source data must be kept for the maximum period of time permitted by the hospital, institution, or private practice, but not less than 25 years. Ultragenyx must be notified should the Investigator/institution be unable to continue maintenance of subject files for the full 25 years. All study records must be stored in a secure and safe facility.

8.5 Reporting and Follow-up of Adverse Events

8.5.1 Definition of Adverse Events

An AE is defined as any untoward medical occurrence associated with the use of a drug in humans, whether or not considered drug-related. An AE can therefore be any unfavorable and unintended sign (including an abnormal laboratory finding, for example), symptom, or disease temporally associated with the use of a medicinal (investigational) product, whether or not related to the medicinal (investigational) products.

All AEs will be collected from the time the subject signs informed consent through up to 12 weeks (approximately 5 times the elimination half-life) following the last dose of study drug (unless the subject starts treatment with commercial KRN23 [burosumab], see Section 7.1). In addition, the Investigator should report any AE that occurs after this time period that is believed to have a reasonable possibility of being associated with study drug.

AEs ongoing at 12 weeks following the last dose of study drug should have a comment in the source document by the Investigator whether the event has recovered, recovered with sequelae, or stabilized.

Suspected Adverse Reaction is any AE for which there is a reasonable possibility that the drug caused the AE. For the purposes of expedited safety reporting, “reasonable possibility” means there is evidence to suggest a causal relationship between the drug and the AE. Suspected adverse reaction implies a lesser degree of certainty about causality than adverse reaction, which means any AE caused by a drug.

Life-threatening AE or life-threatening suspected adverse reaction is an AE or suspected adverse reaction that, in the view of either the Investigator or Sponsor, places the subject at immediate risk of death. It does not include an AE or suspected adverse reaction that, had it occurred in a more severe form, might have caused death.

SAE or serious suspected adverse reaction is an AE or suspected adverse reaction that at any dose, in the view of either the Investigator or Sponsor, results in any of the following outcomes:

- Death
- A life-threatening AE
- Inpatient hospitalization or prolongation of existing hospitalization
- Persistent or significant incapacity or disability (substantial disruption of the ability to conduct normal life functions)
- A congenital anomaly/birth defect

Note that hospitalizations planned prior to study enrollment (eg, for elective surgeries) are not considered SAEs. Hospitalizations that occur for pre-existing conditions that are scheduled after study enrollment are considered SAEs.

Important medical events that may not result in death, be immediately life-threatening, or require hospitalization may be considered serious when, based upon appropriate medical judgment, they may jeopardize the subject and may require medical or surgical intervention to prevent 1 of the outcomes listed in the definition.

8.5.2 Severity of Adverse Events

Wherever possible, the severity of all AEs will be graded using the Common Terminology Criteria for Adverse Events (CTCAE) version 4.0. The majority of AEs can be graded using this system. If an AE cannot be graded using the CTCAE criteria, it should be graded as mild, moderate, severe, life-threatening, or death using the following definitions:

- ***Mild (Grade 1):*** Awareness of signs or symptoms, but easily tolerated and are of a minor irritant type, causing no loss of time from normal activities. Symptoms do not require therapy or a medical evaluation; signs and symptoms are transient
- ***Moderate (Grade 2):*** Events introduce a low level of inconvenience or concern to the subject and may interfere with daily activities, but are usually improved by simple therapeutic measures; moderate experiences may cause some interference with functioning
- ***Severe (Grade 3):*** Events interrupt the subject's normal daily activities and generally require systemic drug therapy or other treatment; they are usually incapacitating

- ***Life-threatening (Grade 4):*** Events that place the subject at immediate risk of death or are disabling
- ***Death (Grade 5):*** Events that result in death

To make sure there is no confusion or misunderstanding of the difference between the terms "serious" and "severe," which are not synonymous, the following note of clarification is provided. The term "severe" is often used to describe the intensity (severity) of a specific event (as in mild, moderate, or severe myocardial infarction); the event itself, however, may be of relatively minor medical significance (such as severe headache). This is not the same as "serious" which is based on subject/event outcome or action criteria usually associated with events that pose a threat to a subject's life or functioning. Seriousness (not severity) serves as a guide for defining regulatory reporting obligations.

8.5.3 Relationship of Adverse Events to Study Drug

The Investigator will assess the potential relationship of the AE to study drug using the following descriptions. For the purposes of reporting to regulatory agencies, AEs deemed as Definitely, Probably, or Possibly Related will be considered Related and those deemed Definitely Not or Probably Not Related will be considered Unrelated.

Categories of attributions for "Not Related" events:

- ***Definitely Not Related***: This category applies to an AE that ***is clearly not related*** to the investigational agent/procedure, beyond a reasonable doubt. That is, another cause of the event is most plausible; and/or a clinically plausible temporal sequence is inconsistent with the onset of the event and the exposure to study drug and/or a causal relationship is considered biologically implausible
- ***Probably Not Related:*** This category applied to an AE that ***is doubtfully related*** to the investigational agent/procedure. That is, an alternative explanation is more likely, eg, concomitant drug(s), concomitant disease(s), known consequences of the disease under investigation or the relationship in time suggest that a causal relationship is unlikely

Categories of attributions for "Related" events:

- ***Possibly Related:*** This category applies to an AE that ***may be related*** to the investigational agent/procedure. That is the AE follows a reasonable temporal sequence from administration of the study drug and that follows a known or expected response pattern to the suspected study drug, but that could readily have been produced by a number of other factors
- ***Probably Related:*** This category applies to an AE that ***is likely related*** to the investigational agent/procedure. That is, the AE has a temporal relationship to the administration of the investigational agent(s) or research intervention, follows a known or suspected pattern of response, and is strongly associated with study drug exposure.

An alternative explanation is less likely, eg, concomitant drugs(s), concomitant medication(s)

- ***Definitely Related:*** This category applies to an AE that ***is clearly related*** to the investigational agent/procedure. That is, the AE is listed as a possible adverse reaction and cannot be reasonably explained by an alternative explanation, eg, concomitant drug(s), concomitant disease(s), known consequences of the disease under investigation or the relationship in time is very suggestive (eg, it is confirmed by dechallenge and rechallenge)

8.5.4 Adverse Event Reporting to Ultragenyx

8.5.4.1 General

All AEs (ie, any new or worsening in severity or frequency of a preexisting condition) with onset after the subject signs consent for study participation must be promptly documented on the AE eCRF via the EDC system. The Investigator is responsible for evaluating all AEs, obtaining supporting documents, and ensuring documentation of the event is adequate. Details of the AE must include severity, relationship to study drug, duration, and outcome.

All AEs will be collected from the time the subject signs informed consent through their EOS/ET Visit or final Safety Follow-up TC, as defined in Section 7.1. In addition, the Investigator should report any AE that occurs after this time period that is believed to have a reasonable possibility of being associated with study drug.

AEs ongoing at a subject's EOS/ET Visit or final Safety Follow-up TC (Section 7.1) should have a comment in the source document by the Investigator whether the event has recovered, recovered with sequelae, or stabilized.

8.5.4.2 Serious Adverse Events, Serious Adverse Drug Reactions, and Requirements for Immediate Reporting

Any SAE that occurs at any time during the study, including a clinically significantly abnormal laboratory test result that is considered serious, must be reported within 24 hours of knowledge of the event to Ultragenyx or its designee. These requirements apply equally to all subjects, regardless of the study phase or the at-risk subject's treatment assignment or dosage. The reporting requirement for SAEs is from the time of signing of the ICF through the EOS/ET Visit or final Safety Follow-up TC, as defined in Section 7.1.

SAEs will be reported by completing and submitting SAE report forms to Ultragenyx or its designee. Initial SAE reports must be followed by detailed descriptions. These should include copies of hospital case records and other documents when requested. Telephone reports must be confirmed promptly by facsimile. Follow-up SAE information must be submitted in a timely manner as additional information becomes available. All SAEs regardless of relationship to study drug must be followed to resolution or stabilization if improvement is not expected.

Any death occurring from signing of the ICF through the EOS/ET Visit or final Safety Follow-up TC (Section 7.1) must be reported to Ultragenyx or its designee within 24 hours of knowledge of the death, whether or not it is considered treatment-related.

The Investigator also must notify the IRB/EC of the occurrence of the SAE, in writing, as soon as is practicable and in accordance with IRB/EC requirements and local law. A copy of this notification must be provided to Ultragenyx or its designee.

8.5.4.3 Urgent Safety Reporting

The regulations governing clinical studies state that the Sponsor and Investigator are required to take appropriate urgent safety measures to protect subjects against any immediate hazards that may affect the safety of subjects, and that the appropriate regulatory bodies should be notified according to their respective regulations. According to the European Union (EU) Clinical Trial Directive 2001/20/EC, "…in the light of the circumstances, notably the occurrence of any new event relating to the conduct of the trial or the development of the investigational medicinal product where that new event is likely to affect the safety of the subjects, the Sponsor and the Investigator shall take appropriate urgent safety measures to protect the subjects against any immediate hazard. The Sponsor shall forthwith inform the competent authorities of those new events and the measures taken and shall ensure that the Ethics Committee (EC) is notified at the same time." The reporting period for urgent safety issues is the period from the signing of the ICF through the EOS/ET Visit or final Safety Follow-up TC, as defined in Section 7.1. Investigators are required to report any urgent safety measures to Ultragenyx within 24 hours.

8.5.4.4 Adverse Drug Reaction Reporting

Ultragenyx or its designee will submit suspected unexpected serious adverse reactions (SUSAR) to appropriate Regulatory Authorities (including Competent Authorities in all Member States concerned), Ethics Committees, and Investigators as per local laws and regulations. Fatal and life-threatening SUSARs will be submitted no later than 7-calendar days of first knowledge of the event and follow-up information submitted within an additional 8 days. All other SUSARs will be submitted within 15-calendar days of first knowledge of the event.

Principal Investigators are required to report any urgent safety matters to Ultragenyx or its designee within 24 hours. Ultragenyx or its designee will inform the Regulatory Authorities, Ethics Committees, and Investigators of any events (eg, change to the safety profile of KRN23, major safety findings) that may occur during the clinical trial that do not fall within the definition of a SUSAR but may affect the safety of subjects participating in the clinical trials, as required, in accordance with applicable laws and regulations. The reporting period for urgent safety issues is the period from the signing of the ICF through the EOS/ET Visit or final Safety Follow-up TC, as defined in Section 7.1.

The Investigator will notify the IRBs/Research Ethics Boards (REB)/ECs of SAEs and urgent safety matters, in accordance with IRB/REB/EC requirements and local laws and regulations. A copy of this notification must be provided to Ultragenyx or its designee.

Non-SUSARs will be maintained in the Ultragenyx safety database and provided in annual and/or periodic reports as per local laws and regulations. Ultragenyx or its designee will prepare and submit annual safety reports and/or other aggregate periodic summary reports to Regulatory Authorities and Ethics Committees, as per local laws and regulations.

8.5.4.5 Pregnancy in Subject or Partner

Pregnancies in subjects or partners must be reported within 24 hours of knowledge of the event to Ultragenyx or its designee. The reporting period for pregnancies is the period from the signing of the ICF through the EOS/ET Visit or final Safety Follow-up TC, as defined in Section 7.1. Reported pregnancy of a subject or a subject's partner, while participating in the study, will be monitored for the full duration and/or followed until the outcome of the pregnancy is known. In the event of a pregnancy in the partner of a subject, the Investigator should make every effort to obtain the female partner's consent for release of protected health information. Refer to the Study Reference Manual for details on the reporting procedures to follow in the event of pregnancy. Pregnancy-associated SAEs will be processed and submitted, as necessary, as per the SUSAR reporting process (Section 8.5.4.4).

8.5.4.6 Safety Contact Information

Drug Safety	Medical Monitor
PrimeVigilance<br>Fax: PPD<br>e-mail: PPD	Mary Scott Roberts, MD<br>Telephone: PPD<br>Mobile: PPD<br>e-mail: PPD

8.6 Financing and Insurance

Financing and insurance for this clinical trial will be addressed in clinical trial agreements with the study site.

8.7 Publication Policy

Any publication or presentation by the Investigator and/or the Institution based on data or results resulting from the Ultragenyx study shall only be done in strict accordance with the Publication section in the Clinical Trial Agreement executed between Ultragenyx or its designee and the Institution and/or the Investigator.

9 REFERENCES

Aono, Y, Hasegawa, H, Yamazaki, Y, Shimada, T, Fujita, T, Yamashita, T, and Fukumoto, S. 2011. "Anti-FGF-23 neutralizing antibodies ameliorate muscle weakness and decreased spontaneous movement of Hyp mice." *J Bone Miner Res* 26 (4):803-10.

Aono, Y, Yamazaki, Y, Yasutake, J, Kawata, T, Hasegawa, H, Urakawa, I, Fujita, T, Wada, M, Yamashita, T, Fukumoto, S, and Shimada, T. 2009. "Therapeutic effects of anti-FGF23 antibodies in hypophosphatemic rickets/osteomalacia." *J Bone Miner Res* 24 (11):1879-88.

Arnstein, AR, Frame, B, and Frost, HM. 1967. "Recent progress in osteomalacia and rickets." *Ann Intern Med* 67 (6):1296-1330.

ATS. 2002. "American Thoracic Society Statement: Guidelines for the six-minute walk test." *Am.J.Respir, Crit.Care Med* 166 (1):111-117.

Bohannon, RW. 1997. "Reference values for extremity muscle strength obtained by hand-held dynamometry from adults aged 20 to 79 years." *Arch.Phys.Med.Rehabil.* 78 (1):26-32.

CTFG. 2014. Clinical Trial Facilitation Group. Recommendations related to contraception and pregnancy testing in clinical trials. http://www.hma.eu/fileadmin/dateien/Human_Medicines/01-About_HMA/Working_Groups/CTFG/2014_09_HMA_CTFG_Contraception.pdf.

Dempster, DW, Compston, JE, Drezner, MK, Glorieux, FH, Kanis, JA, Malluche, H, Meunier, PJ, Ott, SM, Recker, RR, and Parfitt, AM. 2013. "Standardized nomenclature, symbols, and units for bone histomorphometry: a 2012 update of the report of the ASBMR Histomorphometry Nomenclature Committee." *J Bone Miner Res* 28 (1):2-17.

Drezner, MK. 1999. "Tumor-induced osteomalacia." *Favus MJ (ed.)* Primer on Metabolic Bone Diseases and Disorders of Mineral Metabolism, 4th Edition:331-337.

Folpe, AL, Fanburg-Smith, JC, Billings, SD, Bisceglia, M, Bertoni, F, Cho, JY, Econs, MJ, Inwards, CY, Jan de Beur, SM, Mentzel, T, Montgomery, E, Michal, M, Miettinen, M, Mills, SE, Reith, JD, O'Connell, JX, Rosenberg, AE, Rubin, BP, Sweet, DE, Vinh, TN, Wold, LE, Wehrli, BM, White, KE, Zaino, RJ, and Weiss, SW. 2004. "Most osteomalacia-associated mesenchymal tumors are a single histopathologic entity: an analysis of 32 cases and a comprehensive review of the literature." *Am J Surg Pathol* 28 (1):1-30.

Fukumoto, S. 2008. "Physiological regulation and disorders of phosphate metabolism--pivotal role of fibroblast growth factor 23." *Intern Med* 47 (5):337-43.

Geller, JL, Khosravi, A, Kelly, MH, Riminucci, M, Adams, JS, and Collins, MT. 2007. "Cinacalcet in the management of tumor-induced osteomalacia." *J Bone Miner Res* 22 (6):931-7.

Gibbons, WJ, Fruchter, N, Sloan, S, and Levy, RD. 2001. "Reference values for a multiple repetition 6-minute walk test in healthy adults older than 20 years." *J Cardiopulm.Rehabil.* 21 (2):87-93.

Jan de Beur, SM, Streeten, EA, Civelek, AC, McCarthy, EF, Uribe, L, Marx, SJ, Onobrakpeya, O, Raisz, LG, Watts, NB, Sharon, M, and Levine, MA. 2002. "Localisation of mesenchymal tumours by somatostatin receptor imaging." *Lancet* 359 (9308):761-3.

Lim, YH, Ovejero, D, Sugarman, JS, Deklotz, CM, Maruri, A, Eichenfield, LF, Kelley, PK, Juppner, H, Gottschalk, M, Tifft, CJ, Gafni, RI, Boyce, AM, Cowen, EW, Bhattacharyya, N, Guthrie, LC, Gahl, WA, Golas, G, Loring, EC, Overton, JD, Mane, SM, Lifton, RP, Levy, ML, Collins, MT, and Choate, KA. 2014. "Multilineage somatic activating mutations in HRAS and NRAS cause mosaic cutaneous and skeletal lesions, elevated FGF23 and hypophosphatemia." *Hum Mol Genet* 23 (2):397-407.

Olivares, JL, Ramos, FJ, Carapeto, FJ, and Bueno, M. 1999. "Epidermal naevus syndrome and hypophosphataemic rickets: description of a patient with central nervous system anomalies and review of the literature." *Eur J Pediatr* 158 (2):103-7.

Peters, MJ, van Nes, SI, Vanhoutte, EK, Bakkers, M, van Doorn, PA, Merkies, IS, Faber, CG, and PeriNom, SSg. 2011. "Revised normative values for grip strength with the Jamar dynamometer." *J Peripher Nerv Syst* 16 (1):47-50.

Razzaque, MS, and Lanske, B. 2007. "The emerging role of the fibroblast growth factor-23-klotho axis in renal regulation of phosphate homeostasis." *J Endocrinol* 194 (1):1-10.

Shane, E, Parisien, M, Henderson, JE, Dempster, DW, Feldman, F, Hardy, MA, Tohme, JF, Karaplis, AC, and Clemens, TL. 1997. "Tumor-induced osteomalacia: clinical and basic studies." *J Bone Miner Res* 12 (9):1502-11.

Stavrianeas, NG, and Kakepis, ME. 2004. "Epidermal nevus syndrome." *Orphanet Encyclopedia*.

Sullivan, W, Carpenter, T, Glorieux, F, Travers, R, and Insogna, K. 1992. "A prospective trial of phosphate and 1,25-dihydroxyvitamin D3 therapy in symptomatic adults with X-linked hypophosphatemic rickets." *J Clin Endocrinol Metab* 75 (3):879-85.

Verge, CF, Lam, A, Simpson, JM, Cowell, CT, Howard, NJ, and Silink, M. 1991. "Effects of therapy in X-linked hypophosphatemic rickets." *N Engl J Med* 325 (26):1843-8.

Weidner, N, and Santa Cruz, D. 1987. "Phosphaturic mesenchymal tumors. A polymorphous group causing osteomalacia or rickets." *Cancer* 59 (8):1442-54.

Yamazaki, Y, Tamada, T, Kasai, N, Urakawa, I, Aono, Y, Hasegawa, H, Fujita, T, Kuroki, R, Yamashita, T, Fukumoto, S, and Shimada, T. 2008. "Anti-FGF23 neutralizing antibodies show the physiological role and structural features of FGF23." *J Bone Miner Res* 23 (9):1509-18.

10 SIGNATURE PAGE

Protocol Title: A phase 2 open-label trial to assess the efficacy and safety of KRN23, an antibody to FGF23, in subjects with tumor-induced osteomalacia (TIO) or epidermal nevus syndrome (ENS-associated osteomalacia)

Protocol Number: UX023T-CL201 (Amendment 6)

I have read Protocol UX023T-CL201 (Amendment 6). I agree to conduct the study as detailed in this protocol and in compliance with the Declaration of Helsinki, Good Clinical Practice (GCP) and all applicable regulatory requirements and guidelines.

Investigator Signature　　　　　　　　　　　　　　　　Date

Printed Name:______________________________

Accepted for the Sponsor:

As the Sponsor representative, I confirm that Ultragenyx will comply with all Sponsor obligations as detailed in all applicable regulations and guidelines. I will ensure that the Investigator is informed of all relevant information that becomes available during the conduct of this study. PPD

PPD

Javier San Martin, MD　　　　　　　　　　　　　　　　Date
Senior Vice President,
Global Clinical Development
Ultragenyx Pharmaceutical Inc.
